# The Evolution of Fungicide Resistance Resulting from Combinations of Foliar-Acting Systemic Seed Treatments and Foliar-Applied Fungicides: A Modeling Analysis

**DOI:** 10.1371/journal.pone.0161887

**Published:** 2016-08-29

**Authors:** James L. Kitchen, Frank van den Bosch, Neil D. Paveley, Joseph Helps, Femke van den Berg

**Affiliations:** 1 Computational and Systems Biology, Rothamsted Research, Harpenden, Hertfordshire, United Kingdom; 2 Plant Pathology Department, ADAS, High Mowthorpe, Duggleby, North Yorkshire, United Kingdom; Charles University in Prague, CZECH REPUBLIC

## Abstract

For the treatment of foliar diseases of cereals, fungicides may be applied as foliar sprays or systemic seed treatments which are translocated to leaves. Little research has been done to assess the resistance risks associated with foliar-acting systemic seed treatments when used alone or in combination with foliar sprays, even though both types of treatment may share the same mode of action. It is therefore unknown to what extent adding a systemic seed treatment to a foliar spray programme poses an additional resistance risk and whether in the presence of a seed treatment additional resistance management strategies (such as limiting the total number of treatments) are necessary to limit the evolution of fungicide-resistance. A mathematical model was developed to simulate an epidemic and the resistance evolution of *Zymoseptoria tritici* on winter wheat, which was used to compare different combinations of seed and foliar treatments by calculating the fungicide effective life, i.e. the number of years before effective disease control is lost to resistance. A range of parameterizations for the seed treatment fungicide and different fungicide uptake models were compared. Despite the different parameterizations, the model consistently predicted the same trends in that i) similar levels of efficacy delivered either by a foliar-acting seed treatment, or a foliar application, resulted in broadly similar resistance selection, ii) adding a foliar-acting seed treatment to a foliar spray programme increased resistance selection and usually decreased effective life, and iii) splitting a given total dose—by adding a seed treatment to foliar treatments, but decreasing dose per treatment—gave effective lives that were the same as, or shorter than those given by the spray programme alone. For our chosen plant-pathogen-fungicide system, the model results suggest that to effectively manage selection for fungicide-resistance, foliar acting systemic seed treatments should be included as one of the maximum number of permitted fungicide applications.

## Introduction

Foliar plant diseases can cause significant yield loss. In wheat, for example, the disease Septoria tritici leaf blotch, caused by the fungus *Zymoseptoria tritici*, can reduce grain yields by up to 30–40% [1]. To reduce disease-induced yield loss, growers apply fungicides that reduce the rate of epidemic progress.

Fungicides can be applied directly to the foliage of the crop, protecting it from infection by fungal spores. Systemic fungicides, which are taken up by the plant and distributed through the leaf and other tissues, reduce pathogen infection, increase the pathogen’s latent period and reduce spore production. This makes them particularly effective at slowing down epidemics. Such fungicides can in some cases also be added to a seed coating. After germination the fungicide is then gradually taken up by the plant and distributed into the growing leaves. Therefore, the fungicide from a seed coating treatment is more active in the lower part of the canopy as compared to the fungicides applied with foliar sprays later in the crop growing season. Seed treatments with foliar-active systemic action can be attractive because they provide additional disease control and allow some flexibility in the subsequent foliar treatments program. For example, a seed treatment can provide insurance if an early foliar treatment is delayed or missed [2].

Fungicides affect one or more biochemical pathways in the pathogen hampering its growth. The QoI fungicides, for example, affect mitochondrial respiration, thereby shutting down the pathogen’s energy source. Fungicides that affect the same enzyme (target site) within a biochemical pathway are said to have the same mode-of-action (MOA). Fungal pathogens can develop resistance/insensitivity to a MOA, rendering the active substances within that group of fungicides less effective. Such resistance develops due to one or more mutation (or other genetic changes) in the pathogen’s genome. Systemic fungicides, which usually act against a single target site, are more prone to resistance than non-systemic fungicides which often act against multiple sites [3]. For example, one single mutation in the mitochondrial genome causes complete resistance to all QoI fungicides [4]. To prevent or at least delay the build-up of resistance in pathogen populations manufacturers in collaboration with regulatory authorities often put resistance management measures in place when a fungicide is introduced onto the market. Resistance management normally is a set of rules for the treatments program. For example, the number of applications per growing season can be restricted or the fungicide is marketed as a mixture with a fungicide with another mode of action [5].

For foliar application programs there is a considerable body of evidence showing that application programs have an effect on selection for fungicide resistance. Both laboratory and field experiments (summarized in [6]) and modelling studies (summarized in van den Bosch and Gilligan [7]) are published. This body of evidence has led to a clear set of recommendations on resistance management [8, 9]. For example, it is now generally accepted that mixing two fungicides with different modes of action reduces the rate of build-up of resistance.

Surprisingly little is known about the selection pressure imposed by seed treatments. Only one study has been published on the rate of fungicide resistance development due to seed treatments [10] as compared to over 70 experimental studies on foliar treatments [6]. Moreover, virtually nothing is known about the selection pressure due to seed treatments as compared to foliar treatments. The temporal and spatial differences in the activity of seed compared to foliar treatments, as discussed above, may lead to significantly differences in selection pressure. However, based on results from the only published experiment on the development of resistance to triademinol in powdery mildew of barley [10] concluded that the selection pressures were similar.

It is of key importance in the development of effective resistance management programs to understand and be able to predict the relative rate of selection for fungicide resistance by foliar and seed treatments. Manufacturers will continue to develop and register seed treatments when they add to effective disease control and are cost effective. A case in point is the recent development of the SDHI fungicides that can be applied as foliar as well as seed treatments. A decision needed to be made on resistance management for fungicide programs incorporating seed treatments. The Fungicide Resistance Action Committee (FRAC) decided that a seed treatment used against any foliar pathogen should be counted as one of the maximum two SDHI treatments per growing season [11]. This quite restrictive decision was taken in order to be precautionary in protecting future activity of this MOA group, given the lack of evidence.

The objective of this project was to build a model and use it to analyze the development of fungicide resistance with both foliar and seed treatments and to address the knowledge gaps described above. We parameterized the model for Septoria leaf blotch controlled by a fungicide representative of the SDHIs. In developing this model we ran into two significant problems. Firstly, there is little quantitative information in the literature about how plants take up fungicides from the seed coating. Secondly, few experiments on the efficacy of seed treatments against foliar pathogens are published, and none have been published on the efficacy of foliar-acting SDHI fungicides. This makes parameterizing the seed treatment elements of the model difficult. We addressed these problems as follows:

1. We developed a model incorporating two very different methods of fungicide uptake from the seed coating. Each can be switched on or off.
2. We developed two widely contrasting parameterizations for the seed treatment dynamics.

Each question posed to the model was then analyzed for all four combinations of uptake mechanism and parameterization. Combined structural and numerical sensitivity analyses were conducted to show how critically results depend on the assumptions regarding the uptake mechanism and parameter values. The two main questions we focus on in this paper are:

1. Does adding a seed treatment to the currently allowed two foliar applications affect resistance development more or less than adding a third foliar application?
2. Does using a seed treatment instead of one of the foliar applications affect resistance development differently as compared to using two foliar applications?

## Materials and Methods

### Model Overview

The model was used to simulate a population of the pathogen *Zymoseptoria tritici*, comprised of a fungicide-sensitive and a fungicide-resistant strain on winter wheat under selection pressure from a fungicide treatment program comprising a foliar-acting seed treatment and/or foliar applied treatments.

### The Canopy

The wheat crop canopy growth model developed is an extension of the model described by van den Berg, van den Bosch [12] and was used to simulate the growth and senescence of eleven explicit leaf layers (between nine and fourteen layers can be produced depending on sowing date and environment [13]). Following the convention in previous resistance articles the flag leaf as was numbered as leaf 1 and the bottom leaf as leaf 11. The leaf area of each leaf layer was measured in units of area index, defined as the ratio of the total leaf area to ground area [13].

**Fig 1:**
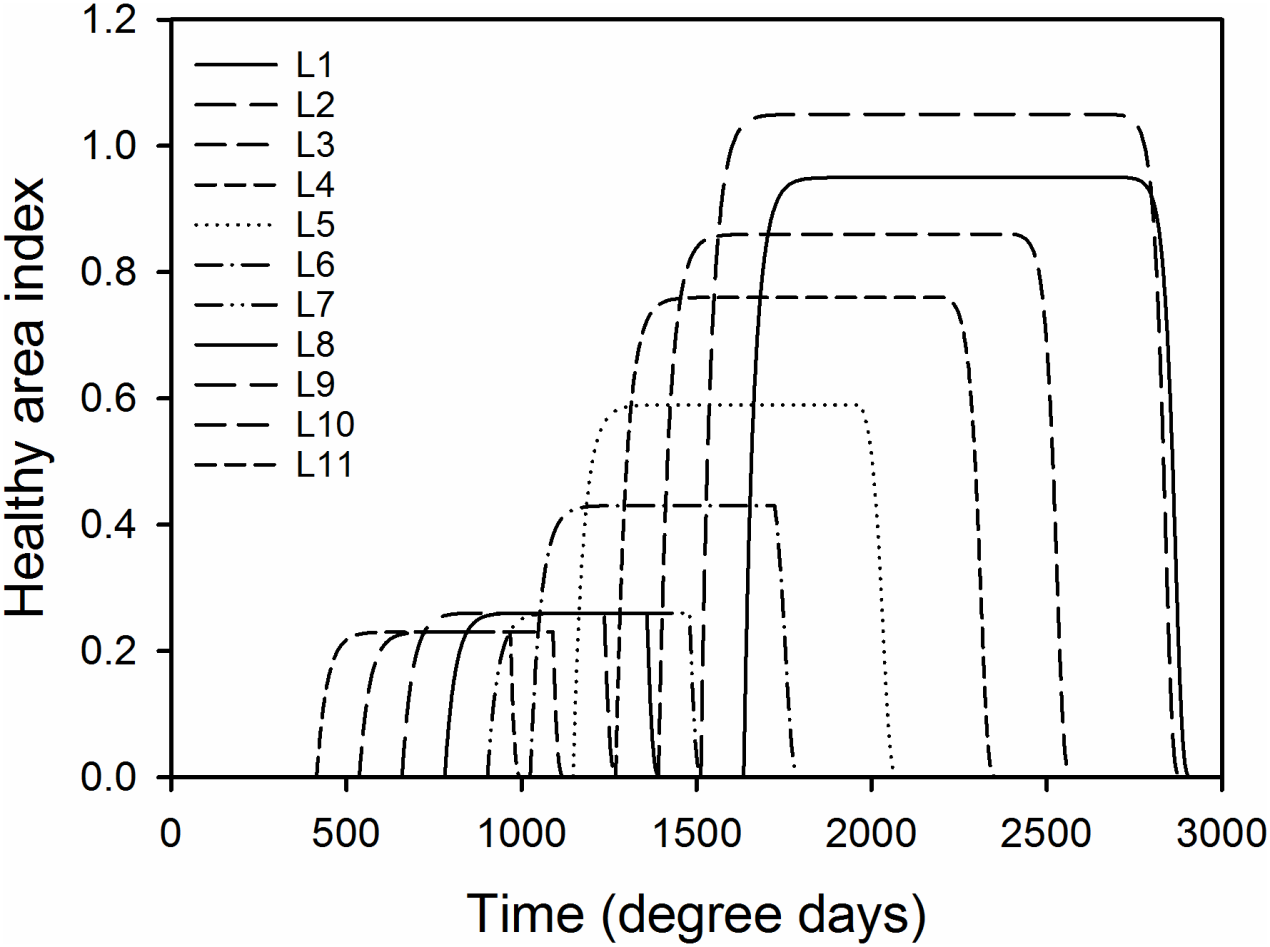
Graphical representation of the 11 leaves in the crop canopy growth model in the absence of disease. Leaf lives overlap and were comprised of a monomolecular growth phase, a lag phase and a logistic senescence phase.

The life cycle of each leaf layer *i* contained three phases: a growth phase, where leaf area increased according to a monomolecular function to a maximum; a lag phase, where leaf area remained constant, and a senescence phase, where leaf area decreased due to necrosis (Fig 1). Dead leaf tissues were removed from the simulation.

### The Pathogen

**Fig 2:**
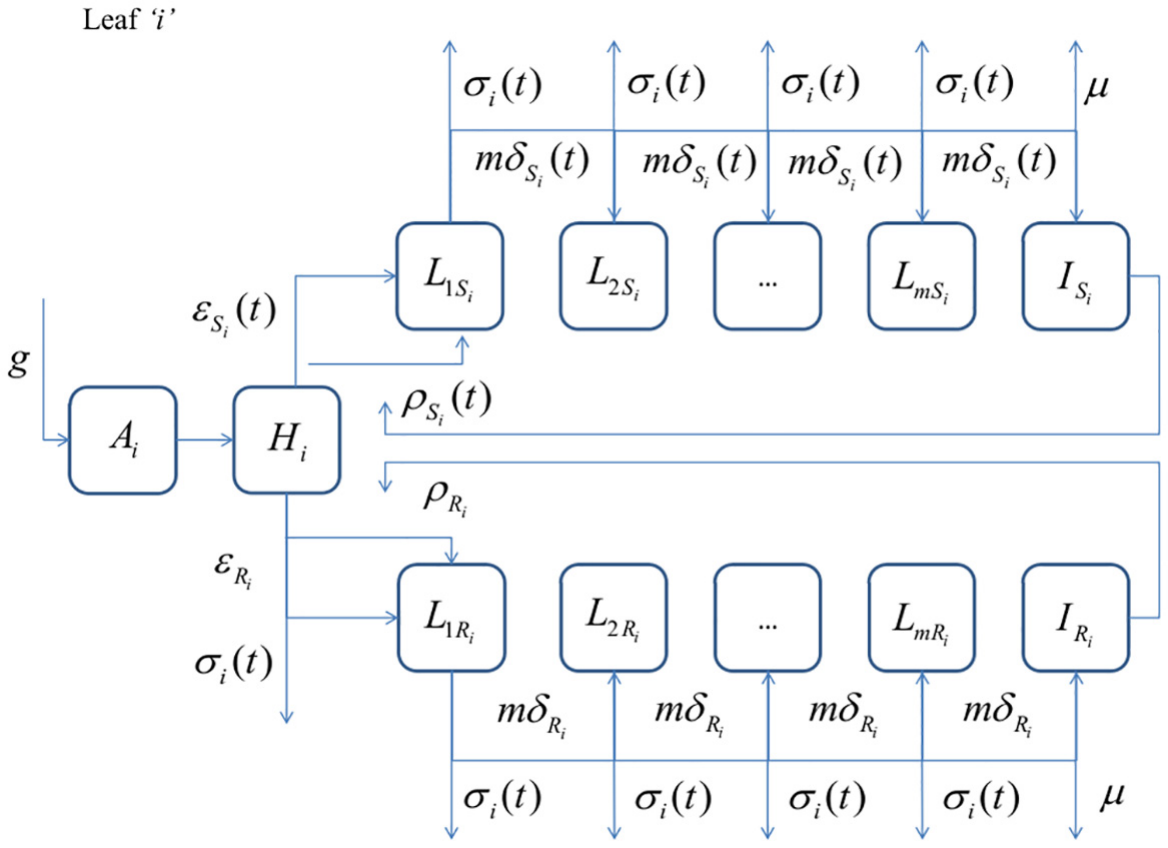
Flow diagram of the disease sub-model for leaf layer *i*. Healthy leaf area not lost by senescence (rate *σ*(*t*)) was infected at rate *ε* by primary inoculum (ascospores) and at rate *ρ* from secondary inoculum (pycnidiospores). The mean infectious period was 1/μ and the mean latent period was 1/ *δ*. The pathogen model included two strains; a fungicide sensitive strain, *S*, and a fungicide resistant strain, *R*. Parameters *δ*, *ρ* and *ε* for the sensitive strain were reduced according to the fungicide dose response functions.

The pathogen was modelled using compartments of latently infected tissue, comprised of non-sporulating mycelium, and infectious tissue, carrying sporulating fruiting bodies (Fig 2). At the start of the crop growing season the epidemic was initiated by primary inoculum, made up of asco-spores, produced by ascii on dead stuble left in the field after the previous season’s harvest. The primary inoculum gave rise to lesions producing pycnidiospores. During the rest of the crop growing season the pycnidiospores produced a series of asexual generations that formed the secondary inoculum of the epidemic. This secondary inoculum was dispersed by rain splash. Transmission of inoculum between leaf layers was reduced through stem extension, which led to a greater separation between the leaf layers.

### The Fungicides

Latent and infectious tissues belonged to either a fungicide-sensitive or a fungicide-resistant strain of the pathogen. The latter strain was assumed to be unaffected by the fungicide within the range of doses permitted and infected new leaf tissues at the same rate in the presence or absence of the fungicide.

Use of the fungicide caused selection for the fungicide-resistant strain. The fungicide was applied as a T1 and/or a T2 foliar spray, applied at the full emergence of the third eventual leaf or the flag leaf, respectively, or as a foliar-acting seed treatment. The fungicide was systemic and exhibited both protectant (transmission rate limiting) and eradicant (latent period prolonging) activity towards the fungicide-sensitive strain.

The systemic seed treatment fungicide was initially present on, or around, the seed and then gradually translocated to the leaves. The fungicide concentration present in each leaf decayed exponentially over time due to breakdown from exposure to light and plant catabolism.

### Fungicide Effective Life

To compare the effect of selection pressure between foliar sprays and systemic seed treatments, we used the fungicide effective life. The effective life is the number of consecutive years the fungicide treatment program was able to maintain effective disease control. Hereby, loss of effective disease control was defined as a reduction in healthy area duration (HAD), as measured on leaves 1–3, greater than 5%. We used this quantification of effective disease control because for wheat HAD is closely correlated with yield [14].

### Model Description

#### Plant growth dynamics

We denote by subscript *i* the *i*^*th*^ leaf layer. Leaf layers emerged in reverse chronological order, thus leaf layer *i* = 11 was the first leaf layer to emerge and leaf layer *i* = 1 was the flag leaf layer. Each leaf layer began growth at *t = t*_*initiation_i*_, was fully emerged at *t = t*_*emergence_i*_, began senescence at *t = t*_*senes_i*_ and was removed from the simulation at *t = t*_*death_i*_. The total leaf layer area index, *A*_*i*_, grew according to a monomolecular function [15] at rate *g* and reached an asymptote at the maximum area index for leaf layer *i*, *A*_max_*i*_:

$$\frac{dA_{i}}{dt}=\{\begin{array}{ll} 0 & t<t_{initiation\_i}\\ g(A_{\text{max}\_i}-A_{i}) & t<t_{senes\_i}\\ 0 & t\geq t_{death\_i} \end{array}$$ (1)

The healthy area index (HAI) of each leaf layer, *H*_*i*_, was comprised of infection-free photosynthetic leaf tissue and in the absence of disease was equal to *A*_*i*_ up until *t* = *t*_*_senes_i*_, after which *H*_*i*_ decreased at rate *σ*_*i*_(*t*) until *t = t*_*death_i*_:

$$\frac{dH_{i}}{dt}=\{\begin{array}{ll} 0 & t<t_{initiation\_i}\\ \frac{dA_{i}}{dt} & t\leq t<t_{senes\_i}\\ -\sigma_{i}(t)H_{i} & t_{senes\_i}\leq t<t_{death\_i} \end{array}$$ (2)

The senescence rate of leaf layer *i*, *σ*_*i*_(*t*), was calculated by:

$$\sigma_{i}(t)=e^{s(t-t_{death\_i})}\;t_{senes\_i}\leq t<t_{death\_i}$$ (3)

leading to a sigmoidal decline until *t*_*death*_*i*_, at which point the leaf layer was removed from the simulation.

#### Pathogen dynamics

**Fig 3:**
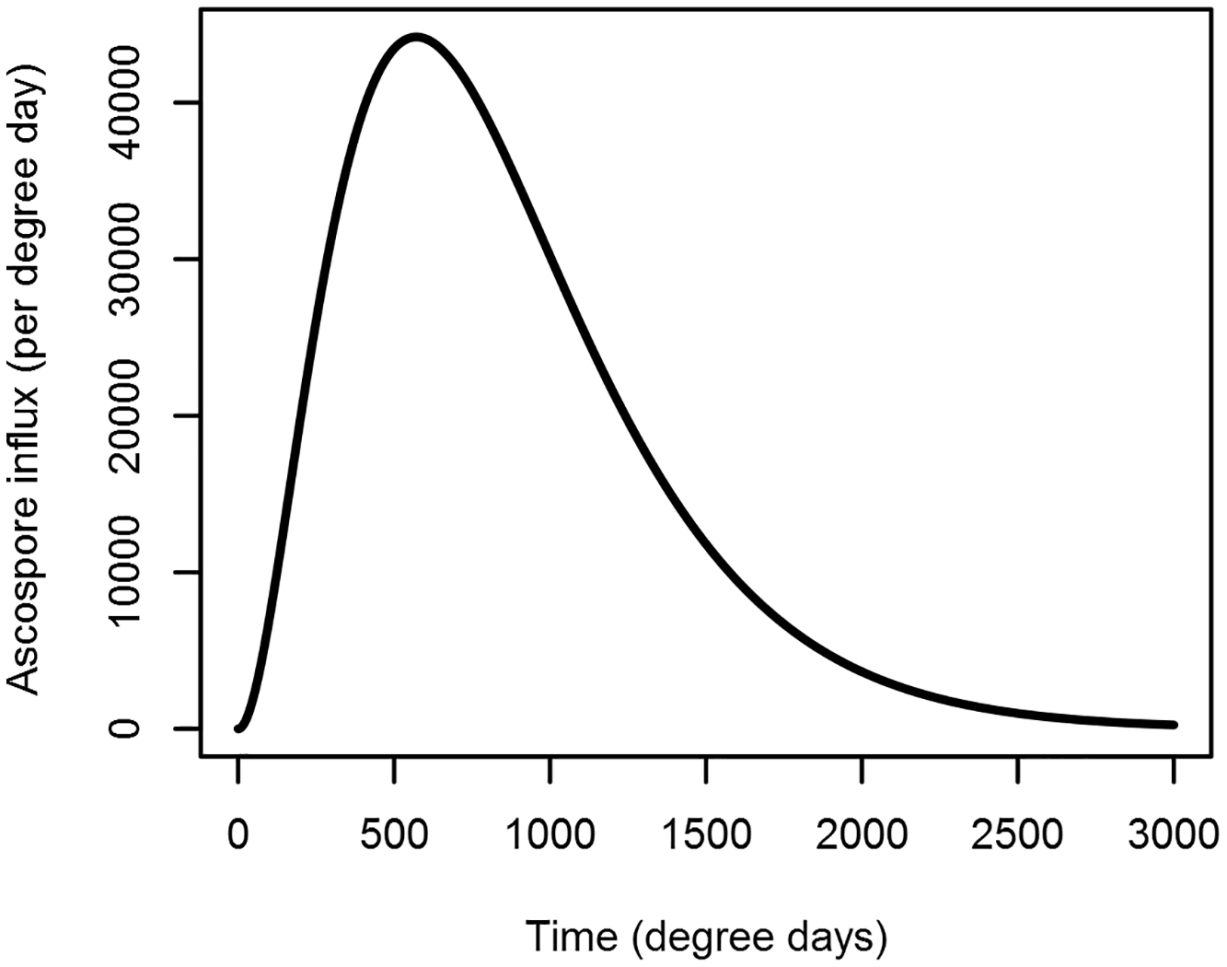
Seasonal ascospore spore concentrations. The peak of the function was during the winter (0–1200 degree days) and declined to 1% of the peak value at the time of death of leaf 5 (2094 degree days).

The epidemic was initiated through infection by wind-blown ascospores of the fungicide-sensitive and fungicide-resistant strains, which were produced by pseudothecia on plant debris. A flow diagram of the epidemic model is given in Fig 2. The rate of influx of ascospores, *X*(*t*), is given by:

$$X(t)=\eta t^{2}e^{-\lambda t}$$ (4)

where *η* and *λ* are parameters (Fig 3). When multiplied with the ascospore deposition rate and the ascospore infection efficiency, *γ*, our ascospore transmission rate, *ε*(*t*), was obtained:

ε(t)=γX(t) (5)

The transmission rate of fungicide-sensitive ascospores was reduced according to the concentration of fungicide at time *t* on leaf layer *i* and was thus denoted by $\varepsilon_{S_{i}}(t)$. It was assumed that fungicide-resistant ascospores were fully resistant to the fungicide and the transmission rate of fungicide-resistant ascospores was denoted by $\varepsilon_{R_{i}}$.

Parameter *θ*_0_ denotes the initial proportion of ascospores which were resistant to the fungicide. During the simulation, the proportion of fungicide-resistant ascospores released from pseudothecia on plant debris, *θ*, was calculated at the end of every growing season according to the fraction of fungicide-resistant infectious leaf tissue on the top five leaves:

$$\theta =\frac{\sum_{i=1}^{5}I_{R_{i}}}{\sum_{i=1}^{5}I_{S_{i}}+\sum_{i=1}^{5}I_{R_{i}}}$$ (6)

$I_{S_{i}}$ and $I_{R_{i}}$ are the infectious leaf tissue occupied by fungicide-sensitive and fungicide resistant infectious lesions, respectively.

Once initial infection as a result of influx of ascospore comprising primary inoculum had occurred the epidemic was driven by infection from pycnidiospores arising from mycelium on the remaining active leaf layers. Each infectious lesion produced a constant number of pycnidiospores per time unit. The transmission rate parameter for pycnidiospores, *ρ*, was found by multiplying the spore production rate with the infection efficiency. The transmission rate for fungicide-sensitive pycnidiospores was reduced according to the fungicide concentration on leaf layer *i* at time *t* and was denoted by $\rho_{S_{i}}(t)$, whereas the transmission rate for fungicide-resistant pycniodiospores on leaf layer *i* was unaffected by fungicide and was denoted by $\rho_{R_{i}}$. Unlike wind-dispersed ascospores, pycnidiospores were splash dispersed between leaf layers [16], and the number of pycnidiospores that were dispersed between leaf layers was reduced by the distance between the layers. Stem extension only occured between leaf layers 1 to 4. We denoted the probability of splash dispersal between leaf layer *j* and leaf layer *i* as *P*_*j*,*i*_(*D*_*j*,*i*_), which was a function of the distance between leaf layers *j* and *i*, *D*_*j*,*i*_:

$$P_{j,i}(D_{j,i})=\{\begin{array}{ll} 1 & i=j\\ e^{-\sigma_{down}D_{j,i}} & i>j\\ e^{-\sigma_{up}D_{j,i}} & i<j \end{array}$$ (7)

where *σ*_*down*_ and *σ*_*up*_ were parameters for the ease of downward and upward splash dispersal, respectively. For each leaf layer *i*, *P*_*j*,*i*_(*D*_*j*,*i*_) was summed over every active leaf layer *j*. We denoted the number of active leaf layers for any given *t* as *u*. By including both sources of inoculum, Eq (2) was extended to:

$$\frac{dH_{i}}{dt}=\{\begin{array}{ll} 0 & t<t_{initiation\_i}\\ \frac{dA_{i}}{dt}-\left(\frac{H_{i}}{A_{i}}\right)\left(\rho_{S_{i}}(t)\sum_{j=1}^{u}P_{j,i}(D_{j,i})I_{S_{j}}+\rho_{R_{i}}\sum_{j=1}^{u}P_{j,i}(D_{j,i})I_{R_{j}}+(1-\theta )\varepsilon_{S_{i}}(t)+\theta \varepsilon_{R_{i}}\;\right) & t_{initiation\_i}\leq t<t_{senes\_i}\\ -\left(\frac{H_{i}}{A_{i}}\right)\left(\rho_{S_{i}}(t)\sum_{j=1}^{u}P_{j,i}(D_{j,i})I_{S_{j}}+\rho_{R_{i}}\sum_{j=1}^{u}P_{j,i}(D_{j,i})I_{R_{j}}+(1-\theta )\varepsilon_{S_{i}}(t)+\theta \varepsilon_{R_{i}}\;\right)-\sigma_{i}(t)H_{i} & t_{senes\_i}\leq t<t_{death\_i} \end{array}$$ (8)

Latently infected leaf tissue transitioned to infectious lesions at rate *δ* and was reduced due to the eradicant properties of the fungicide on leaf layer *i* at time *t*. Hence, the rate with which latently infected leaf tissue transitioned to infectious lesions was denoted by $\delta_{S_{i}}(t)$ and $\delta_{R_{i}}$ for the fungicide-sensitive and fungicide-resistant pathogen strains, respectively. Following van den Berg, van den Bosch [12] and Cunniffe, Stutt [17] we introduced *m* latently infected compartments. This resulted in a more realistic gamma distributed latent period compared to the exponentially distributed latent period that would have arisen from one compartment [17]. The rate at which latently infected leaf tissue transitioned between the *m* latent compartments for the fungicide-sensitive and resistant strains was hence $m\delta_{S_{i}}(t)$ and $m\delta_{R_{i}}$, respectively. Like healthy leaf tissue, latently infected leaf tissue senesced at rate *σ*_*i*_(*t*). Eqs 9 & 10 define our differential equations for the growth of the first latently infected leaf tissue compartment for the fungicide-sensitive and fungicide-resistant strain, respectively:

$$\frac{dL_{1S_{i}}}{dt}=\left(\frac{H_{i}}{A_{i}}\right)\left(\rho_{S_{i}}(t)\sum_{j=1}^{u}P_{j,i}(D_{j,i})I_{S_{j}}+(1-\theta )\varepsilon_{S_{i}}(t)\right)-L_{1S_{i}}\left(m\delta_{S_{i}}(t)+\sigma_{i}(t)\right)$$ (9)

$$\frac{dL_{1R_{i}}}{dt}=\left(\frac{H_{i}}{A_{i}}\right)\left(\rho_{R_{i}}\sum_{j=1}^{u}P_{j,i}(D_{j,i})I_{R_{j}}+\theta \varepsilon_{R_{i}}\;\right)-L_{1R_{i}}\left(m\delta_{R_{i}}+\sigma_{i}(t)\right)$$ (10)

The differential equations for each remaining latently infected leaf tissue compartment *n* where *n* ϵ {2, …, *m*} are defined for the fungicide-sensitive and fungicide-resistant strain in Eqs 11 and 12.

$$\frac{dL_{nS_{i}}}{dt}=m(L_{(n-1)S_{i}}-L_{nS_{i}})\delta_{S_{i}}(t)-\sigma_{i}(t)L_{nS_{i}}$$ (11)

$$\frac{dL_{nR_{i}}}{dt}=m(L_{(n-1)R_{i}}-L_{nR_{i}})\delta_{R_{i}}-\sigma_{i}(t)L_{nR_{i}}$$ (12)

Infectious leaf tissue of the fungicide-sensitive Eq (11) and fungicide-resistant Eq (12) strains grew due to the influx of latently infected leaf tissue from latent compartment *m* at rate $m\delta_{S_{i}}(t)$ and $\;m\delta_{R_{i}}$ and was removed at rate *μ*:

$$\frac{dI_{S_{i}}}{dt}=m\delta_{S_{i}}(t)L_{mS_{i}}-\mu I_{S_{i}}$$ (13)

$$\frac{dI_{R_{i}}}{dt}=m\delta_{R_{i}}L_{mR_{i}}-\mu I_{R_{i}}$$ (14)

#### Foliar spray dynamics

The dose of the fungicide that was intercepted by leaf layer *i*, *F*_*i*_, at *t* = *t*_*spray*,_ was measured in units of mg m^-2^ of leaf area. In analogy with the transmission of light through a turbid medium (Beer-Lambert law), the dose intercepted by leaf layer *i* increased with its AI and decreased according to the product of the AI of each leaf layer *j* above that intercepts the sprayed fungicide:

$$F_{i}=F_{0}\left(1-e^{-\tau A_{i}(t_{spray})}\right)\left(\prod_{j=1}^{i-1}e^{-\tau A_{j}(t_{spray})}\right)\;t_{initiation\_j}\leq t<t_{death\_j}$$ (15)

where *τ* was the angle of leaf layer *i*, measured as a projection onto a horizontal surface, which ranged from zero when fully vertical to 1 at a fully horizontal projection. *F*_*0*_ was the total dose in mg m^-2^ of ground that was sprayed onto the field at *t* = *t*_*spray*_. The fungicide concentration within leaf layer *i*, measured in mg m^-3^ of leaf volume at time unit *t* − *t*_*spray*,_ equal to *f*_*foliar*_*i*_(*t*), was then calculated by:

$$f_{foliar\_i}(t)=\frac{F_{i}}{qA_{i}}e^{-\upsilon (t-t_{spray})}$$ (16)

where *q* represented leaf thickness, and *υ* was the fungicide breakdown rate.

#### Seed treatment dynamics

We modelled a continuous flow of systemic fungicide from the treated seed coating into the leaf layers where it accumulated and subsequently decayed. We did not simulate export of fungicides from leaf layers. The initial seed treatment fungicide dose was a quantity in mg per seed. The flow of seed treatment fungicide from the seed coating into each leaf layer was calculated in units of mg per time unit, and the dose that reached each leaf layer *i* was converted into a concentration of mg m^-3^ of leaf volume, which was then summed with the foliar spray concentration to determine the protectant and eradicant effects of the fungicide on the pathogen. The systemic fungicide that was within the seed reservoir, *N*_*seed*_, was depleted over time at rate *β*(*t*):

$$\frac{dN_{seed}}{dt}=-\beta (t)N_{seed}$$ (17)

As discussed in the introduction we used two models of seed treatment uptake. In the first model we assumed a constant rate of uptake, *β*(*t*) = *β*. In the second model, the seed treatment fungicide was drawn through the plant xylem resulting from a transpirational pull at rate *β*(*t*), which was a sigmoidal function of time:

$$\beta (t)=b+\frac{yce^{at}}{ce^{at}+(1-c)}$$ (18)

where *b* and *y* are parameters for the baseline and maximum transpiration rate, respectively, and *c* and *a* are growth parameters. We assumed that the influx of seed treatment fungicide from the seed coating into each leaf layer *i*, *z*_*i*,_ was proportional to the fraction of the leaf layer’s healthy leaf tissue, *H*_*i*_, relative to the total healthy leaf tissue across all leaf layers, which was equal to:

$$z_{i}=\beta N_{seed}\frac{H_{i}}{\sum_{j=1}^{u}H_{j}}\;t_{initiation\_j}\leq t<t_{death\_j}$$ (19)

**Fig 4:**
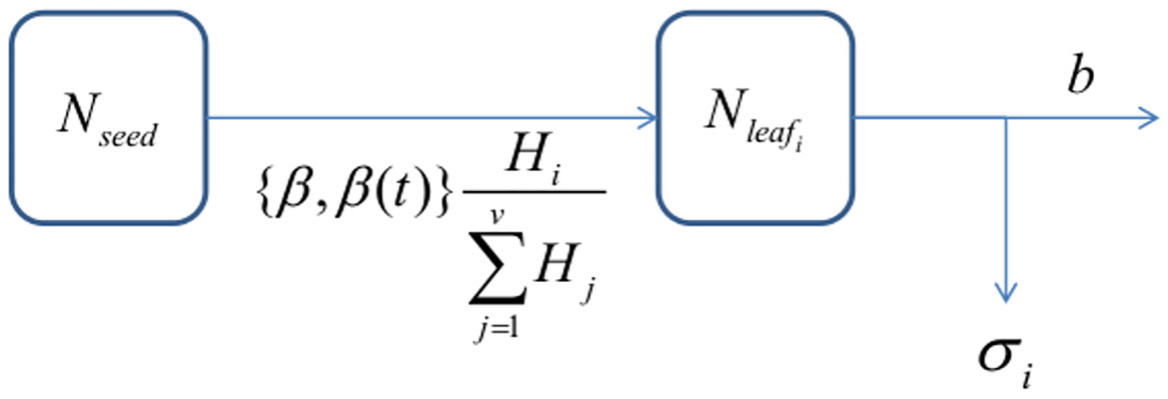
Flow diagram for the seed treatment fungicide sub-model. The fungicide in the seed coating (*N*_*seed*_) decreased at rate *β* and moved into each leaf according to the product of *β* and the proportion of the leaf’s healthy area index relative to the total canopy healthy area. Seed treatment loss from each leaf was due to senescence and catabolism, at rates *σ* and *υ* respectively.

The systemic fungicide dose accumulated in leaf *i*, *N*_*layer*_*i*_, increased at rate *z*_*i*_ and decreased from breakdown and leaf senescence at rates *b* and *σ*(*t*), respectively (Fig 4):

$$\frac{dN_{leaf\_i}}{dt}=z_{i}-bN_{leaf\_i}-\sigma (t)N_{leaf\_i}$$ (20)

The concentration of systemic fungicide applied as a seed treatment in mg per sown seed was given by:

$$f_{seed\_treatment\_i}=\frac{N_{leaf\_i}}{qh_{i}}$$ (21)

**Fig 5:**
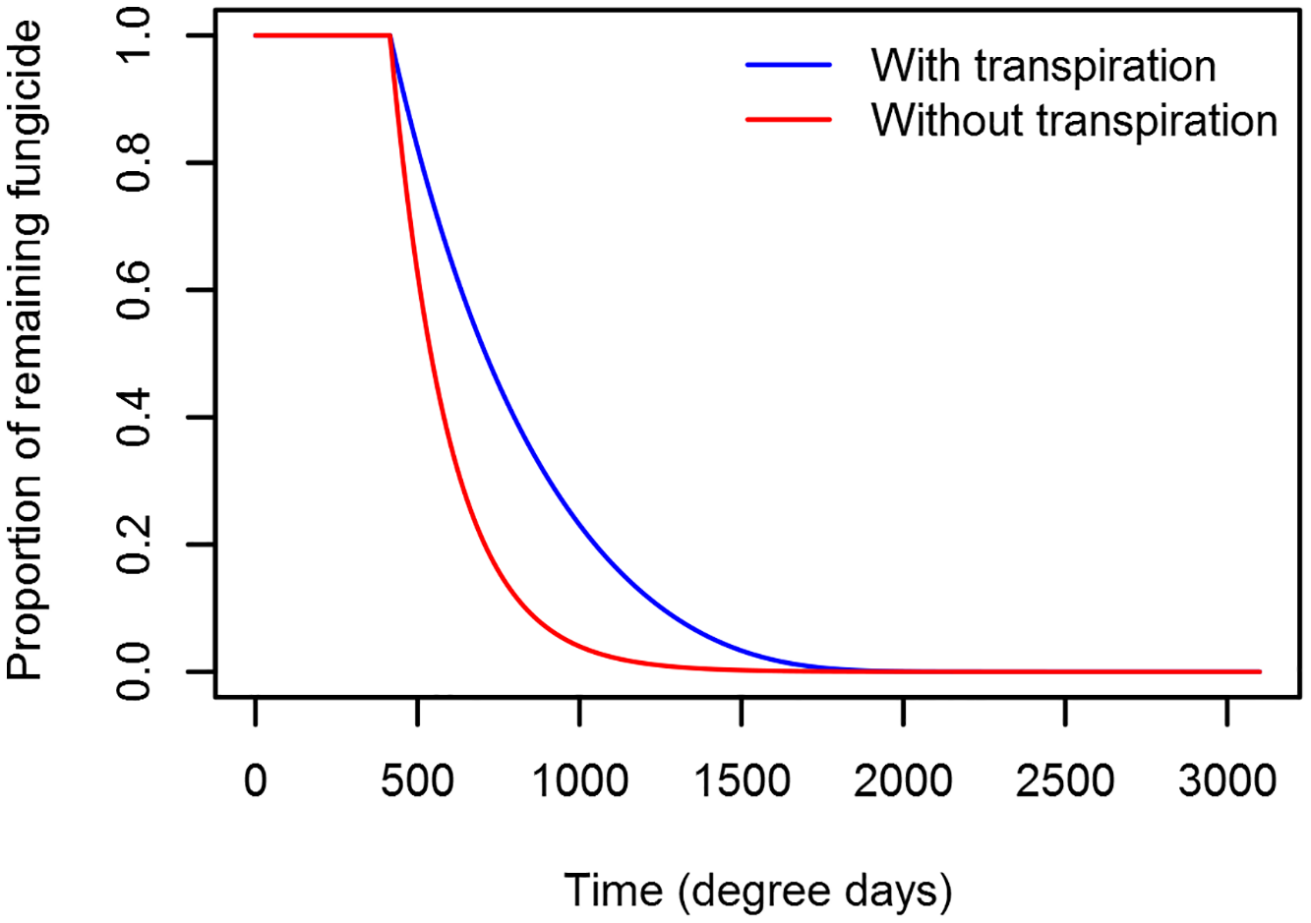
Seed treatment fungicide depletion for the constant versus transpiration-based seed treatment uptake model. Both models were parameterized such that 99.99% of the fungicide has been depleted from the seed at the time that leaf layer 5 is dead.

Where *h*_i_ is the total area in m^2^ for leaf *i* per m^2^ ground area and *q* is the leaf thickness in m. See Fig 5 for a graphical representation of how the two different seed treatment uptake models affect the seed treatment fungicide depletion.

#### Dose response curves

Our dose response equations were of exponential type and not traditional probit-log models [18], as the exponential curve fitted to the observed data [19]. The modelled fungicide had both protectant activity, which reduced the ascospore and pycnidiospore transmission rates *ε*_*i*_ and *ρ*_*i*_, and eradicant activity, which increased the length of the latent period, as given by 1 / *δ*_*i*_. However, the eradicant activity of systemic fungicides was only effective during the early latent stages of infection [20, 21], thus following the approach of van den Berg, van den Bosch [12] only lesions that are in the first half of the latent period are affected by the eradicant action of the fungicide (i.e. only latent compartments *n* that satisfy the condition 1 ≤ *n* ≤ (*m* / 2) are affected by the fungicide). The dose response functions had two parameters: *α*, which we defined as the maximum proportional reduction in the target pathogen parameter *ω*, where *ω* ϵ {*ρ*, *δ*, *ε*}; and *k*, which served as the dose response curve shape parameter.

$$\alpha_{\omega }(t)=\alpha_{\text{max}\_\omega }\left(1-e^{-k_{\omega }\left(f_{foliar\_i}(t)+f_{seed\_treatment}\right)}\right)$$ (22)

The pathogen parameters of the fungicide-sensitive strain were then deduced according to:

$$\rho_{S_{i}}(t)=\rho \left(1-\alpha_{\rho }(t)\right)$$ (23)

$$\varepsilon_{S_{i}}(t)=\varepsilon \left(1-\alpha_{\varepsilon }(t)\right)$$ (24)

$$\delta_{S_{i}}(t)=\{\begin{array}{ll} \delta \left(1-\alpha_{\delta }(t)\right) & in\;L_{n}\;with\;n\leq m/2\\ \delta & in\;L_{n}\;with\;n\;>\;m/2 \end{array}$$ (25)

**Fig 6:**
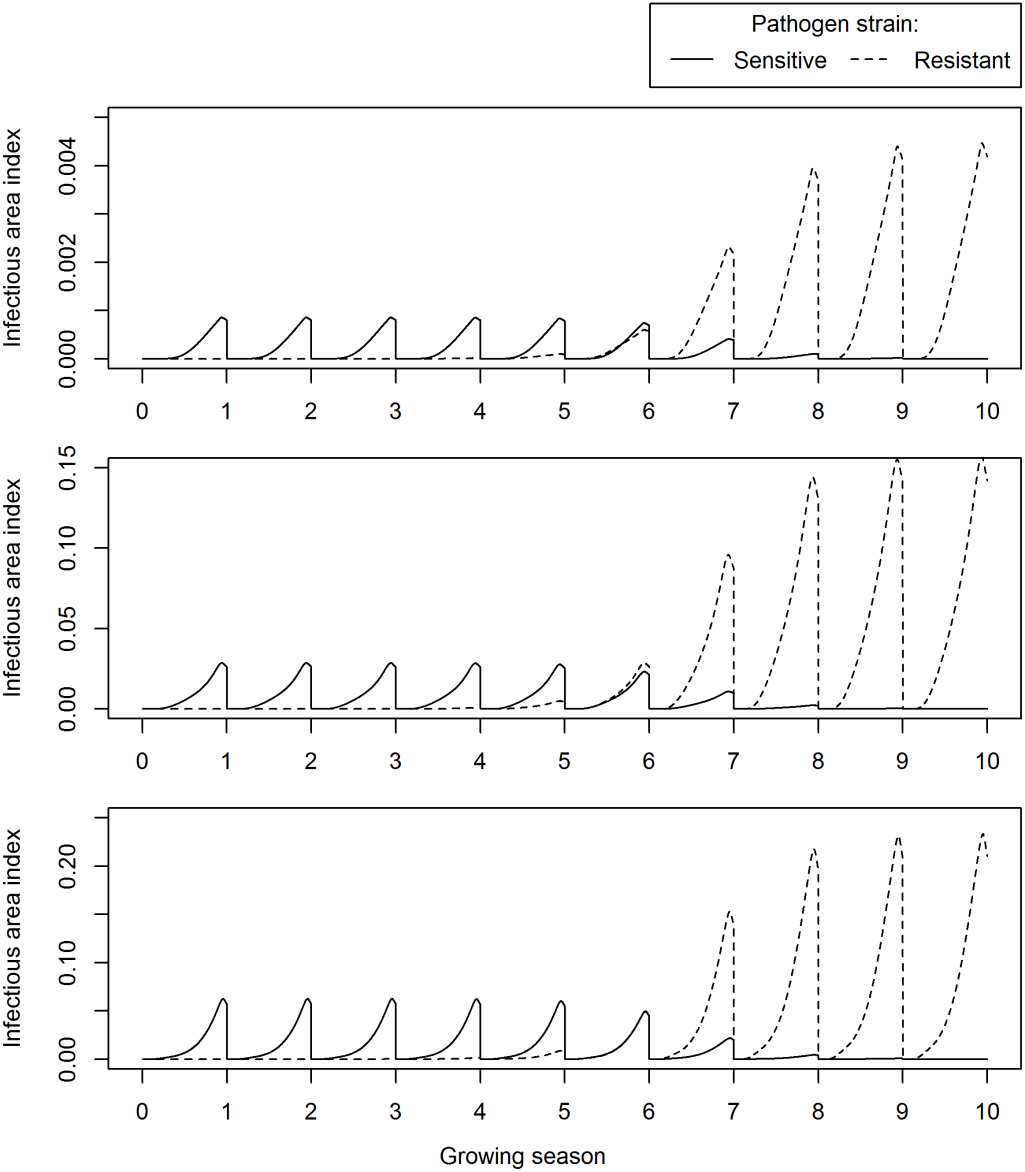
Time course of the sensitive and resistant pathogen strain for a T1 foliar treatment. The simulation was run for a low fungicide breakdown rate and a constant seed treatment uptake model. Blue and red lines indicate fungicide-sensitive and fungicide-resistant area index, respectively. Top panel: leaf layer 11. Middle panel: leaf layer 5. Bottom panel: leaf layer 1. A T1 spray was applied at 20 mg/m^2^ and the simulation was run for 10 growing seasons.

**Fig 7:**
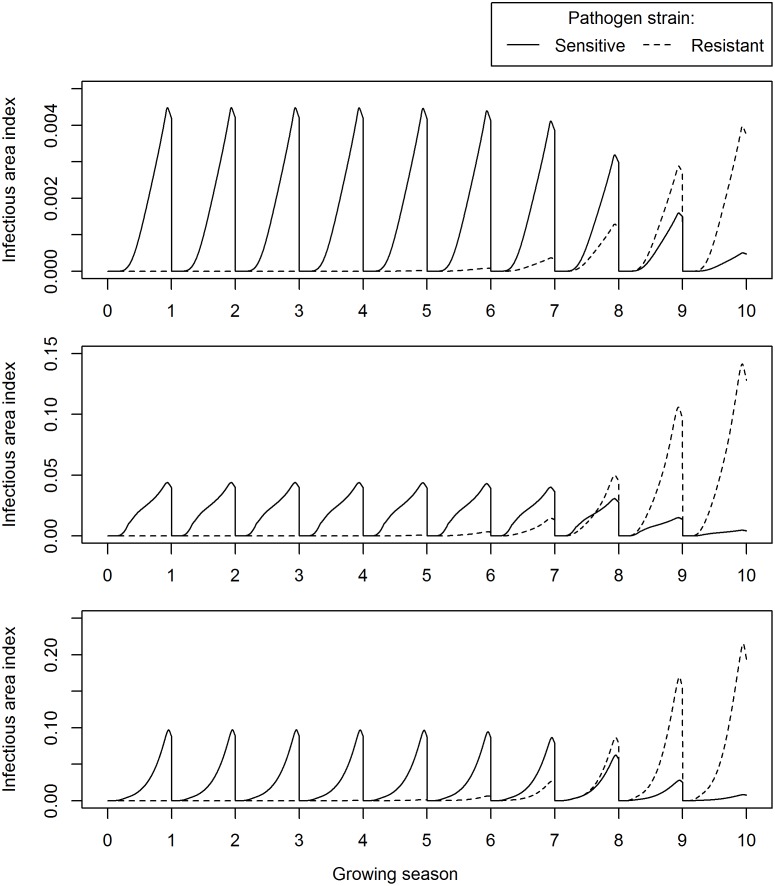
Time course of the sensitive and resistant pathogen strain for a seed treatment. The simulation was run for a low fungicide breakdown rate and a constant seed treatment uptake model. Blue and red lines indicate fungicide-sensitive and fungicide-resistance area index, respectively. Top panel: leaf layer 11. Middle panel: leaf layer 5. Bottom panel: leaf layer 1. A seed treatment was applied at 4.5 mg/m^2^, which provides a HAD gain that approximates that of a T1 spray, and the simulation was run for 10 growing seasons.

The target pathogen parameters were left unchanged for the fungicide-resistant strain, which we assumed to exhibit absolute resistance. Example time courses of the sensitive and resistant pathogen strains when fungicide treatments consist of a T1 foliar treatment only or a seed treatment only are given in Figs 6 and 7, respectively.

#### Definition of loss of effective control

To compare the selection for fungicide resistance within the simulated treatment regimens we calculated the fungicide effective life [7, 22, 23], which is defined as the number of years after first introduction over which the fungicide is able to maintain effective disease control. Hereto, we first calculated the healthy area duration (HAD) of the crop canopy [14] according to the equation:

$$HAD=\int_{t=2100}^{t=3100}\sum_{i=1}^{3}\left(H_{i}+\sum_{n=1}^{m}\left(L_{nS_{i}}+L_{nR_{i}}\right)\right)\;dt$$ (26)

The integral in Eq 26 was calculated using the healthy and latently infected leaf tissue of the top three leaf layers, from anthesis at Zadock’s GS61 until the end of the simulation at GS91. HAD was calculated numerically according to the method described in [24], as implemented in the NAG Numerical Library [25]. For wheat the HAD experienced during the yield forming period is closely correlated with final yield [14], and was used to determine whether a fungicide application was still controlling the epidemic. In the absence of disease we calculated a reference value, *HAD*_0_, and defined that a HAD loss of 5% or greater, relative to *HAD*_*0*_, indicated a loss of effective disease control. Either an inadequate fungicide programme (insufficient treatments and/or dose per treatment) or selection for fungicide-resistance will have caused effective disease control to fail. The effective life was then the number of consecutive growing seasons before disease control was lost.

### Parameter Values

For a detailed description of the estimation of all model parameters we refer the reader to S1 file. The parameter values are summarized in Table 1.

**Table 1:** Parameter symbols, descriptions, values and units.

Parameter name	Definition	Value	Units
*g*	Leaf growth rate	0.034	t^-1^
*s*	Leaf senescence rate	0.05	t^-1^
*τ*	Measure of leaf projection onto horizontal surface	0.77	Dimensionless
*t*_*anthesis_early*_	Time of early anthesis	2100	t
*t*_*harvest*_	Time of harvest	3100	t
*q*	Leaf thickness	0.001	m
*D*_*max*_	Maximum stem extension between leaves 1–4	10	cm
*η*	Ascospore influx coefficient	1	t^-3^
*λ*	Ascospore influx decay rate	0.0035	Dimensionless
*θ*_*0*_	Initial proportion of fungicide-resistance in population	1.00E-05	Dimensionless
*σ*_*up*_	Rate of change of reduction of inoculum from upward splash dispersal	0.1	Dimensionless
*σ*_*down*_	Rate of change of reduction of inoculum from downward splash dispersal	0.01	Dimensionless
*1/μ*	Infectious period	456	t
*1/δ*	Latent period	244	t
*m*	Number of latent compartments	10	Dimensionless
*γ*	Infection efficiency per individual ascospore	4.00E-10	HAI spore-1
*ε*	Ascospore transmission rate	Variable	HAI t^-1^
*ρ*	Pycnidiospore transmission rate	0.007	t^-1^
*υ*_*low*_	Low fungicide breakdown rate	0.0046	Dimensionless
*υ*_*high*_	High fungicide breakdown rate	0.009	Dimensionless
*β*	Seed treatment uptake rate	0.0055	t^-1^
*α*_*δ_high*_; *α*_*ε_high*_; *α*_*ρ_high*_;	Maximum reduction in pathogen parameters from fungicides with a high breakdown rate	0.5	Dimensionless
*α*_*δ_low*_; *α*_*ε_low*_; *α*_*ρ_low*_;	Maximum reduction in pathogen parameters from fungicides with a low breakdown rate	0.45	Dimensionless
*k*_*δ_high*_; *k*_*ε_high*_; *k*_*ρ_high*_;	Dose response curve shape parameter for fungicides with a high breakdown rate	0.003	Dimensionless
*k*_*δ_low*_; *k*_*ε_low*_; *k*_*ρ_low*_;	Dose response curve shape parameter for fungicides with a low breakdown rate	0.0025	Dimensionless
*b*	Intercept of transpiration based seed treatment uptake	0.002	t^-1^
*c*	Coefficient for transpiration based seed treatment uptake	0.0015	Dimensionless
*y*	Asymptote for transpiration based seed treatment uptake	0.027	t^-1^
*a*	Rate of increase for transpiration based seed treatment uptake	0.003	Dimensionless
*l*_*short*_	Number of leaf layers used for calculation of resistance proportion, with a short ascospore longevity	5	Dimensionless
*l*_*long*_	Number of leaf layers used for calculation of resistance proportion, with a long ascospore longevity	11	Dimensionless

Here we only describe the estimation of parameter values used to model the systemic seed treatment efficacy. The initial amount of fungicide in the seed coating, *N*_*seed*_, in units of mg seed^-1^ was estimated by searching for values that resulted in a specific level of disease control. In our simulations, we used two different parameterizations:

#### Parameterization 1

The initial seed treatment dose was set to provide the same antifungal effect as a T1 spray. This was achieved by adjusting *N*_*seed*_ until the calculated HAD at the end of the first growing season equaled the HAD value obtained from a T1 spray. This parameterization provided the upper bound for the disease control provided by a systemic seed treatment, as it is unlikely that a systemic seed treatment could provide a stronger reduction in disease severity than a T1 spray. The estimated doses varied according to the fungicide breakdown rate and the seed treatment uptake model in use (S1 Table).

#### Parameterization 2

A dataset was obtained from Parker and Lovell [26], which contained spore-washing data in spores ml^-1^ on leaves of winter wheat that were infected by Septoria leaf spot, comparing untreated plots with plots treated with a seed treatment (fluquinconazole; product name Jockey). Areas under the disease progress curve (AUDPC) values for the spore washing data were calculated by numerically integrating the spore washing values using the method described in [24], as implemented in [25]. The percentage AUDPC remaining after treatment was observed and recorded for each data point from both sets. Averaging over all data points for both sets yielded average reductions in AUDPC values of 60% after treatment. Values of *N*_*seed*_ were then adjusted in the model to obtain a 60% reduction in AUDPC (S2 Table).

## Results

### Selection for Fungicide-Resistance

The model was used to calculate fungicide-sensitive and fungicide-resistant infectious leaf tissue over ten growing seasons on leaf layers 1, 5 and 11 (representing the upper, mid- and lower canopy, respectively, after applying either a solo T1 foliar spray or a solo systemic seed treatment (Figs 6 and 7, respectively). The fungicide in these calculations was parameterized with a low breakdown rate, and a constant uptake model for the systemic seed treatment fungicide was used (refer to Table 1 for values). To provide an appropriate comparison between both treatments, the input seed treatment dose was parameterized to approximate the HAD gain of a T1 spray (S1 Table). As expected, applying a seed treatment led to the fungicide-sensitive infectious leaf tissue being reduced most in the lower canopy, particularly on leaf layer 11. However, the fungicide-sensitive infectious leaf tissue on leaf layers 1 and 5 were affected to a similar extent when treated with a systemic seed treatment as compared to a T1 foliar spray. There were no substantial differences in the growth of the fungicide-resistant strain after either treatment, indicating that the selection pressure for fungicide-resistance was similar in both cases.

To obtain a quantitative measure of the selection pressure, median selection ratios [23] were calculated over the ten growing seasons plotted in Figs 5 and 6. The selection ratio represents the factor by which the frequency of the resistant strain is multiplied over one growing season. The selection ratios were calculated per growing season over the ten growing seasons and then the median of the resultant distribution was calculated. Median selection ratios were calculated at 4.37 and 4.0 for Figs 6 and 7 respectively (S3 Table), which suggested a slightly larger rate of increase of the fungicide-resistant strain when a solo T1 spray was applied. However, this trend was reversed when the fungicide half-life was increased (*l*_*long*_ rather than *l*_*short*_), leading to median selection ratios for a solo T1 spray and a seed treatment of 3.61 and 3.86, respectively.

### Effective Fungicide Lives

Effective lives were compared between four treatment programmes over a range of doses. The regimens were labelled in results Tables 2–4 as the following:

1. ST + T1: A seed treatment and a foliar spray at T1 were applied each growing season.
2. ST + T2: A seed treatment and a foliar spray at T2 were applied each growing season.
3. T1 + T2: Two foliar sprays at T1 and T2 were applied each growing season.
4. ST + T1 + T2: A seed treatment and two foliar sprays at T1 and T2 were applied every growing season. In this regimen the doses of the foliar treatments at T1 and T2 were equal.

**Table 2:** Effective lives in the presence of low and high fungicide breakdown rates.

ST dose	foliar dose (per treatment)	Low breakdown			High breakdown		
		ST+T1	ST+T2	ST+T1+T2	ST+T1	ST+T2	ST+T1+T2
0	0	-	-	-	-	-	-
0	0.2	-	-	-	-	-	-
0	0.4	-	-	5	-	-	-
0	0.6	-	-	4	-	-	5
0	0.8	-	-	4	-	-	5
0	1	-	-	4	-	-	5
0.6	0	-	-	-	-	-	-
0.6	0.2	4	5	4	-	-	4
0.6	0.4	4	5	4	4	5	4
0.6	0.6	4	4	3	4	5	4
0.6	0.8	4	4	3	4	5	4
0.6	1	4	4	3	4	5	3
1	0	-	-	-	-	-	-
1	0.2	4	5	4	4	4	4
1	0.4	4	4	3	4	5	4
1	0.6	4	4	3	4	5	4
1	0.8	4	4	3	4	4	3
1	1	4	4	3	4	4	3

Effective lives (in years) in the presence of high and low fungicide breakdown rates. All effective lives were calculated for the scenario of a constant seed treatment uptake rate model, and the seed treatment being parameterised such that it provides the same level of control as a T1 spray when applied at dose 1. ST refers to a seed treatment and T1 and T2 refer to foliar treatments at the full emergence of eventual leaf 3 and the flag leaf, respectively. The table has been truncated for brevity. Full tables, also containing results for dose 0.2, 0.4 and 0.8, are in S4 and S5 Tables. Dashes indicate simulations for which effective disease control was not achieved during the first growing season.

**Table 3:** Effective lives for two seed treatment efficacy parameterizations.

ST_dose	foliar_dose (per treatment)	ST = T1			ST = 40% of AUDPC		
		ST_T1	ST_T2	ST_T1_T2	ST_T1	ST_T2	ST_T1_T2
0	0	-	-	-	-	-	-
0	0.2	-	-	-	-	-	-
0	0.4	-	-	5	-	-	5
0	0.6	-	-	4	-	-	4
0	0.8	-	-	4	-	-	4
0	1	-	-	4	-	-	4
0.6	0	-	-	-	-	-	-
0.6	0.2	4	-	4	-	-	5
0.6	0.4	4	5	4	-	-	4
0.6	0.6	4	4	3	4	5	4
0.6	0.8	4	4	3	4	5	4
0.6	1	4	4	3	4	5	4
1	0	-	-	-	-	-	-
1	0.2	4	5	4	-	-	4
1	0.4	4	4	3	4	5	4
1	0.6	4	4	3	4	5	4
1	0.8	4	4	3	4	4	3
1	1	4	4	3	4	4	3

Effective lives (in years) for two different seed treatment (ST) efficacy parameterizations: 1) ST = T1, whereby the seed treatment provides the same level of control as a T1 spray and 2) ST = 40% of AUDPC, whereby the seed treatment provides a 60% reduction in AUDPC when applied at dose 1. All simulations were run for the scenario of a low fungicide breakdown rate and the transpiration-based seed treatment uptake model. The table has been truncated for brevity. Full tables, also containing results for dose 0.2, 0.4 and 0.8, are in S9 and S11 Tables. Dashes indicate simulations for which effective disease control was not achieved during the first growing season.

**Table 4:** Effective lives for the constant versus transpiration-based seed treatment uptake model.

ST_dose	foliar_dose	Constant uptake			Transpiration-based uptake		
		ST_T1	ST_T2	ST_T1_T2	ST_T1	ST_T2	ST_T1_T2
0	0	-	-	-	-	-	-
0	0.2	-	-	-	-	-	-
0	0.4	-	-	-	-	-	-
0	0.6	-	-	5	-	-	5
0	0.8	-	-	5	-	-	5
0	1	-	-	5	-	-	5
0.6	0	-	-	-	-	-	-
0.6	0.2	-	-	4	-	-	5
0.6	0.4	4	5	4	4	4	4
0.6	0.6	4	5	4	4	5	4
0.6	0.8	4	5	4	4	5	4
0.6	1	4	5	3	4	5	4
1	0	-	-	-	-	-	-
1	0.2	4	4	4	4	5	4
1	0.4	4	5	4	4	5	4
1	0.6	4	5	4	4	4	4
1	0.8	4	4	3	4	4	3
1	1	4	4	3	4	4	3

Effective lives (in years) for two different seed treatment uptake models: constant and transpiration-based. All simulations were run for the scenario of a high fungicide breakdown rate and a seed treatment (ST) which at dose 1 provides the same level of control as a T1 spray. The tables have been truncated for brevity. Full tables, also containing results for dose 0.2, 0.4 and 0.8, are in S5 and S8 Tables. Dashes indicate simulations for which effective disease control was not achieved during the first growing season.

#### High and low fungicide breakdown rates

Effective lives for both a high and a low fungicide breakdown rate are shown in Table 2. The maximum seed treatment dose was set to provide an equivalent HAD gain to a T1 spray, and the constant uptake model was used. Note that the column ST+T1+T2 is the T1+T2 spray program for entries of the table where the seed treatment dose is zero.

The table leads to three key conclusions for this set of simulations. Firstly, the ST+T1+T2 column shows that adding a seed treatment to a two foliar spray program shortens the effective life of the fungicide. This holds for both the low and the high fungicide breakdown rate. Secondly, the effective life of all spray programs that include a seed treatment is equal to or smaller than a spray program with two foliar sprays. Again this holds for low and high fungicide breakdown rates. Thirdly, comparing treatment programs with equal total fungicide dose used, the effective life of the spray program including a seed treatment is equal to or smaller than that of a program without a seed treatment. See also S4 and S5 Tables for this comparison in more detail.

Clearly these qualitative conclusions are not affected by the fungicide decay rate showing that the conclusions are not sensitive to the decay rate parameter. In the following paragraphs we will not vary this parameter, but we have checked that the conclusions are not sensitive to the rate of fungicide decay.

#### Maximum seed treatment dose value

The effective lives for the two different seed treatment efficacy parameterizations in the presence of a low fungicide breakdown rate and the transpiration-based uptake model are given in Table 3. In the columns marked ‘ST = T1’ the seed treatment provides a HAD gain equal to that of a T1 spray. In the columns marked ‘ST = 40% of AUDPC’ the maximum seed treatment dose reduced the AUDPC by 60%. From Table 3 we can draw the same three qualitative conclusions as we reached from Table 2, (i) adding a seed treatment to a spray program with two foliar sprays reduces the effective life of the fungicide, (ii) the effective life of all spray programs that includes a seed treatment is equal to or smaller than a spray program of only two foliar applications, and (iii) comparing spray programs with equal total dose the effective life of the program including a seed treatment is equal to or smaller than that of the program without a seed treatment. These conclusions hold irrespective of the chosen efficacy of the seed treatment (ST = T1 or ST = 40% of AUDPC).

#### Seed treatment uptake model

Finally, in Table 4 we compare the effective life for the constant fungicide uptake model with those for the transpirational uptake model. Again the same set of three key conclusions relating to the effective lives obtained by the different fungicide treatment programs were present. These conclusions were therefore also not affected by the choice of fungicide uptake dynamics from the seed coating.

This study showed that irrespective of the choice in the uptake dynamics of the fungicide from the seed coating, the efficacy of the seed treatment and the fungicide breakdown rate used, the key qualitative conclusions regarding the effective lives remained the same. Tables 2, 3 and 4 are only a subset of all possible permutations of uptake model, breakdown rate and seed treatment efficacy. However, we have calculated all possible combinations of these three aspects and present the results in S4–S11 Tables. The reader can verify from this supplementary material that the conclusions hold for any combination of the three factors.

#### Ascospore longevity and length of period for HAD calculation

There is little published information describing over what time period, or from what parts of the crop canopy, ascospores, which cause infection of crops in the following season, are produced. To test if the results were sensitive to this, in S12 Table, the effect of increasing the longevity of the simulated ascospores that remained in pseudothecia on crop debris left over from the previous season (and are therefore carried forward as the founder population for the following season) was extended by summing over all 11 leaves to calculate the proportion of fungicide-resistant individuals in the population (see Eq 6), and effective lives were calculated over the dose range. The aforementioned trends in the effective lives remained, therefore the model output did not seem sensitive to these changes.

## Discussion

We developed a model to simulate epidemics of *Zymoseptoria tritici* on winter wheat controlled by applying a systemic fungicide to seed and foliage. We used the model to compare the selection pressures for fungicide resistance between both fungicide treatment types. Unlike foliar sprays, for which information on dose response curves are readily available, there is little information on the efficacy and the uptake dynamics of systemic seed treatments [27–30]. We therefore did a sensitivity analysis with respect to the parameter values for the systemic seed treatment model and combined them with a structural sensitivity analysis, using two different approaches to model the uptake dynamics of the fungicide from the seed coating. Our results show that the qualitative trends in the model output are insensitive to: (i) the value of the parameter scaling the seed treatment efficacy, (ii) the half life time of the fungicide, and (iii) the model description of the fungicide uptake by the plant from the seed coating. As further mentioned a range of other parameters were explored by sensitivity analysis and also showed that the conclusions were very robust to these parameter changes.

The model outputs result in three key conclusions about the effect of seed treatment on fungicide resistance development as compared to foliar sprays: (i) Adding a seed treatment to a spray program with two foliar sprays reduces the effective life of the fungicide. (ii) The effective life of all spray programs that include a seed treatment is equal to or shorter than a spray program of only two foliar applications. (iii) Comparing spray programs with equal total dose the effective life of the program including a seed treatment is equal to or smaller than that of the program without a seed treatment.

For *Zymoseptoria tritici* on wheat, and for SDHI type solo fungicides, the selection for fungicide resistance of a seed treatment is equal or at least comparable to that of a foliar spray, if the seed treatment efficacy is equal or comparable to that of a foliar spray. This finding is consistent with the current FRAC guideline concerning seed treatments of SDHI fungicides [31].

Our model predictions suggest that for epidemics of *Z*. *tritici* on winter wheat being treated by SDHI fungicides, there is no gain in the effective life that can be obtained from seed treatments compared to foliar treatments as the maximum effective life was consistently attained by two foliar sprays. However, we recognize that the effective fungicide life is only one aspect of the usefulness of a fungicidal seed treatment, and that systemic seed treatments may still be useful for controlling disease.

The current FRAC guidelines recommended at most two SDHI treatments unless the following ‘risk modifiers’ were in place: i) if the epidemic is being propagated by a low-risk foliar pathogen or a seed or soil-borne pathogen, ii) if the SDHI is mixed with a different mode of action that is able to solely provide control, and iii) if the following foliar spray does not contain a SDHI [31]. The first of the modifiers is irrelevant for foliar-based epidemics of *Zymoseptoria tritici*. However, the second and third modifiers correspond to fungicide mixture and alternation strategies, and as suggested by FRAC may allow SDHI seed treatments to be combined with more than one foliar spray without significantly reducing the effective life.

## Supporting Information

S1 File Model parameter derivation. (DOCX)

S2 File Seed treatment program code.–C++ code used within Visual Studio 2015. (DOCX)

S1 Table Estimated initial seed treatment dose (ST) resulting in % HAD losses (in year 1) similar to those achieved with a T1 foliar spray for a range of model scenarios. (DOCX)

S2 Table Estimated initial seed treatment dose (ST) leading to a 60% reduction in AUDPC for a range of model scenarios. (DOCX)

S3 Table Median selection ratios under three treatment regimes. (DOCX)

S4 Table Effective lives (in years) for a seed treatment that provides the same level of control as a T1 foliar treatment in the presence of the constant seed treatment uptake model and a low fungicide breakdown rate. (DOCX)

S5 Table Effective lives (in years) for a seed treatment that provides the same level of control as a T1 foliar treatmentin the presence of the constant seed treatment uptake model and a high fungicide breakdown rate. (DOCX)

S6 Table Effective lives (in years) for a seed treatment leading to a 60% reduction in AUDPC in the presence of a constant seed treatment uptake rate and a high fungicide breakdown rate. (DOCX)

S7 Table Effective lives (in years) for a seed treatment leading to a 60% reduction in AUDPC in the presence of a constant seed treatment uptake rate and a low fungicide breakdown rate. (DOCX)

S8 Table Effective lives (in years) for a seed treatment that provides the same level of control as a T1 foliar treatment in the presence of the transpiration-based seed treatment uptake model and a high fungicide breakdown rate. (DOCX)

S9 Table Effective lives (in years) for a seed treatment that provide the same level of control as a T1 foliar treatment in the presence of the transpiration-based seed treatment uptake model and a low fungicide breakdown rate. (DOCX)

S10 Table Effective lives (in years) for a seed treatment leading to a 60% reduction in AUDPC in the presence of the transpiration-based seed treatment uptake model and a high fungicide breakdown rate. (DOCX)

S11 Table Effective lives (in years) for a seed treatment leading to a 60% reduction in AUDPC in the presence of the transpiration-based seed treatment uptake model and a low fungicide breakdown rate. (DOCX)

S12 Table Effect of different ascospore initiation. (DOCX)
