# Supplementary material for: The Evolution of Fungicide Resistance Resulting from Combinations of Foliar-Acting Systemic Seed Treatments and Foliar-Applied Fungicides: A Modeling Analysis
Source: PLoS One. 2016 Aug 29;11(8):e0161887. doi: 10.1371/journal.pone.0161887 (PMC5003396; doi:10.1371/journal.pone.0161887)
Supplement: S1 File — (DOCX) [file pone.0161887.s001.docx]

**S1 File. Model parameter derivation**

The model parameters derived in this appendix represent wheat plants and *Z. tritici* epidemics under UK field conditions. All dynamics are modelled in degree days, whereby daily measurements are converted to degree day measurements by applying a base temperature of 0 ⁰C an average temperature of 15.2 ⁰C (Met Office, United Kingdom). The decimal code system for measuring wheat development using the prefix GS, denoting the growth stage used in this article, is described by Chang et al. [1].

**Crop dynamics.** Wheat leaves emerge a phyllochron (*P*) apart, whereby *P* equals 122 degree days [2]. The time of full emergence of the flag leaf, $t_{emergence\_1}$, occurs 3*P* before the onset of flowering (GS61) at 2066 degree days [3, 4], leading to $t_{emergence\_1}=1700$. The calculation of the flag leaf initiation time, $t_{initiation\_1}$, uses the approach described by Milne, Paveley (5), such that , $t_{initiation\_1}=t_{emergence\_1}-2ln(3)/g$. Whereby, *g* is the constant growth rate of 0.034 degree days^-1^, yielding $t_{initiation\_1}=1635$ degree days. From this information, the initiation and emergence times of the other leaves can then be derived.

Milne, Paveley (5) provide maximum leaf area estimates for leaf layers 1 to 5 based on experimental data and estimates for the rosette leaves (leaf layers 6 to 11) simulated according to the England (6) simulation. According to van den Berg, Paveley (7), the top five leaf layers have a combined area index of 4.2 at anthesis. Given a maximum total area index of 5.9, this leaves a total area index of 1.7 for the rosette leaves. The leaf layer specific maximum AIs, $A_{max\_i}$, are subsequently calculated according to the leaf’s average actual size [5], resulting in AIs ranging between 0.23 and 1.05.

Lawless, Semenov (8) defined leaf size dependent lag periods, specifying the time between full leaf emergence and the onset of senescence, with a maximum lag period of 9*P* for leaf 2 (largest leaf) and a minimum lag period of 4*P* for leaves 10 and 11. The onset of senescence of leaf 2, $t_{senes\_2}$, is then given by the time of full emergence, $t_{emergence\_2}=1513$, plus the lag period, $T_{lag\_2}=1098$, giving $t_{senes\_2}=2676$ degree days. The lag periods and, hence, the onset of senescence of the other leaves can be calculated according to their maximum AI.

Senescence occurs with a mean rate of $s=0.05$ [5]. Using the time of death of leaf 2 at 2900 degree days (GS87) as a reference point resulting in a total senescence period of 224 degree days, the time of death of the other leaves, $t_{death\_i}$ , can be approximated by

$t_{death\_i}$=$\left( \frac{224A_{max\_i}}{A_{max\_2}} \right)+t_{senes\_i}$.

The top four leaf layers are subject to stem extension at a rate of 1 cm per 10 degree days up to a maximum extension of 10 cm per internode [7, 9].

**Disease dynamics.** Ascospore influx is assumed to peak during the life-time of the rosette leaves, whilst subsequently declining from the time of death of leaf layer 5, leading to $\lambda=0.0035$ and $\eta=1$.

The average length of the infectious period, 1/*μ*, was taken to be 456 degree days (30 days) based on experiments by Eyal (10), leading to a value for *μ* at 1/456 = 0.002. Following Lovell, Hunter (11) who reported an average latent period, 1/*δ*, of approximately 2 phyllochrons or 244 degree days, our value for *δ* is 1/244 = 0.004. For natural rainfall events, the rate of splash coverage decline with plant height was found to be -0.1 per unit of distance [12], leading to $\sigma_{up}=0.1$. van den Berg, Paveley (7) estimated $\sigma_{\mathrm{down}}$ to be 0.01. The number of simulated latent compartments, *m*, was set to 10, resulting in a smooth and biologically realistic distribution of the latent period [7, 13].

Finally, the ascospore and pycnidiospore transmission rates were estimated by visually fitting the disease progress curves on the top four leaf layers to a large set of disease severity observations [14]. This data set contained leaf layer specific disease severity observations for 10 winter wheat cultivars, measured across 7 sites and over 4 years. Disease progress curves with a maximum disease severity $\leq$ 5% were excluded from the analysis and all remaining progress curves were standardized in time by selecting GS32 as a common reference time point. Average disease severities were subsequently binned into intervals of one hundred degree days. During the fitting process the ascospores transmission rate, $\gamma$, and the pycnidiospore transmission rate, $\rho$, were varied until the leaf specific disease progress curves as predicted by the model simulations tracked through the largest number of data points (within their standard error range), leading to estimates of $\gamma=4\cdot{10}^{-10}$ and $\rho=0.007$.

**Fungicide dynamics.** The fungicide parameters are based on a generic SDHI fungicide. For the foliar treatments the maximum dose allowed per application is determined by averaging over three SDHI active ingredients: fluxapyroxad, penthiopyrad and isopyrazam. The maximum dose of fluxapyroxad and penthiopyrad were obtained from the labels of products Imtrex® and Vertisan® and are 12.5 mg m^-2^ [15] and 35 mg m^-2^ [16], respectively. The maximum dose for solo use of isopyrazam was reported by Bounds, Blake (17). The generic SDHI fungicide used in this study hence has a maximum dose of 20 mg m^-2^ when applied as a foliar treatment and this dose is henceforth referred to as the foliar treatment label dose. For wheat Hosoi, Nakai (18) report a leaf angle of approximately 50 degrees. The average fractional projection area onto a horizontal surface can then be calculated to be $\tau=\cos\left( 40 \right)=0.77$. A wheat leaf is assumed to have a thickness of $q=0.001$ meter. No information is available regarding systemic SDHI half-lives. The half-life of the systemic quinone outside inhibitor (QOI) pyraclostrobin has however been estimated to range between 3 and 11 days [19-21]. In this study the model results are derived for two fungicide half-lives that fall within this range, i.e. 5 and 10 days, leading to a low and high fungicide breakdown rate of $\nu_{low}=0.0046$ and $\nu_{high}=0.009$ , respectively.

Seed treatment dosages are normally given in the units of mg seed^-1^. However, under the assumption of an average sowing density of 300 plants per m^2^, the dose can be converted to mg m^-2^ ground area, which means that the seed treatment dose can be directly compared to the foliar treatment dose. The simulations compare two initial seed treatment dosages, representing a high and a low seed treatment efficacy.

Under the assumption that a systemic seed treatment is unlikely to provide a stronger reduction in disease severity than a T1 foliar spray, for the high seed treatment efficacy the initial seed treatment dose was chosen such that a seed treatment provides the same antifungal effect as a T1 foliar spray. Hereto, $N_{seed}$ was adjusted until the % HAD loss during a single growing season when using a seed treatment approximated the % HAD loss when using a T1 foliar spray. The estimated doses varied according to the fungicide breakdown rate and the seed treatment uptake model used. Table S1 provides an overview of the initial dose values and resultant % HAD losses for each model scenario.

For the low treatment efficacy, data on disease control achieved in the presence of a seed treatment in comparison with untreated plots were used. Parker and Lovell (22) report spore washing data (spores ml^-1^) for winter wheat leaves infected with *Zymoseptoria tritici* comparing leaves from untreated plots with those from plots treated with a fluquinconazole (product Jockey) seed treatment. The majority of the data could be represented by a 500 to 1200 degree day sampling window, whereas data derived over a shorter time period were linearly extrapolated over the full time window using R package *Hmisc*. Subsequently, the areas under the disease progress curves (AUDPCs) were calculated by numerically integrating the spore washing values using the method described in Gill and Miller (23). From these integrations it could be derived that on average a seed treatment resulted in a 60% reduction in AUDPC. Values of *N_seed_* were then adjusted until the model obtained a 60% reduction in AUDPC. Table S2 provides an overview of the initial dose values and the resultant reduction in AUDPC for the different model scenarios.

Two seed treatment uptake models, describing the movement of the fungicide into the leaf layers were compared. The first model assumes a constant fungicide uptake rate, β. In this model the uptake rate was chosen such that 99.99% of the fungicide has been depleted from the seed at the time that leaf 5 is fully dead (i.e. t=2094 degree days), representing the death of the rosette. This led to β=0.0055. The second model, hereafter referred to as the transpiration-based uptake model, assumes a time dependent fungicide uptake rate, $\beta(t)$. Parameters *a*, *b* and *c* associated with this function were estimated by fitting a sigmoidal function (eqn. 18) to winter wheat water use data in L bin^-1^ as measured by Ober, Werner (24), leading to *a=0.003*, *b=0.002*, and *c=0.0015*. The asymptote, *y*, was chosen such that 99.99% of the fungicide has been depleted from the seed at the time that leaf 5 is fully dead, leading to *y=0.027*.

For the generic SDHI used in the model simulations the dose-response curves were based on three active ingredients (fluxapyroxad, penthiopyrad and isopyrazam). Anonymous (25) and Bounds, Blake (17) report percentage declines in Septoria severity as a result of different doses of penthiopyrad and isopyrazam applied at the full emergence of leaf 2 (estimated to be at 1578 degree days), respectively. Disease severity observations were made on leaves 3 and 4 three weeks after spraying and on leaves 1 and 2 six weeks after spraying. The average of these observed disease severities was used as an estimate of the disease severity experienced by the upper leaves. The dose-response parameters were then adjusted until the model’s predicted average decrease in severity with an increased dose closely matched the field observations. Note that, in the model, the disease severities were also assessed on leaves 3 and 4 (or 1 and 2) 3 weeks (or 6 weeks) after a single spray at GS32. This led to the approximation *α_δ_high_* = *α_ε_high_* = *α_ρ_high_* =0.5 and *k_δ_high_* = *k_ε_high_* = *k_ρ_high_*=0.003 for an SDHI with a high breakdown rate and *α_δ_low_* = *α_ε_low_* = *α_ρ_low_* =0.45 and *k_δ_low_* = *k_ε_low_* = *k_ρ_low_*=0.0025 for an SDHI with a low breakdown rate.

1. Chang TT, Konzak CF, Zadoks JC. A decimal code for the growth stages of cereals. Weed Res. 1974;14:415-21.

2. Anonymous. The wheat growth guide. Home-Grown Cereals Authority, United Kingdom., 2008.

3. Brooking IR, Jamieson PD, Porter JR. The influence of daylength on final leaf number in spring wheat. Field Crops Res. 1995;41:155-65.

4. Jamieson PD, Semenov MA, Brooking IR, Francis GS. *Sirius*: a mechanistic model of wheat response to environmental variation. Eur J Agron. 1998;8:161-79.

5. Milne A, Paveley ND, Audsley E, Livermore P. A wheat canopy model for use in disease management decision support systems. Ann Appl Biol. 2003;143:265-74.

6. England RA. Modelling the performance of spray decision rules for cereal crops: 1. The Host Crop. Wrest Park, Silsoe, Bedford: NIAE, 1985.

7. van den Berg F, Paveley ND, van den Bosch F. Optimal fungicide application timings for disease control are also an effective anti-resistance strategy: a case study for *Mycosphaerella graminicola* on wheat. Phytopathology. 2013;103:1209-19.

8. Lawless C, Semenov MA, Jamieson PD. A wheat canopy model linking leaf area and phenology. Eur J Agron. 2005;22:19-32.

9. Audsley E, Milne A, Paveley ND. A foliar disease model for use in wheat disease management decision support systems. Ann Appl Biol. 2005;147:161-72.

10. Eyal Z. Kinetics of pycnospore liberation in *Septoria tritici*. Can J Bot. 1971;49:1095-9.

11. Lovell DJ, Hunter T, Powers SJ, Parker SR, van den Bosch F. Effect of temperature on latent period of septoria leaf blotch on winter wheat under outdoor conditions. Plant Pathol. 2004;53:170-81.

12. Shaw MW. Assessment of upward movement of splash using a fluorescent tracer method and its application to the epidemiology of cereal pathogens. Plant Pathol. 1987;36:201-13.

13. Cunniffe NJ, Stutt ROJH, van den Bosch F, Gilligan CA. Time-dependent infectivity and flexible latent and infectious periods in compartmental models of plant disease. Phytopathology. 2012;102:365-80.

14. te Beest DE, Shaw MW, Pietravalle S, van den Bosch F. A predictive model for early-warning of Septoria leaf blotch on winter wheat. Eur J Plant Pathol. 2009;124:413-25.

15. BASF. Imtrex^(R)^ MAPP 15509 2014 [cited 2015 08 July]. Available from: <http://www.agricentre.basf.co.uk/agroportal/uk/media/product_files_uk/labels/Imtrex.pdf>.

16. Dupont. Vertisan® MAPP 16104 2014 [cited 2015 08 July]. Available from: <http://www.dupont.co.uk/content/dam/assets/industries/agriculture/assets/Vertisan%20UK%20NEW%20CLP%20version%2029.9.14.pdf>.

17. Bounds P, Blake J, Fraaije BA, Parsons D, Knight S, Burnett FJ, et al. Fungicide performance on winter wheat. London, UK: HGCA, 2012 Contract No.: HGCA PROJECT REPORT 488

18. Hosoi F, Nakai Y, Omasa K. Estimating the leaf inclination angle distribution of the wheat canopy using a portable scanning lidar. J Agric Meteorol. 2009;65:297-302.

19. Garau VL, de Melo Abreu S, Caboni P, Angioni A, Alves A, Cabras P. Residue-free wines: fate of some quinone outside inhibitor (QoI) fungicides in the winemaking process. J Agric Food Chem. 2009;57:2329-33.

20. Hanafi A, Garau VL, Caboni P, Sarais G, Cabras P. Minor crops for export: a case study of boscalid, pyraclostrobin, lufenuron and lambda-cyhalothrin residue levels on green beans and spring onions in Egypt. J Environ Sci Health Part B. 2010;45:493-500.

21. Zhang XY, Loyce C, Meynard JM, Monod H. Modelling the effect of cultivar resistance on yield losses of winter wheat in natural multiple disease conditions. Eur J Agron. 2007;26:384-93.

22. Parker SR, Lovell DJ. Quantifying the benefits of seed treatment for foliar disease control. In: Biddle AJ, editor. Seed Treatment: Challenges & Opportunities, Proceedings. British Crop Protection Council Symposium Proceedings. Farnham: British Crop Protection Council; 2001. p. 181-8.

23. Gill PE, Miller GF. An Algorithm for the Integration of Unequally Spaced Data. Compu J. 1972;15(1):80-3. doi: 10.1093/comjnl/15.1.80.

24. Ober ES, Werner P, Flatman E, Angus B, Jack P, Tapsell C. Improving water use efficiency and drought tolerance in UK winter wheats. London, UK: HGCA, 2013 Contract No.: HGCA PROJECT REPORT 476

25. Anonymous. Fungicide performance in wheat 2014. 2014.
