# Supplementary material for: The Evolution of Fungicide Resistance Resulting from Combinations of Foliar-Acting Systemic Seed Treatments and Foliar-Applied Fungicides: A Modeling Analysis
Source: PLoS One. 2016 Aug 29;11(8):e0161887. doi: 10.1371/journal.pone.0161887 (PMC5003396; doi:10.1371/journal.pone.0161887)
Supplement: S2 File — (DOCX) [file pone.0161887.s002.docx]

**S2 File. Seed treatment program code – C++ code used within Visual Studio 2015**

This file contains all code required to replicate the simulation described within the main manuscript. They files are either in text format (.txt); source code (.cpp) or header file code (.h). To run the files within the Visual Studio 2015 environment please copy the text found under each section in a text fiel, source file or header file as appropriate and with the same title as section heading.

**epidemic_st.cpp**

#include "epidemic_st.h"

#include "JKUtils.h"

#include "parameters.h"

using namespace std;

double current_conid = 0.0; //variables for printing our ascospore and conidia

double current_asc = 0.0; //contribution in our differential equations

/**

* Constructor. Initialises our variable for the number of latent

* compartments we are simulating and initialises our arrays for

* calculation of our ODE's.

*

* @param no_of_latent_compartments - number of latent

* compartments we are simulating

*

* @param layers - list of leaf layer objects/ needed

* for initialising our variable/temp variable

* /derivative hashes for each leaf layer

* @param fungicideIn - reference to fungicide object passed in

* from main. Don't forget about the intitialisation

* list!

*

*/

epidemic_st::epidemic_st( std::list<leafLayer*>* layers,

fungicide_st & h_fungicideIn, fungicide_st & l_fungicideIn )

: high_fung( h_fungicideIn ), low_fung( l_fungicideIn ){

high_fung = h_fungicideIn;

low_fung = l_fungicideIn;

//in the first year the proportion of resistant strain is theta zero

prop_resistant = parameters::theta_zero;

cout<<"initial prop_resistant"<<prop_resistant<<"\n";

//set up our map of variable and derivative arrays per leaf layer

list<leafLayer*>::iterator it;

for( it = layers->begin(); it != layers->end(); it ++ ){

leafLayer * currentLayer = (*it);

vector<long double> * layer_variable_array = new vector<long double>();

vector<long double> * layer_temp_variable_array = new vector<long double>();

vector<long double> * layer_deriv_array = new vector<long double>();

layer_variable_map.insert( make_pair( currentLayer, layer_variable_array ) );

layer_temp_variable_map.insert( make_pair( currentLayer, layer_temp_variable_array ) );

layer_derivative_map.insert( make_pair( currentLayer, layer_deriv_array ) );

}//end for

cout<<"initialising k vectors\n";

initialise_k_vectors( layers->size() );

parameters::print_bookkeeping_variables();

}//end constructor

/**

* calculates our number of wind-blown ascospores as

* a bell-shaped function of time.

*

* @param t - time measurement

* @return - dependent variable representing our

* density of ascospores entering the

* system

*/

double epidemic_st::calc_X_t( double t ){

double X_t = parameters::eta * pow( t, 2 ) * exp( -parameters::lambda * t ) ;

return X_t;

}

/**

* loops through all of our leaf layers and sums up our infectious densities.

* then takes the proportion of resistant infectious densities compared with

* sensitive density.

*

* @param layers - list of leaf layer objects

* @param res_prop - file stream to write proportion of resistant to

*/

void epidemic_st::calc_prop_of_resistant( list<leafLayer*>* layers, fstream & res_prop ){

//first sum up sensitive and resistant latent areas

double sum_sensitive = 0.0;

double sum_resistant = 0.0;

list<leafLayer*>::iterator it;

for( it = layers->begin(); it != layers->end(); it ++ ){

leafLayer * layer = (*it);

sum_sensitive += layer->getSensitiveInfectiousArea();

sum_resistant += layer->getResistantInfectiousArea();

}//end for

/**

* now calculate resistant proportion. If either the numerator or denominator are

* zero, we leave prop_resistant at zero.

*/

if( sum_resistant == 0 || ( sum_resistant + sum_sensitive ) == 0 ){

this->prop_resistant = 0.0;

}else{

this->prop_resistant = sum_resistant / ( sum_resistant + sum_sensitive );

}//end else

res_prop<<"prop of resistant = "<<prop_resistant<<"\n";

}//end method

/**

* loops through all of our leaf layers and sums up our infectious densities.

* then takes the proportion of resistant infectious densities compared with

* sensitive density.

*

* @param layers - list of leaf layer objects

* @param res_prop - file stream to write proportion of resistant to

*/

void epidemic_st::calc_prop_of_resistant( double & sum_sensitive, double & sum_resistant, fstream & res_prop ){

/**

* We calculate the resistant proportion. If either the numerator or denominator are

* zero, we leave prop_resistant at zero.

*/

if( sum_resistant == 0 || ( sum_resistant + sum_sensitive ) == 0 ){

this->prop_resistant = 0.0;

}else{

this->prop_resistant = sum_resistant / ( sum_resistant + sum_sensitive );

}//end else

res_prop<<"prop of resistant = "<<prop_resistant<<"\n";

sum_sensitive = 0.0;

sum_resistant = 0.0;

}//end method

/**

* initialises our derivative and variable arrays for a particular leaf layer.

* assumed to be called from within a loop iterating through our leaf layers.

*

* @param layer_i - leaf layer of interest

*/

void epidemic_st::initialise_calculation_arrays( leafLayer * layer_i ){

//get our arrays from our map

vector<long double> * derivative_array = layer_derivative_map.at( layer_i );

vector<long double> * variable_array = layer_variable_map.at( layer_i );

vector<long double> * temp_variable_array = layer_temp_variable_map.at( layer_i ); //contains a copy of all values in

//the variable array

//set up our our derivatives array:

derivative_array->clear();

derivative_array->push_back( 0.0 ); //for A_i

derivative_array->push_back( 0.0 ); //for H_i

derivative_array->push_back( 0.0 ); //for L1_S_i

derivative_array->push_back( 0.0 ); //for L1_R_i

derivative_array->push_back( 0.0 ); //for I_S_i

derivative_array->push_back( 0.0 ); //for I_R_i

/**

* add all of our sensitive latent compartments - excluding the first (no_of_latent_compartments - 1)

* as this has already been stored above.

*/

for( unsigned int i = 0; i < parameters::no_of_latent_compartments - 1 ; i ++ ){

derivative_array->push_back( 0.0 );

}

/**

* get all of our resistant latent compartments - excluding the first (no_of_latent_compartments - 1)

* as this has already been stored above.

*/

for( unsigned int i = 0; i < parameters::no_of_latent_compartments - 1 ; i ++ ){

derivative_array->push_back( 0.0 );

}

/**

* add two more elements for Nseed and N_layer_i. YES - the same value for the derivative will be

* stored for Nseed in every leaf layer. But I am forced to do this currently.

*/

derivative_array->push_back( 0.0 ); //for high risk NSeed

derivative_array->push_back( 0.0 ); //for high risk N_layer_i

derivative_array->push_back( 0.0 ); //for low risk NSeed

derivative_array->push_back( 0.0 ); //for low risk N_layer_i

//now we set up our variables array to contain our initial conditions:

variable_array->clear();

variable_array->push_back( layer_i->getArea() ); //A_i

variable_array->push_back( layer_i->get_healthy_area() ); //H_i

variable_array->push_back( layer_i->getSensitiveLatentCompartments()->at( 0 ) ); //L1_S_i

variable_array->push_back( layer_i->getResistantLatentCompartments()->at( 0 ) ); //L1_R_i

variable_array->push_back( layer_i->getSensitiveInfectiousArea() ); //I_S_i

variable_array->push_back( layer_i->getResistantInfectiousArea() ); //I_R_i

//set up our latent compartments. Note we are skipping the first special compartment

vector<double> * layer_i_latent_compts_S = layer_i->getSensitiveLatentCompartments();

vector<double> * layer_i_latent_compts_R = layer_i->getResistantLatentCompartments();

/**

* now to our variable array we add all of our sensitive and resistant latent compartments

* - excluding the first (no_of_latent_compartments - 1), this has been added above.

*/

for( unsigned int i = 1; //skipping first element

i < layer_i_latent_compts_S->size(); i ++ ){

variable_array->push_back( layer_i_latent_compts_S->at( i ) );

}//end for

for( unsigned int i = 1; //skipping first element

i < layer_i_latent_compts_R->size(); i ++ ){

variable_array->push_back( layer_i_latent_compts_R->at( i ) );

}//end for

//two more for Nseed and Nlayeri

variable_array->push_back( high_fung.get_Nseed() ); //for high risk NSeed

variable_array->push_back( layer_i->get_high_risk_seed_treat_amount() ); //for high risk N_layer_i

variable_array->push_back( low_fung.get_Nseed() ); //for low risk NSeed

variable_array->push_back( layer_i->get_low_risk_seed_treat_amount() ); //for low risk N_layer_i

//now we copy all the above variables into our variable array

copyVectors( variable_array, temp_variable_array );

//initialise our k vectors

reset_k_vectors();

}//end function

/**

* function calls for all of our differential equations.

*

* @param layer_i - leaf layer of interest

* @param layers - list of all layer objects

* @param t - time value

*

*/

void epidemic_st::derivs( leafLayer * layer_i, list<leafLayer*>* layers, double t ){

//calculate our explicit foliar derivatives:

calc_healthy_area( layer_i, layers, t );

calc_first_latent_class( layer_i, layers, t );

calc_latent_classes( layer_i, t, layers );

calc_infected_area( layer_i, t, layers );

//high risk seed treatment fungicide derivatives

high_fung.ST_seed_deriv( layer_i, layer_temp_variable_map, layer_derivative_map, t );

high_fung.ST_layer_deriv( layer_i, layers, t, layer_temp_variable_map, layer_derivative_map );

//low risk seed treatment fungicide derivatives

low_fung.ST_seed_deriv( layer_i, layer_temp_variable_map, layer_derivative_map, t );

low_fung.ST_layer_deriv( layer_i, layers, t, layer_temp_variable_map, layer_derivative_map );

}

/**

* Function used to initialise our variable and derivative array per leaf layer

* call our functions for calculating our derviatives and call our integrator.

*

* sets up our derivatives and variables arrays for variables healthy area (H_i,

* latent compartment 1 for sensitive and resistant strains (L1_S_i, L1_R_i) and

* sensitive and resistant infectious densities (I_S_i and I_R_i).

*

* @param step_size - the step size used for integrating

* @param layers - array of leaf layers for calculating our variables

* @param t - time step

* @return - the new calculated step size

*/

double epidemic_st::calculate_disease_ODEs( double step_size, list<leafLayer*>* layers, double t ){

double new_step_size = 0.0;

list<leafLayer*>::iterator it;

//we loop through all of our leaf layers here, and perform calculations for each leaf layer

for( it = layers->begin(); it != layers->end(); it ++ ){

leafLayer * layer_i = (*it);

//initialise our variable and derivative arrays for the leaf layer

initialise_calculation_arrays( layer_i );

}//end for each leaf layer

//now solve with RK4.

new_step_size = this->RK4_j( step_size, layers, t );

double leaf_extension_sum = 0.0;

//Give these newly calculated values back to each leaf layer

for( it = layers->begin(); it != layers->end(); it ++ ){

leafLayer * currentLayer = (*it);

//leaf_extensions<<"time "<<t<<" layer "<<currentLayer->getLayerNo()<<" extension "<<currentLayer->calc_extension( t )<<"\n";

if( !currentLayer->isActive() ){

continue;

}//end if

//lets sum up total plant extension

leaf_extension_sum += currentLayer->calc_extension( t );

set_new_layer_values( currentLayer, t );

}//end for

//assign the new total extension to the static member variable of leafLayer (for debugging only)

leafLayer::totalExtension = leaf_extension_sum;

return new_step_size;

}//end function

/**

* Takes our newly calculated variables and places them into the current

* leaf layer

*

* @param layer_i - leaf layer of interest

* @param t - current time (for debugging)

*

*/

void epidemic_st::set_new_layer_values( leafLayer * layer_i, double t ){

//get our arrays from our map

vector<long double> * variable_array = layer_temp_variable_map.at( layer_i );

layer_i->setArea( variable_array->at( parameters::Ai ) );

layer_i->setHealthyArea( variable_array->at( parameters::Hi ) );

layer_i->setInfectious_sensitive( variable_array->at( parameters::Ii_s ) );

layer_i->setInfectious_resistant( variable_array->at( parameters::Ii_r ) );

//get original latent compartment lists and add new values

vector<double> * layer_i_sensitive_latent = layer_i->getSensitiveLatentCompartments();

vector<double> * layer_i_resistant_latent = layer_i->getResistantLatentCompartments();

layer_i_sensitive_latent->clear();

layer_i_resistant_latent->clear();

layer_i_sensitive_latent->push_back( variable_array->at( parameters::L1i_s ) );

layer_i_resistant_latent->push_back( variable_array->at( parameters::L1i_r ) );

for( unsigned int i = parameters::L2i_s; i < parameters::L2i_r; i ++ ){

layer_i_sensitive_latent->push_back( variable_array->at( i ) );

}//end for

for( unsigned int i = parameters::L2i_r; i < parameters::Nseed_h; i ++ ){

layer_i_resistant_latent->push_back( variable_array->at( i ) );

}//end for

/**

* now set the fungicide specific values (I know I know, we are doing

* this multiple times for Nseed in the fungicide object!!).

*/

layer_i->set_high_risk_seed_treat_amount( variable_array->at( parameters::N_layer_i_h ) );

high_fung.set_Nseed( variable_array->at( parameters::Nseed_h ) );

layer_i->set_low_risk_seed_treat_amount( variable_array->at( parameters::N_layer_i_l ) );

low_fung.set_Nseed( variable_array->at( parameters::Nseed_l ) );

}//end function

/**

* Calculates the probability of transmission between the input leaf layers i and j.

* if the layers are not immediately adjacent to each other we will need the

* list of leaf layers to sum the distances between all layers between i and j.

*

* @param layer_i - target leaf layer

* @param layer_j - transmitting leaf layer

* @param layers - list of all other leaf layers

* @param t - current time step

*

* we do not normalise these probabilities, as spores can be lost to the ground. However, if a

* leaf may infect itself with 100% probability, then it cannot infect others. A scaling parameter

* exists that is multiplied with the transmission rate parameter to account for this (is part of

* the 'compound parameter').

*/

double epidemic_st::calc_prob_of_transmission( leafLayer * layer_i, leafLayer * layer_j, list<leafLayer*>* layers, double t ){

double dist = 0.0;

/**

* LAYERS CAN SELF INFECT THEMSELVES.

* if so we return a probability of 100%

*/

if( layer_j->getLayerNo() == layer_i->getLayerNo() ){

double prob = 1;

return( prob );

}else if( layer_i->getLayerNo() < layer_j->getLayerNo() ){

/**

* check if layer i is above layer j (note that the highest leaf has

* the smallest layer no, with the flag leaf being layer number 1)

*/

dist = layer_i->calc_extension( t );

list<leafLayer*>::iterator it;

for( it = layers->begin(); it != layers->end(); it ++ ){

leafLayer * currentLayer = (*it);

/**

* so as layer i is above layer j ( i < j ) we want layers that are less

* than layer i but greater than layer j

*/

if( currentLayer->getLayerNo() < layer_j->getLayerNo() &&

currentLayer->getLayerNo() > layer_i->getLayerNo() ){

dist += currentLayer->calc_extension( t );

}//end if

}//end for

double prob = exp( -parameters::sigma_up * dist );

return( prob );

/**

* check if layer j is above layer i (note that the highest leaf has

* the smallest layer no, with the flag leaf being layer number 1

*/

}else if( layer_i->getLayerNo() > layer_j->getLayerNo() ){

dist = layer_j->calc_extension( t );

list<leafLayer*>::iterator it;

for( it = layers->begin(); it != layers->end(); it ++ ){

leafLayer * currentLayer = (*it);

/**

* so as layer j is above layer i ( j < i ) we want layers that are greater

* than layer j but less than layer i

*/

if( currentLayer->getLayerNo() > layer_j->getLayerNo() &&

currentLayer->getLayerNo() < layer_i->getLayerNo() ){

dist += currentLayer->calc_extension( t );

}//end if

}//end for

double prob = exp( -parameters::sigma_down * dist );

return( prob );

}else{

cout<<"Error, we shouldn't be here!\n";

exit( 1 );

}

}//end function*/

/**

* Calculates the sum of all inoculum hitting leaf i from all other leaves j, including

* from leaf i.

* This is the product of the exponentially distributed probability of infection with distance

* between layers i and j and the infectious area index on leaf j.

*

* @param transmissionSum_s - sum of inoculum from the sensitive strain

* @param transmissionSum_r - sum of inoculum from the resistant strain

* @param layerIi - the current leaf layer of interest

* @param layers - list object of all leaf layers

* @param t - current time step.

*

* As transmissionSum_s and transmissionSum_r are references their values will be updated

* within the calling function.

*/

void epidemic_st::sum_infectious_spores( double & transmissionSum_s, double & transmissionSum_r,

leafLayer * layer_i, list<leafLayer*>* layers, double t ){

list<leafLayer*>::iterator it;

for( it = layers->begin(); it != layers->end(); it ++ ){

leafLayer * layer_j = (*it);

//check that layer_j has started growing, has initialised, or don't bother.

if( !layer_j->isActive() ){

continue;

}//end if*/

//get our initial conditions for layer j, so we can get sensitive and infectious area for layer_j

vector<long double> * variable_array_layer_j = layer_temp_variable_map.at( layer_j );

double sensitive_infectious_area_layer_j = variable_array_layer_j->at( parameters::Ii_s );

double resistant_infectious_area_layer_j = variable_array_layer_j->at( parameters::Ii_r );

transmissionSum_s += ( epidemic_st::calc_prob_of_transmission( layer_i, layer_j, layers, t )

* sensitive_infectious_area_layer_j );

transmissionSum_r += ( epidemic_st::calc_prob_of_transmission( layer_i, layer_j, layers, t )

* resistant_infectious_area_layer_j );

}//end for

}//end function

/**

* Calculates the healthy area of the input leaf layer i using our ODE.

* first calculates transmission of inoculum between leaf layers using our

* static function calc_prob_of_transmission(), then plugs this into the

* ODE.

*

* assumed to be run from within a loop, as we are looping through all leaf

* layers (hence the postfix 'i').

*

* @param layer_i - our leaf layer i of interest

* @param layer_j - pointer to our array of leaf layers, used for calculating

* the transmission of inoculum between the layers.

* @param t - Current time step. Used for calculating layer senescence.

*

*/

void epidemic_st::calc_healthy_area( leafLayer * layer_i, list<leafLayer*>* layers, double t ){

//get our arrays from our map

vector<long double> * variable_array = layer_temp_variable_map.at( layer_i );

vector<long double> * derivative_array = layer_derivative_map.at( layer_i );

double area = variable_array->at( parameters::Ai );

double healthy_area = variable_array->at( parameters::Hi );

//first we need to calculate Ai for the leaf layer

derivative_array->at( parameters::Ai ) = layer_i->calc_Ai_deriv( t, area );

//next we sum all spores received by leaf from itself and all other layers

double transmissionSum_s = 0.00;

double transmissionSum_r = 0.00;

sum_infectious_spores( transmissionSum_s, transmissionSum_r, layer_i, layers, t );

//lets get our variables for clarity:ins

double healthyRatio = 0.0;

if( area > 0.0 && healthy_area > 0.0 ){

healthyRatio = healthy_area / area;

}

double rho_s = fungicide_st::calc_reduction_in_rho_sensitive( high_fung, low_fung, layer_i, layers, t, layer_temp_variable_map );

double rho_r = fungicide_st::calc_reduction_in_rho_resistant( high_fung, low_fung, layer_i, layers, t, layer_temp_variable_map );

double eps_s = fungicide_st::calc_reduction_in_epsilon_sensitive( high_fung, low_fung, t, calc_X_t( t ), layer_i, layers, layer_temp_variable_map );

double eps_r = fungicide_st::calc_reduction_in_epsilon_resistant( high_fung, low_fung, t, calc_X_t( t ), layer_i, layers, layer_temp_variable_map );

double s_conidia = rho_s*healthyRatio*transmissionSum_s;

double r_conidia = rho_r*healthyRatio*transmissionSum_r;

double s_asc = eps_s*healthyRatio*(1-prop_resistant);

double r_asc = eps_r*healthyRatio*prop_resistant;

double senes = layer_i->senes_eq( t ) * healthy_area;

derivative_array->at( parameters::Hi ) = derivative_array->at( parameters::Ai ) - s_conidia - r_conidia - r_asc - s_asc - senes;

}//end function

/**

* Calculates the area of the first latent compartment of the input leaf layer i

* using our ODE. First calculates transmission of inoculum between leaf layers using our

* static function calc_prob_of_transmission(), then plugs this into the ODE.

*

* Assumed to be run from within a loop, as we are looping through all leaf

* layers (hence the postfix 'i').

*

* @param layer_i - our leaf layer i of interest

* @param layer_j - pointer to our array of leaf layers, used for calculating

* the transmission of inoculum between the layers.

* @param t - current time step. Used for calculating layer senescence.

*

*/

void epidemic_st::calc_first_latent_class( leafLayer * layer_i, list<leafLayer*>* layers, double t ){

//get our arrays from our map

vector<long double> * variable_array = layer_temp_variable_map.at( layer_i );

vector<long double> * derivative_array = layer_derivative_map.at( layer_i );

//first we need to sum all transmission from layer_i to all other layers

double transmissionSum_s = 0.00;

double transmissionSum_r = 0.00;

sum_infectious_spores( transmissionSum_s, transmissionSum_r, layer_i, layers, t );

//lets get our variables for clarity:

double healthyRatio = 0.0;

double area = variable_array->at( parameters::Ai );

double healthy_area = variable_array->at( parameters::Hi );

if( area > 0.0 && healthy_area > 0.0 ){

healthyRatio = healthy_area / area;

}//end if

double m = parameters::no_of_latent_compartments;

double senes = layer_i->senes_eq( t );

double rho_s = fungicide_st::calc_reduction_in_rho_sensitive( high_fung, low_fung, layer_i, layers, t, layer_temp_variable_map );

double rho_r = fungicide_st::calc_reduction_in_rho_resistant( high_fung, low_fung, layer_i, layers, t, layer_temp_variable_map );

double eps_s = fungicide_st::calc_reduction_in_epsilon_sensitive( high_fung, low_fung, t, calc_X_t( t ), layer_i, layers, layer_temp_variable_map );

double eps_r = fungicide_st::calc_reduction_in_epsilon_resistant( high_fung, low_fung, t, calc_X_t( t ), layer_i, layers, layer_temp_variable_map );

double delta_s = fungicide_st::calc_reduction_in_delta_sensitive( high_fung, low_fung, layer_i, layers, t, layer_temp_variable_map );

double delta_r = fungicide_st::calc_reduction_in_delta_resistant( high_fung, low_fung, layer_i, layers, t, layer_temp_variable_map );

double s_conidia = rho_s*healthyRatio*transmissionSum_s;

double r_conidia = rho_r*healthyRatio*transmissionSum_r;

double s_asc = eps_s*healthyRatio*(1-prop_resistant);

double r_asc = eps_r*healthyRatio*prop_resistant;

double s_latent_movement = m*delta_s*variable_array->at( parameters::L1i_s );

double s_latent_senescence = senes*variable_array->at( parameters::L1i_s );

double r_latent_movement = m*delta_r*variable_array->at( parameters::L1i_r );

double r_latent_senescence = senes*variable_array->at( parameters::L1i_r );

//ordinary differential equation for sensitive latent first compartment area

derivative_array->at( parameters::L1i_s ) = s_conidia + s_asc - s_latent_movement - s_latent_senescence;

//ordinary differential equation for resistant latent first compartment area

derivative_array->at( parameters::L1i_r ) = r_conidia + r_asc - r_latent_movement - r_latent_senescence;

}//end function

/**

* loops through all of our latent classes and calculates the derivatives

* for all latent classes excluding the first latent class which is calculated

* elsewhere.

*

* @param layer_i - our leaf layer i of interest

* @param t - current time step. Used for calculating layer senescence.

* @param layers - list of leaf layer objects

*

*/

void epidemic_st::calc_latent_classes( leafLayer * layer_i, double t, list<leafLayer*>* layers ){

//get our arrays from our map

vector<long double> * variable_array = layer_temp_variable_map.at( layer_i );

vector<long double> * derivative_array = layer_derivative_map.at( layer_i );

//lets get our variables for clarity:

double m = parameters::no_of_latent_compartments;

double input_delta_S = 0; //the parameter for inflow of sensitive latent area index to the current compartment

double output_delta_S = 0; //the parameter for outflow of sensitive latent area index from the current compartment to the next

double input_delta_R = 0; //the parameter for inflow of resistant latent area index to the current compartment

double output_delta_R = 0; //the parameter for outflow of resistant latent area index from the current compartment to the next

double senes = layer_i->senes_eq( t );

//calculate sensitive latent areas, loop from position L2i_s ( 6 ) to L2i_r ( 15 )

for( unsigned int i = parameters::L2i_s; i < parameters::L2i_r; i ++ ){

/**

* Bear in mind that if a latent compartment n > m/2, where

* m is the maximum number of compartments, fungicide doesn't

* have an effect. Note that in this function we are starting

* from the second latent compartment - hence we want fungicide

* to stop having an effect after half of the number of our

* latent compartments minus one.

*

* different delta_s out and delta_s in for intermediate classes!

*/

/**

* the compartment number is i - 4. L2i_s should be 6. The last bookeeping variable index is 5, however,

* we start here from compartment 2.

*/

int compartment_number = i - 4;

if( compartment_number < 6 ){

input_delta_S = fungicide_st::calc_reduction_in_delta_sensitive( high_fung, low_fung, layer_i, layers, t, layer_temp_variable_map );

output_delta_S = input_delta_S;

}else if( compartment_number == 6 ){

input_delta_S = fungicide_st::calc_reduction_in_delta_sensitive( high_fung, low_fung, layer_i, layers, t, layer_temp_variable_map );

output_delta_S = parameters::delta_default;

}else{

input_delta_S = parameters::delta_default;

output_delta_S = parameters::delta_default;

}//end else

double S_prev_latent = 0.0;

/**

* if class n is the second latent class, we take the previous to be the first latent class.

* otherwise, we take the n-1th class to be our previous class.

*/

if( i == parameters::L2i_s ){

S_prev_latent = m*input_delta_S*variable_array->at( parameters::L1i_s );

}else{

S_prev_latent = m*input_delta_S*variable_array->at( i-1 );

}

double movement_to_next_class_s = m*output_delta_S*variable_array->at( i );

double loss_to_senescence_s = senes*variable_array->at( i );

derivative_array->at( i ) = S_prev_latent - movement_to_next_class_s - loss_to_senescence_s;

}//end for

/**

* now calculate resistant latent areas. IF we are simulating absolute resistance, there should

* be NO DIFFERENCE between default_delta and delta after spraying.

*/

for( unsigned int i = parameters::L2i_r; i < parameters::Nseed_h; i ++ ){

/**

* the compartment number is i - 13. L2i_r should be 15. The last bookeeping variable index is 24, however,

* we start here from compartment 2.

*/

int compartment_number = i - 13;

if( compartment_number < 6 ){

input_delta_R = fungicide_st::calc_reduction_in_delta_resistant( high_fung, low_fung, layer_i, layers, t, layer_temp_variable_map );

output_delta_R = input_delta_R;

}else if( compartment_number == 6 ){

input_delta_R = fungicide_st::calc_reduction_in_delta_resistant( high_fung, low_fung, layer_i, layers, t, layer_temp_variable_map );

output_delta_R = parameters::delta_default;

}else{

input_delta_R = parameters::delta_default;

output_delta_R = parameters::delta_default;

}//end else

double R_prev_latent = 0.0;

/**

* if class n is the second latent class, we take the previous to be the first latent class.

* otherwise, we take the n-1th class to be our previous class.

*/

if( i == parameters::L2i_r ){

R_prev_latent = m*input_delta_R*variable_array->at( parameters::L1i_r );

}else{

R_prev_latent = m*input_delta_R*variable_array->at( i-1 );

}

double movement_to_next_class_r = m*output_delta_R*variable_array->at( i );

double loss_to_senescence_r = senes*variable_array->at( i );

derivative_array->at( i ) = R_prev_latent - movement_to_next_class_r - loss_to_senescence_r;

}//end for

}//end function//*/

/**

* Calculates the infectious area from sensitive and resistant strains.

* assumed to be run from within a loop.

*

* @param layer_i - our leaf layer of interest.

*

*/

void epidemic_st::calc_infected_area( leafLayer * layer_i, double t, list<leafLayer*>* layers ){

//get our arrays from our map

vector<long double> * variable_array = layer_temp_variable_map.at( layer_i );

vector<long double> * derivative_array = layer_derivative_map.at( layer_i );

//lets get our variables for clarity:

double m = parameters::no_of_latent_compartments;

double delta_S = fungicide_st::calc_reduction_in_delta_sensitive( high_fung, low_fung, layer_i, layers, t, layer_temp_variable_map );

double delta_R = fungicide_st::calc_reduction_in_delta_resistant( high_fung, low_fung, layer_i, layers, t, layer_temp_variable_map );

/**

* the element corresponding to the array element of the last sensitive latent compartment is the

* element for the resistant infectious compartment minus one. The element corresponding to the last resistant

* latent area is the element for seed treatment amount minus one

*/

double S_loss_of_last_latent = m*delta_S*variable_array->at( parameters::L2i_r - 1 );

double R_loss_of_last_latent = m*delta_R*variable_array->at( parameters::Nseed_h - 1 );

double S_loss_of_infectious = parameters::mu*variable_array->at( parameters::Ii_s );

double R_loss_of_infectious = parameters::mu*variable_array->at( parameters::Ii_r );

/**

* Remember that for the final latent compartment, array indices begin at zero and we

* have not placed the first latent compartment in our latent compartments array,

* therefore to access the final latent compartment from our array, Lm = array[m-2].

*/

//sensitive infectious area:

derivative_array->at( parameters::Ii_s ) = S_loss_of_last_latent - S_loss_of_infectious;

//resistant infectious area:

derivative_array->at( parameters::Ii_r ) = R_loss_of_last_latent - R_loss_of_infectious;

}//end method

/**

* takes an input vector and adds new ones inside, up to the

* input number of elements desired

*

* @param vectorIn - vector we wish to add more vectors inside

* @param no_of_elems - number of sub vectors we want in the outer vector

*/

void epidemic_st::make_new_arrayOfArrays( vector<vector<double>*> & vectorIn, int no_of_elems ){

for( int i = 0; i < no_of_elems; i ++ ){

vectorIn.push_back( new vector<double>( 1, 1.0 ) );

}

}

/**

* initialises our k vectors for at the beginning of the simulation.

* It appears for some reason that c++ doesn't like initialising arrays

* to zero, as they are still empty. So we add 1.0, which will be

* overwritten later

*

* @param no_leaf_layers - number of leaves we are simulating (11 probably)

*/

void epidemic_st::initialise_k_vectors( int no_leaf_layers ){

for( int i = 0; i < no_leaf_layers; i ++ ){k1.push_back( new vector<double>( 1, 1.0 ) );}

for( int i = 0; i < no_leaf_layers; i ++ ){k2.push_back( new vector<double>( 1, 1.0 ) );}

for( int i = 0; i < no_leaf_layers; i ++ ){k3.push_back( new vector<double>( 1, 1.0 ) );}

for( int i = 0; i < no_leaf_layers; i ++ ){k4.push_back( new vector<double>( 1, 1.0 ) );}

}

/**

* resets our k vectors for in between time steps, before

* RK4 integration

*/

void epidemic_st::reset_k_vectors(){

for( unsigned int i = 0; i < k1.size(); i ++ ){

k1[i]->clear();

k1[i]->push_back( 1.0 );

k2[i]->clear();

k2[i]->push_back( 1.0 );

k3[i]->clear();

k3[i]->push_back( 1.0 );

k4[i]->clear();

k4[i]->push_back( 1.0 );

}//end for

}//end function

/**

* checks the current time step against the spray times and the leaf layer initiation and

* death times, to see if there are any boundary conditions. If so, the smallest possible

* time step is returned.

*

* @param t - current time

* @param h - stepsize

* @param layers - list of leaf layer objects

*

* @return - smallest possiblem step size, where h t_boundary - t + 0.01

*/

double epidemic_st::check_for_boundary_conditions( double t, double h, list<leafLayer*>* layers ){

//make array for storing new h values. We'll return the smallest

vector<double> h_values;

//check for all leaf layers times of leaf initation and death

list<leafLayer*>::iterator it;

for( it = layers->begin(); it != layers->end(); it ++ ){

leafLayer * layer = (*it);

h_values.push_back( adjust_h( t, h, layer->get_t_ini() ) );

h_values.push_back( adjust_h( t, h, layer->get_t_death() ) );

//also check for extension

h_values.push_back( adjust_h( t, h, layer->get_t_emerge() ) );

}//end for

//now check spray times (they WILL be the same for high and low risk, as this is a mixture)

map<double, double> & spray_hash = high_fung.get_spray_times_hash();

map<double, double>::iterator map_it;

for( map_it = spray_hash.begin(); map_it != spray_hash.end(); map_it ++ ){

double spray_time = (*map_it).first;

h_values.push_back( adjust_h( t, h, spray_time ) );

}//end for

//now return the smallest value in the list

double smallest_h = 1000; //complete nonsense initial value

for( unsigned int i = 0; i < h_values.size(); i ++ ){

if( h_values.at( i ) < smallest_h ){smallest_h = h_values.at( i );}

}//end for

return smallest_h;

}//end method

/**

* Adjusts values of the step size according to any boundary time steps for RK4,

* where a sudden change in the model may occur, such as spraying fungicide.

* such an error can occur during RK4 as it calculates derivatives ahead of

* the current time step.

*

* if t+h > boundary_t, then h = boundary_t - t - 0.01 (so it is just under).

* Otherwise, the function will return the time step unadjusted.

*

* @param t - current time value

* @param h - current time step

* @param boundary_t - boundary time step of interest

*/

double epidemic_st::adjust_h( double t, double h, double boundary_t ){

double t_plus_h = t+h;

if( t < boundary_t && t_plus_h >= boundary_t ){

//we multiply up to round, then calculate the new step size

int large_boundary_t = boundary_t*parameters::h_MOD;

int large_time = t*parameters::h_MOD ;

//double new_step_size = boundary_t - t;

double new_step_size = ( large_boundary_t - large_time )/10.0;

//cout<<"large_time = "<<large_time<<" large_boundary_t = "<<large_boundary_t<<" h = "<<h<<" new_step_size = "<<new_step_size<<"\n";

//cout<<"";

return new_step_size;

}else{

return h;

}//end else

}//end method

/**

* Runge Kutta 4th order integrator for solving our ODEs.

* this is the more complicated algorithm, that calls all

* of our differential equations with different independent

* variable (y) values

*

* we have our variable array of initial conditions for our state

* variables. We also have a temp variable array, for each of the four

* iterations of Runge Kutta

*

* we first loop through all our leaves, copying our initial conditions

* to each variable, plus each weight (e.g., h/2*k1, h/2*k2, etc).

*

* we then calculate the derivatives based on these initial conditions, and

* place in the corresponding k array (k1, k2, k3, k4)

*

* we reset our temp variable array for each RK4 iteration

*

* after K4, we take the weighted average as the derivative and add to the value of

* the each state variable

*

* @param stepSize - step size

* @param layers - list of leaf objects

*

* @param t - current time step

*

* @return - new step size (as calculated by function

* check_for_boundary_conditions()

*/

double epidemic_st::RK4_j( double h, list<leafLayer*>* layers, double t ){

/*fstream h_in_rk4;

h_in_rk4.open( "D:/seed_treatment_sims/o_files/h_in_rk4.txt", ios::out | ios::app );

h_in_rk4<<"t = "<<t<<" input step size = "<<h<<endl;//*/

//initialise our k vectors

reset_k_vectors();

list<leafLayer*>::iterator it;

//adjust our step size according to any boundaries

double stepSize = 0.0;

stepSize = check_for_boundary_conditions( t, h, layers );

//h_in_rk4<<"t = "<<t<<" step size after boundary check = "<<stepSize<<endl;

//first copy initial conditions over to our temp initial condition array

for( it = layers->begin(); it != layers->end(); it ++ ){

leafLayer * layer = (*it);

if( !layer->isActive() ){continue;}

int layerNo = layer->getLayerNo()-1;

copyVectors( layer_variable_map.at( layer ), layer_temp_variable_map.at( layer ) );

}

//now calculate derivatives and place in k1

for( it = layers->begin(); it != layers->end(); it ++ ){

leafLayer * layer = (*it);

if( !layer->isActive() ){continue;}

int layerNo = layer->getLayerNo()-1;

derivs( layer, layers, t );

//place derivatives into array k1

copyVectors( layer_derivative_map.at( layer ), k1[layerNo] );

}

//first copy k1 initial conditions over to our temp initial condition array

for( it = layers->begin(); it != layers->end(); it ++ ){

leafLayer * layer = (*it);

if( !layer->isActive() ){continue;}

int layerNo = layer->getLayerNo()-1;

copyVectors( layer_variable_map.at( layer ), layer_temp_variable_map.at( layer ) );

copy_and_add_with_modifier( layer_temp_variable_map.at( layer ), k1[layerNo], stepSize*0.5 );

}

//now calculate derivatives and place in k2

for( it = layers->begin(); it != layers->end(); it ++ ){

leafLayer * layer = (*it);

if( !layer->isActive() ){continue;}

int layerNo = layer->getLayerNo()-1;

derivs( layer, layers, ( t + stepSize / 2 ) );

//place derivatives into array k2

copyVectors( layer_derivative_map.at( layer ), k2[layerNo] );

}

//first copy k2 initial conditions over to our temp initial condition array

for( it = layers->begin(); it != layers->end(); it ++ ){

leafLayer * layer = (*it);

if( !layer->isActive() ){continue;}

int layerNo = layer->getLayerNo()-1;

copyVectors( layer_variable_map.at( layer ), layer_temp_variable_map.at( layer ) );

copy_and_add_with_modifier( layer_temp_variable_map.at( layer ), k2[layerNo], stepSize*0.5 );

}

//now calculate derivatives and place in k3

for( it = layers->begin(); it != layers->end(); it ++ ){

leafLayer * layer = (*it);

if( !layer->isActive() ){continue;}

int layerNo = layer->getLayerNo()-1;

derivs( layer, layers, ( t + stepSize / 2 ) );

copyVectors( layer_derivative_map.at( layer ), k3[layerNo] );

}

//first copy k3 initial conditions over to our temp initial condition array

for( it = layers->begin(); it != layers->end(); it ++ ){

leafLayer * layer = (*it);

if( !layer->isActive() ){continue;}

int layerNo = layer->getLayerNo()-1;

copyVectors( layer_variable_map.at( layer ), layer_temp_variable_map.at( layer ) );

copy_and_add_with_modifier( layer_temp_variable_map.at( layer ), k3[layerNo], stepSize );

}

//now calculate derivatives and place in k4

for( it = layers->begin(); it != layers->end(); it ++ ){

leafLayer * layer = (*it);

if( !layer->isActive() ){continue;}

int layerNo = layer->getLayerNo()-1;

derivs( layer, layers, ( t + stepSize ) );

copyVectors( layer_derivative_map.at( layer ), k4[layerNo] );

}

//ok, now we sum and average through the k values

for( it = layers->begin(); it != layers->end(); it ++ ){

leafLayer * layer = (*it);

if( !layer->isActive() ){continue;}

int layerNo = layer->getLayerNo()-1;

vector<long double> * variable_array = layer_variable_map.at( layer );

vector<long double> * temp_variable_array = layer_temp_variable_map.at( layer );

/**

* we now iterate up to N_layer_i_l, as this is the last derivative

*/

for( unsigned int i = 0; i <= parameters::N_layer_i_l; i ++ ){

long double k_1 = k1[layerNo]->at( i );

long double k_2 = k2[layerNo]->at( i );

long double k_3 = k3[layerNo]->at( i );

long double k_4 = k4[layerNo]->at( i );

long double old_var = variable_array->at( i );

long double new_var = old_var + (((k_1) + (2*k_2) + (2*k_3) + (k_4))*stepSize)/6;

temp_variable_array->at( i ) = new_var;

}//end for

}//end for

//now swap across temp to variable array

for( it = layers->begin(); it != layers->end(); it ++ ){

leafLayer * layer = (*it);

if( !layer->isActive() ){continue;}

vector<long double> * variable_array = layer_variable_map.at( layer );

vector<long double> * temp_variable_array = layer_temp_variable_map.at( layer );

copyVectors( temp_variable_array, variable_array );

}

if( stepSize != h ){

//h_in_rk4<<"t = "<<t<<" returning "<<stepSize<<"\n\n";

//h_in_rk4.close();

return stepSize;

}else{

//h_in_rk4<<"t = "<<t<<" returning "<<h<<"\n\n";

//h_in_rk4.close();

return h;

}//end else

}//end method*/

**epidemic_st.h**

#ifndef EPIDEMIC_H

#define EPIDEMIC_H

#include <iostream>

#include <vector>

#include <map>

#include <list>

#include <fstream>

#include "leafLayer.h"

#include "fungicide_st.h"

class epidemic_st{

private:

fungicide_st & high_fung; //fungicide object, allowing us to access the ODE's stored in its function definitions

fungicide_st & low_fung; //fungicide object, allowing us to access the ODE's stored in its function definitions

double prop_resistant;

double epsilon_asco; //transmission rate for ascospores

std::map<leafLayer*, std::vector<long double>*> layer_variable_map; //for calculating the dependent variables of our ODE's.

std::map<leafLayer*, std::vector<long double>*> layer_temp_variable_map; //for calculating the dependent variables of our ODE's.

std::map<leafLayer*, std::vector<long double>*> layer_derivative_map; //for calculating the dependent variables of our ODE's.

std::vector<std::vector<double>*> k1; //our k vectors for runge kutta. Columns are the leaf layer numbers

std::vector<std::vector<double>*> k2; //(minus one), rows are the layer variables, e.g. Ai, Hi, Li, Ii...etc

std::vector<std::vector<double>*> k3;

std::vector<std::vector<double>*> k4;

void RK4( double stepSize, std::list<leafLayer*>* layers, double t ); //Runge Kutta 4th order integrator used to numerically solve

//our differential equations

double RK4_j( double h, std::list<leafLayer*>* layers, double t ); //Runge Kutta 4th order integrator used to numerically solve

//our differential equations

void derivs( leafLayer * layer_i, std::list<leafLayer*>* layers, double t ); //function containing our derivatives

//static methods

static void make_new_arrayOfArrays( std::vector<std::vector<double>*> & vectorIn, int no_of_elems );

static double calc_prob_of_transmission( leafLayer * layer_i, leafLayer * layer_j, std::list<leafLayer*>* layers, double t );

//calculation methods

void calc_healthy_area( leafLayer * layer_i, std::list<leafLayer*>* layers, double t );

void calc_first_latent_class( leafLayer * layer_i, std::list<leafLayer*>* layers, double t );

void calc_latent_classes( leafLayer * layer_i, double t, std::list<leafLayer*>* layers );

void calc_infected_area( leafLayer * layer_i, double t, std::list<leafLayer*>* layers );

void initialise_calculation_arrays( leafLayer * layer_i );

void set_new_layer_values( leafLayer * layer_i, double t );

void initialise_k_vectors( int no_leaf_layers );

void reset_k_vectors();

void sum_infectious_spores( double & transmissionSum_s, double & transmissionSum_r,

leafLayer * layer_i, std::list<leafLayer*>* layers, double t );

void sum_infectious_spores_normalised( double & transmissionSum_s, double & transmissionSum_r,

leafLayer * layer_i, std::list<leafLayer*>* layers, double t );

double check_for_boundary_conditions( double t, double h, std::list<leafLayer*>* layers );

double adjust_h( double t, double h, double boundary_t );

public:

//constructor

epidemic_st( std::list<leafLayer*>* layers, fungicide_st & h_fungicideIn, fungicide_st & l_fungicideIn );

double calculate_disease_ODEs( double step_size, std::list<leafLayer*>* layers, double t );

void calc_asco_influx( double t, std::list<leafLayer*>* layers );

double calc_X_t( double t );

void calc_prop_of_resistant( std::list<leafLayer*>*, std::fstream & res_prop );

void calc_prop_of_resistant( double & sum_sensitive, double & sum_resistant, std::fstream & res_prop );

};

#endif

**foliar_spray_fungicide.cpp**

#include "foliar_spray_fungicide.h"

#include "parameters.h"

using namespace std;

foliar_spray_fungicide::foliar_spray_fungicide( const double & foliar_breakdownIn ) {

foliar_breakdown = foliar_breakdownIn;

//cout<<"foliar_breakdown "<<foliar_breakdown<<endl;

}

/**

* Calculates the per unit area volume of fungicide within the leaf layer and the

* amount that has degraded according to an exponential decay model.

*

* @param layer_i - leaf layer as to what ODE's we are currently solving

* @param layers - list of leaf layer objects

* @param t - current time

* @param layer_temp_variable_map - hash table containing initial conditions per leaf layer of our ODE's.

* @param leaf_spray_doses - hash table containing initial dose of high risk/low risk fungicide

*

*

* @return - new concentration after some breakdown has occured.

*/

double foliar_spray_fungicide::calculate_concentration_after_decay( leafLayer * layer_i, list<leafLayer*>* layers,

double t, map<leafLayer*, vector<long double>*> & layer_temp_variable_map,

map<double,double> * leaf_spray_doses ){

vector<long double> * layer_var_array = layer_temp_variable_map.at( layer_i );

double leaf_area = layer_var_array->at( parameters::Ai );

double q = parameters::leaf_thickness;

double sum_of_doses = 0.0; //doses before degredation is taken into account

double new_concentration = 0.0; //concentration after degredation is taken into account

//now we sum all the doses on spraying at previous sprays

map<double,double>::iterator it;

for( it = leaf_spray_doses->begin(); it != leaf_spray_doses->end(); it ++ ){

double t_spray = (*it).first;

double previous_dose_on_spraying = leaf_spray_doses->at( t_spray );

double eVal = exp( -this->foliar_breakdown*( t - t_spray ) );

sum_of_doses += previous_dose_on_spraying * eVal ;

}

//ensure we don't divide by zero

if( leaf_area*q != 0 ){

new_concentration = sum_of_doses / ( leaf_area*q );

}

return new_concentration;

}//*/

/**

* Calculates the most recent spray dose according to t.

* to do this, we take the largest spray event in our list, t_s_max, where t_s_max < t

*

* @param t - current time step

* @return - current dose (as a fraction of the max dose).

*

*/

double foliar_spray_fungicide::get_most_recent_spray_dose( double t ){

double last_spray = 0.0;

map<double, double>::iterator it;

/**

* we assume that the times written in the list are in advancing chronological order,

* e.g. 100, 200, 400, 600, and are not randomly ordered

*/

for( it = spray_times.begin(); it != spray_times.end(); it ++ ){

double time = (*it).first;

if( time > last_spray && time <= t ){

last_spray = time;

}

}//end for

return spray_times[last_spray];

}//end function

/**

* calculates the most recent spray time according to t.

* to do this, we take the largest spray event in our list, t_s_max, where t_s_max < t

*

* @param t - current time step

* @return - current dose (as a fraction of the max dose).

*

*/

double foliar_spray_fungicide::get_most_recent_spray_time( double t ){

map<double, double>::iterator it;

/**

* We assume that the times written in the list are in advancing chronological order,

* e.g. 100, 200, 400, 600, and are not randomly ordered

*/

double last_spray = 0.0;

for( it = spray_times.begin(); it != spray_times.end(); it ++ ){

double time = (*it).first;

if( time > last_spray && time <= t ){

last_spray = time;

}

}//end for

return last_spray;

}//end function

/**

* Calculates the amount of fungicide spray that is received by each leaf layer i, after

* loss from the interception of all other leaf layers j

*

* @param layer_i - leaf layer as to what ODE's we are currently solving

* @param layers - list of leaf layer objects

* @param t - current time

* @param layer_temp_variable_map - hash table containing initial conditions per leaf layer of our ODE's.

* @param Area_element_no - element number in array of inititial conditions, per leaf, that contains

* the initial condition for leaf area

* @return - the dose that leaf layer i received after interception by the other layers

* - this can be summed up by the calling function

*/

double foliar_spray_fungicide::calculate_dose_per_leaf_on_spraying( leafLayer * layer_i, list<leafLayer*>* layers, double t ){

list<leafLayer*>::iterator it_j;

double leaf_i_area = layer_i->getArea();

/**

* so the below variable geometrically sums the probability of fungicide

* not being intercepted by layers higher up than layer j ( layer_j

* number < layer_i number). E.g., if layer i is layer 3, then

* we sum probabilities of interception for layers 2 and 1.

*/

double sum_of_higher_layers = 1.0; //as we are taking the geometric sum we can initialise to one

for( it_j = layers->begin(); it_j != layers->end(); it_j ++ ){

leafLayer * layer_j = (*it_j);

if( !layer_j->isActive() ){

continue;

}

double leaf_j_area = layer_j->getArea();

if( layer_j->getLayerNo() < layer_i->getLayerNo() ){

sum_of_higher_layers *= exp( -parameters::tau * leaf_j_area );

}//end if

}//end for

double prob_of_interception = 1 - exp( -parameters::tau * leaf_i_area );

//get our dose sprayed at this time

double f_0 = spray_times[floor(t)];

double f_i = f_0 * prob_of_interception * sum_of_higher_layers;

return f_i;

}//end function

/**

* reads spray time and dose information in, according to

* the format <time> <dose>

*

* e.g. 1000 0.7

* 2000 0.7

*/

void foliar_spray_fungicide::read_fungicide_dose_data(){

cout<<"reading in foliar spray times\n";

string line;

ifstream myFile ("foliar_times.txt");

if( myFile.is_open() ){

while( myFile.good () ){

getline( myFile, line );

//split up according to single white spaces:

cout<<"line = "<<line<<endl;

vector<string> out = split( line, ' ' );

string word_one;

string word_two;

try{

word_one = out.at( 0 );

word_two = out.at( 1 );

}catch( out_of_range &e ){

continue;

}//end catch

spray_times.insert( make_pair( atof( word_one.c_str() ), atof( word_two.c_str() ) ) );

string ans;

cout<<"\n\n\n************************\nspray dose = "<<word_one<<" - "<<word_two<<"\nAre you happy? y\\n\n";

cin>>ans;

if( ans == "n" ){exit( 0 );}

}//end while

if( spray_times.empty() ){

cout<<"Error - no spray times read. Set to 0 if you want no spray, but do not have a blank foliar spray file.\n";

exit( 1 );

}

}//end if

cout<<"Done reading foliar spray times\n";

}//end method

/**

* reads spray time and dose information in, according to

* the format <time> <dose>

*

* e.g. 1000 0.7

* 2000 0.7

*/

void foliar_spray_fungicide::read_fungicide_dose_data( string & fileName ){

cout<<"reading in foliar spray times from file "<<fileName<<"\n";

string line;

ifstream myFile ( fileName );

if( myFile.is_open() ){

while( myFile.good () ){

getline( myFile, line );

//split up according to single white spaces:

cout<<"line = "<<line<<endl;

vector<string> out = split( line, ' ' );

string word_one;

string word_two;

try{

word_one = out.at( 0 );

word_two = out.at( 1 );

}catch( out_of_range &e ){

continue;

}//end catch

spray_times.insert( make_pair( atof( word_one.c_str() ), atof( word_two.c_str() ) ) );

}//end while

if( spray_times.empty() ){

cout<<"Error - no spray times read. Set to 0 if you want no spray, but do not have a blank foliar spray file.\n";

exit( 1 );

}

}else{

cout<<"Error in reading foliar file\n";

exit( 1 );

}

cout<<"Done reading foliar spray times\n";

}//end method

**foliar_spray_fungicide.h**

#ifndef FOLIAR_SPRAY_FUNGICIDE_H

#define FOLIAR_SPRAY_FUNGICIDE_H

#include <map>

#include <iostream>

#include <istream>

#include <fstream>

#include <string>

#include <math.h>

#include "leafLayer.h"

#include "JKUtils.h"

class foliar_spray_fungicide{

private:

std::map<double, double> spray_times; //map containing time in degree days of spraying and the dose

double foliar_breakdown;

public:

foliar_spray_fungicide(){}

foliar_spray_fungicide( const double & foliar_breakdownIn );

void read_fungicide_dose_data(); //reads in our fungicide dose and timing data

void read_fungicide_dose_data( std::string & fileName ); //reads in our fungicide dose and timing data according to the input file

std::map<double, double> & get_spray_times_hash(){

return spray_times;

}

double get_most_recent_spray_dose( double t ); //retrieves the dose that was sprayed on the field at the last spray

double get_most_recent_spray_time( double t ); //retrieves the time of the last spray

double calculate_dose_per_leaf_on_spraying( leafLayer * layer_i, std::list<leafLayer*>* layers, double t );

protected:

double calculate_concentration_after_decay( leafLayer * layer_i, std::list<leafLayer*>* layers, double t,

std::map<leafLayer*, std::vector<long double>*> & layer_temp_variable_map,

std::map<double,double> * leaf_spray_doses );

};

#endif

**fungicide_st.cpp**

#include "fungicide_st.h"

#include "epidemic_st.h"

#include "parameters.h"

using namespace std;

/**

* Constructor for the fungicide_st object.

*

* @param foliar_breakdownIn - breakdown rate parameter for spray fungicides

* @param ST_breakdownIn - breakdown rate parameter for seed treatments

* @param ST_uptakeIn - leaf uptake rate parameter for seed treatments

* @param N_seed_element_noIn - book-keeping index variable for where in our derivative

* / variable arrays the amount of ST left in the seed is stored

* @param N_layer_element_noIn - book-keeping index variable for where in our derivative

* / variable arrays the amount of ST left in the leaf is stored

*

* all the above parameters are passed by reference into the seed_treatment_fungicide and foliar_spray_fungicide

* parent objects.

*/

fungicide_st::fungicide_st( const double & foliar_breakdownIn, const double & ST_breakdownIn, const double & ST_uptakeIn,

unsigned int & N_seed_element_noIn, unsigned int N_layer_element_noIn )

: seed_treatment_fungicide( ST_breakdownIn, ST_uptakeIn, N_seed_element_noIn, N_layer_element_noIn ),

foliar_spray_fungicide( foliar_breakdownIn ){

/*cout<<"\nfoliar_breakdownIn "<<foliar_breakdownIn;

cout<<"\nST_breakdownIn "<<ST_breakdownIn;

cout<<"\nST_uptakeIn "<<ST_uptakeIn;

cout<<"\nN_seed_element_noIn "<<N_seed_element_noIn;

cout<<"\nN_layer_element_noIn "<<N_layer_element_noIn;//*/

}

/**

* calculates the reduction in the probability of moving between different latent compartments

* according to the amount of fungicide accrued per leaf layer, for the sensitive strain.

*

* @param layer_i - leaf layer to which we are solving our ODE's

* @param layer_temp_variable_map - hash table containing leaf layers as keys and arrays of the

* plant growth and disease compartment variables

* @param layers - list of leaf layer objects

* @param t - current time step

* seed treatment fungicide is being kept.

*/

double fungicide_st::calc_reduction_in_delta_sensitive( fungicide_st & high_fung, fungicide_st & low_fung,

leafLayer * layer_i, std::list<leafLayer*>* layers,

double t, std::map<leafLayer*, std::vector<long double>*> & layer_temp_variable_map ){

double foliar_conc_h = high_fung.calculate_concentration_after_decay( layer_i, layers, t, layer_temp_variable_map, layer_i->get_high_risk_foliar_dose_at_sprays() );

double ST_conc_h = high_fung.calc_seed_treat_concentration( layer_i, layers, layer_temp_variable_map, t );

double foliar_conc_l = low_fung.calculate_concentration_after_decay( layer_i, layers, t, layer_temp_variable_map, layer_i->get_low_risk_foliar_dose_at_sprays() );

double ST_conc_l = low_fung.calc_seed_treat_concentration( layer_i, layers, layer_temp_variable_map, t );

double alpha_h = parameters::alpha_delta_h*( 1 - exp( -parameters::k_delta_s_h*( foliar_conc_h + ST_conc_h ) ) );

double alpha_l = parameters::alpha_delta_l*( 1 - exp( -parameters::k_delta_s_l*( foliar_conc_l + ST_conc_l ) ) );

return parameters::delta_default * ( 1 - alpha_h ) * ( 1 - alpha_l );

}

/**

* calculates the reduction in the ascospore transmission rate

* according to the amount of fungicide accrued per leaf layer for the sensitive strain.

*

* @param layer_i - leaf layer to which we are solving our ODE's

* @param layer_temp_variable_map - hash table containing leaf layers as keys and arrays of the

* plant growth and disease compartment variables

* @param layers - list of leaf layer objects

* @param t - current time step

* @param X_t - variable corresponding to the amount of ascospore influx. Used for

* the transmission rate compound parameter calculation.

*/

double fungicide_st::calc_reduction_in_epsilon_sensitive( fungicide_st & high_fung, fungicide_st & low_fung,

double t, double X_t, leafLayer * layer_i, std::list<leafLayer*>* layers,

std::map<leafLayer*, std::vector<long double>*> & layer_temp_variable_map ){

double foliar_conc_h = high_fung.calculate_concentration_after_decay( layer_i, layers, t, layer_temp_variable_map, layer_i->get_high_risk_foliar_dose_at_sprays() );

double ST_conc_h = high_fung.calc_seed_treat_concentration( layer_i, layers, layer_temp_variable_map, t );

double foliar_conc_l = low_fung.calculate_concentration_after_decay( layer_i, layers, t, layer_temp_variable_map, layer_i->get_low_risk_foliar_dose_at_sprays() );

double ST_conc_l = low_fung.calc_seed_treat_concentration( layer_i, layers, layer_temp_variable_map, t );

double alpha_h = parameters::alpha_epsilon_h*( 1 - exp( -parameters::k_epsilon_s_h*( foliar_conc_h + ST_conc_h ) ) );

double alpha_l = parameters::alpha_epsilon_l*( 1 - exp( -parameters::k_epsilon_s_l*( foliar_conc_l + ST_conc_l ) ) );

double epsilon_default = parameters::gamma_default * X_t;

double epsilon_sensitive = epsilon_default * ( 1 - alpha_h ) * ( 1 - alpha_l );

return epsilon_sensitive;

}

/**

* calculates the reduction in the conidial transmission rate

* according to the amount of fungicide accrued per leaf layer for the sensitive strain. Writes out results.

*

* @param layer_i - leaf layer to which we are solving our ODE's

* @param layer_temp_variable_map - hash table containing leaf layers as keys and arrays of the

* plant growth and disease compartment variables

* @param layers - list of leaf layer objects

* @param t - current time step

*

*/

double fungicide_st::calc_reduction_in_rho_sensitive( fungicide_st & high_fung, fungicide_st & low_fung,

leafLayer * layer_i, std::list<leafLayer*>* layers,

double t, std::map<leafLayer*, std::vector<long double>*> & layer_temp_variable_map ){

double foliar_conc_h = high_fung.calculate_concentration_after_decay( layer_i, layers, t, layer_temp_variable_map, layer_i->get_high_risk_foliar_dose_at_sprays() );

double ST_conc_h = high_fung.calc_seed_treat_concentration( layer_i, layers, layer_temp_variable_map, t );

double foliar_conc_l = low_fung.calculate_concentration_after_decay( layer_i, layers, t, layer_temp_variable_map, layer_i->get_low_risk_foliar_dose_at_sprays() );

double ST_conc_l = low_fung.calc_seed_treat_concentration( layer_i, layers, layer_temp_variable_map, t );

double alpha_h = parameters::alpha_rho_h*( 1 - exp( -parameters::k_rho_s_h*( foliar_conc_h + ST_conc_h ) ) );

double alpha_l = parameters::alpha_rho_l*( 1 - exp( -parameters::k_rho_s_l*( foliar_conc_l + ST_conc_l ) ) );

double rho_s = parameters::rho_default * ( 1 - alpha_h ) * ( 1 - alpha_l );

return rho_s;

}//end method

/**

* calculates the reduction in the probability of moving between different latent compartments

* according to the amount of fungicide accrued per leaf layer, for the sensitive strain.

*

* @param layer_i - leaf layer to which we are solving our ODE's

* @param layer_temp_variable_map - hash table containing leaf layers as keys and arrays of the

* plant growth and disease compartment variables

* @param layers - list of leaf layer objects

* @param t - current time step

* seed treatment fungicide is being kept.

*/

double fungicide_st::calc_reduction_in_delta_resistant( fungicide_st & high_fung, fungicide_st & low_fung,

leafLayer * layer_i, std::list<leafLayer*>* layers,

double t, std::map<leafLayer*, std::vector<long double>*> & layer_temp_variable_map ){

double foliar_conc_h = high_fung.calculate_concentration_after_decay( layer_i, layers, t, layer_temp_variable_map, layer_i->get_high_risk_foliar_dose_at_sprays() );

double ST_conc_h = high_fung.calc_seed_treat_concentration( layer_i, layers, layer_temp_variable_map, t );

double foliar_conc_l = low_fung.calculate_concentration_after_decay( layer_i, layers, t, layer_temp_variable_map, layer_i->get_low_risk_foliar_dose_at_sprays() );

double ST_conc_l = low_fung.calc_seed_treat_concentration( layer_i, layers, layer_temp_variable_map, t );

double alpha_l = parameters::alpha_delta_l*( 1 - exp( -parameters::k_delta_s_l*( foliar_conc_l + ST_conc_l ) ) );

return parameters::delta_default * ( 1 - alpha_l );

}

/**

* calculates the reduction in the ascospore transmission rate

* according to the amount of fungicide accrued per leaf layer for the sensitive strain.

*

* @param layer_i - leaf layer to which we are solving our ODE's

* @param layer_temp_variable_map - hash table containing leaf layers as keys and arrays of the

* plant growth and disease compartment variables

* @param layers - list of leaf layer objects

* @param t - current time step

* @param X_t - variable corresponding to the amount of ascospore influx. Used for

* the transmission rate compound parameter calculation.

*/

double fungicide_st::calc_reduction_in_epsilon_resistant( fungicide_st & high_fung, fungicide_st & low_fung,

double t, double X_t, leafLayer * layer_i, std::list<leafLayer*>* layers,

std::map<leafLayer*, std::vector<long double>*> & layer_temp_variable_map ){

double foliar_conc_h = high_fung.calculate_concentration_after_decay( layer_i, layers, t, layer_temp_variable_map, layer_i->get_high_risk_foliar_dose_at_sprays() );

double ST_conc_h = high_fung.calc_seed_treat_concentration( layer_i, layers, layer_temp_variable_map, t );

double foliar_conc_l = low_fung.calculate_concentration_after_decay( layer_i, layers, t, layer_temp_variable_map, layer_i->get_low_risk_foliar_dose_at_sprays() );

double ST_conc_l = low_fung.calc_seed_treat_concentration( layer_i, layers, layer_temp_variable_map, t );

double alpha_l = parameters::alpha_epsilon_l*( 1 - exp( -parameters::k_epsilon_s_l*( foliar_conc_l + ST_conc_l ) ) );

double epsilon_default = parameters::gamma_default * X_t;

double epsilon_resistant = epsilon_default * ( 1 - alpha_l );

return epsilon_resistant;

}

/**

* calculates the reduction in the conidial transmission rate

* according to the amount of fungicide accrued per leaf layer for the sensitive strain. Writes out results.

*

* @param layer_i - leaf layer to which we are solving our ODE's

* @param layer_temp_variable_map - hash table containing leaf layers as keys and arrays of the

* plant growth and disease compartment variables

* @param layers - list of leaf layer objects

* @param t - current time step

*

*/

double fungicide_st::calc_reduction_in_rho_resistant( fungicide_st & high_fung, fungicide_st & low_fung,

leafLayer * layer_i, std::list<leafLayer*>* layers,

double t, std::map<leafLayer*, std::vector<long double>*> & layer_temp_variable_map ){

double foliar_conc_h = high_fung.calculate_concentration_after_decay( layer_i, layers, t, layer_temp_variable_map, layer_i->get_high_risk_foliar_dose_at_sprays() );

double ST_conc_h = high_fung.calc_seed_treat_concentration( layer_i, layers, layer_temp_variable_map, t );

double foliar_conc_l = low_fung.calculate_concentration_after_decay( layer_i, layers, t, layer_temp_variable_map, layer_i->get_low_risk_foliar_dose_at_sprays() );

double ST_conc_l = low_fung.calc_seed_treat_concentration( layer_i, layers, layer_temp_variable_map, t );

double alpha_l = parameters::alpha_rho_l*( 1 - exp( -parameters::k_rho_s_l*( foliar_conc_l + ST_conc_l ) ) );

return parameters::rho_default * ( 1 - alpha_l );

}//end method

**Fungicide_st.h**

#ifndef FUNGICIDE_ST_H

#define FUNGICIDE_ST_H

#include <list>

#include <math.h>

#include <fstream>

#include "leafLayer.h"

#include "foliar_spray_fungicide.h"

#include "seed_treatment_fungicide.h"

class fungicide_st : public seed_treatment_fungicide, public foliar_spray_fungicide {

public:

//constructor

fungicide_st( const double & foliar_breakdownIn, const double & ST_breakdownIn, const double & ST_uptakeIn,

unsigned int & N_seed_element_noIn, unsigned int N_layer_element_noIn );

//copy constructor

fungicide_st( const fungicide_st& orig ){

std::cout<<"copy constructor called!\n";

}

void print_input_params();

//sensitive transmission rate functions

static double calc_reduction_in_rho_sensitive( fungicide_st & high_fung, fungicide_st & low_fung,

leafLayer * layer_i, std::list<leafLayer*>* layers, double t, std::map<leafLayer*,

std::vector<long double>*> & layer_temp_variable_map );

static double calc_reduction_in_epsilon_sensitive( fungicide_st & high_fung, fungicide_st & low_fung,

double t, double X_t, leafLayer * layer_i, std::list<leafLayer*>* layers,

std::map<leafLayer*, std::vector<long double>*> & layer_temp_variable_map );

static double calc_reduction_in_delta_sensitive( fungicide_st & high_fung, fungicide_st & low_fung,

leafLayer * layer_i, std::list<leafLayer*>* layers,

double t, std::map<leafLayer*, std::vector<long double>*> & layer_temp_variable_map );

//resistant transmission rate functions

static double calc_reduction_in_rho_resistant( fungicide_st & high_fung, fungicide_st & low_fung,

leafLayer * layer_i, std::list<leafLayer*>* layers, double t, std::map<leafLayer*,

std::vector<long double>*> & layer_temp_variable_map );

static double calc_reduction_in_epsilon_resistant( fungicide_st & high_fung, fungicide_st & low_fung,

double t, double X_t, leafLayer * layer_i, std::list<leafLayer*>* layers,

std::map<leafLayer*, std::vector<long double>*> & layer_temp_variable_map );

static double calc_reduction_in_delta_resistant( fungicide_st & high_fung, fungicide_st & low_fung,

leafLayer * layer_i, std::list<leafLayer*>* layers,

double t, std::map<leafLayer*, std::vector<long double>*> & layer_temp_variable_map );

};

#endif

**JKUtils.h**

#ifndef JKUtils_H

#define JKUtils_H

#include <vector>

#include <sstream>

#include <string>

#include <map>

/**

* The below functions are for splitting up a string according

* white space

*/

static std::vector<std::string>& split(const std::string &s, char delim, std::vector<std::string> &elems) {

std::stringstream ss(s);

std::string item;

while (std::getline(ss, item, delim)) {

elems.push_back(item);

}

return elems;

}

static std::vector<std::string> split(const std::string &s, char delim) {

std::vector<std::string> elems;

split(s, delim, elems);

return elems;

}

template< typename T, typename U >

static void copyVectors( std::vector<T> * vectorOne, std::vector<U> * vectorTwo ){

//cout<<"in copy vectors\n";

vectorTwo->clear();

typename vector<T>::iterator it;

for( it = vectorOne->begin(); it != vectorOne->end(); it ++ ){

T element = (*it);

vectorTwo->push_back( element );

}

//cout<<"Done\n";

}//end template function

/**

* Checks if the input vector of type T contains the element

* of type U.

*

* @param vectorIn - vector of interest

* @param val - value to be searched

*

* @return - true/false for search outcome

*/

template< typename T, typename U >

static bool vector_contains( std::vector<T> * vectorIn, U val ){

typename vector<T>::iterator it;

for( it = vectorIn.begin(); it != vectorIn->end(); it ++ ){

U element = (*it);

if( element == val ){

return true;

}//end if

}//end for

return false;

}//end template function

/**

* Checks whether the input map contains the input element.

*

* @param mapIn - map to search in

* @param element - element to search for

*

* @return - true/false for presence of element in map

*/

template< typename T, typename U, typename V >

static bool map_contains( std::map<T, U> & mapIn, V element ){

typename map<T, U>::iterator it;

for( it = mapIn.begin(); it != mapIn.end(); it ++ ){

if( (*it).first == element ){

return true;

}

}

return false;

}

/**

* Checks whether the input map contains the input varable,

* after multiplying both by a modifier.

*

* for use when the keys and values and the variable to search

* are both doubles, and so multiplying by an integer avoids

* floating point problems

*

* @param mapIn - map to search in

* @param element - element to search for

*

* @return - true/false for presence of element in map

*/

static bool map_contains_with_modifier( std::map<double, double> mapIn, double element, int modifier ){

std::map<double, double>::iterator it;

for( it = mapIn.begin(); it != mapIn.end(); it ++ ){

double val = (*it).first;

if( (int)( val * modifier ) == (int)( element * modifier ) ){

std::cout<<"";

return true;

}

}

return false;

}

template< typename T, typename U, typename V >

static void copy_and_add_with_modifier( std::vector<T> * variable_array, std::vector<U> * K_array, V modifier ){

//recall that the vector stored in layer_temp_variable_map.at( layer ) is the

//same length of the vector stored in k1[layerNo], as the elements are both the

//variables of our leaf layer, currently being solved.

for( unsigned int i = 0; i < K_array->size(); i ++ ){

U k_value = K_array->at( i );

//cout<<"previous value = "<<variable_array->at( i )<<"\n";

//double val = (modifier * k_value);

//cout<<"val to add = "<<val<<"\n";

variable_array->at( i ) += (modifier * k_value);

//cout<<"New value = "<<variable_array->at( i )<<"\n";

}

}

template< typename T, typename U >

static void add_val_to_elems( std::vector<T>, U val_to_add ){

typename vector<T>::iterator it;

for( it = vectorIn.begin(); it != vectorIn->end(); it ++ ){

U element = (*it);

element += val_to_add;

}//end for

}//end function//*/

/**

* adds the contents of our input vectors to their corresponding primitive arrays.

* used for compatability with NAG (which takes only primitive arrays).

*

* @param (HAD_ar[]) - reference to HAD array, for integrating by NAG

* @param (TOT_ar[]) - reference to total leaf area array, for integrating by NAG

* @param HadVec - vector containing HAD points for leaf layer, to be added to HAD array

* @param totVec - vector containing total area points for leaf layer, to be added to tot area array

*/

template< typename T, typename U, typename V, typename X>

void add_to_arrays( T ar1[], U ar2[], std::vector<V>* vec1, std::vector<X>* vec2 ){

for( unsigned int i = 0; i < vec1->size(); i ++ ){

ar1[i] += vec1->at( i );

}

for( unsigned int i = 0; i < vec2->size(); i ++ ){

ar2[i] += vec2->at( i );

}

}//*/

template< typename T, typename U >

static void convert_vec_to_array( T arr[], std::vector<U> & vec ){

for( unsigned int i = 0; i < vec.size(); i ++ ){

arr[i] = vec.at( i );

}

}

template< typename T, typename U >

static void convert_vec_to_array( T arr[], std::vector<U> * vec ){

for( unsigned int i = 0; i < vec.size(); i ++ ){

arr[i] = vec.at( i );

}

}

#endif

**leafLayer.cpp**

/**

* File: leafLayer.cpp

* Author: James Kitchen

*

* Created on June 6, 2013

*/

#include "leafLayer.h"

#include "JKUtils.h"

#include "parameters.h"

using namespace std;

/*

* define/initialise static variables

*/

double leafLayer::totalArea = 0.0;

double leafLayer::totalHealthyArea = 0.0;

double leafLayer::totalInfectiousArea = 0.0;

double leafLayer::totalLatentArea = 0.0;

double leafLayer::totalExtension = 0.0;

double leafLayer::totalSenescenceDiff = 0.0;

/**

* layer constructor

*

* @param layerNoIn - leaf layer number (1-15)

* @param A_maxIn - maximum GAI for leaf

* @param t_emergeIn - time of layer emergence

* @param t_lagIn - length of lag period

* @param t_senesIn - time of onset of senescence

* @param t_death - time of death

*

*/

leafLayer::leafLayer( unsigned short int layerNoIn, double A_maxIn, double t_iniIn,

double t_emergeIn, double t_senesIn, double t_deathIn ){

//initialise our object variables to the input values

this->layerNo = layerNoIn;

this->A_max = A_maxIn;

this->t_initiation = t_iniIn;

this->t_emerge = t_emergeIn;

this->t_senes = t_senesIn;

this->t_death = t_deathIn;

//initialise all other variables to zero

this->timeInSenescence = 0.0;

this->A_i = 0.0;

this->healthy_area = 0.0;

this->sensitive_infectious_area = 0.0;

this->resistant_infectious_area = 0.0;

this->high_risk_seed_treat_amount = 0.0;

this->low_risk_seed_treat_amount = 0.0;

//and all booleans to false

this->layerActive = false;

this->is_growing = false;

this->is_extending = false;

this->is_senescing = false;

//initialise our latent area arrays according to the number of latent compartments

sensitive_latent_area = new vector<double>();

resistant_latent_area = new vector<double>();

cout<<"no_of_latent_compartments = "<<parameters::no_of_latent_compartments<<"\n";

for( unsigned int i = 0; i < parameters::no_of_latent_compartments; i ++ ){

sensitive_latent_area->push_back( 0.0 );

resistant_latent_area->push_back( 0.0 );

}//end for

high_risk_foliar_dose_at_sprays = new map<double, double>();

low_risk_foliar_dose_at_sprays = new map<double, double>();

cout<<layerNo<<" sensitive_latent_area = "<<sensitive_latent_area<<"\n";

cout<<"Leaf layer "<<layerNo<<" created, A_max = "<<A_max<<"\n";

}//end constructor

/**

* zeroes the layer, for in between growing seasons

*/

void leafLayer::zeroLayer(){

//Zero epidemiological variables

this->A_i = 0.0;

this->healthy_area = 0.0;

this->sensitive_infectious_area = 0.0;

this->resistant_infectious_area = 0.0;

this->high_risk_seed_treat_amount = 0.0;

this->low_risk_seed_treat_amount = 0.0;

vector<double>::iterator it;

for( it = sensitive_latent_area->begin(); it != sensitive_latent_area->end(); it ++ ){ (*it) = 0.0;}

for( it = resistant_latent_area->begin(); it != resistant_latent_area->end(); it ++ ){ (*it) = 0.0;}

//also zero the amount of fungicide in the leaf!

high_risk_foliar_dose_at_sprays->clear();

low_risk_foliar_dose_at_sprays->clear();

//and return life cycle booleans to false

this->layerActive = false;

this->is_growing = false;

this->is_extending = false;

this->is_senescing = false;

}//end function

/**

* loops through our arrays for sensitive and resistant area and sums them.

* @return - the total sensitive and resistant latent compartment area index.

*/

double leafLayer::getTotalLatentArea(){

double total_compartment_area = 0.0;

vector<double>::iterator it;

for( it = sensitive_latent_area->begin(); it != sensitive_latent_area->end(); it ++ ){

total_compartment_area += (*it);

}

for( it = resistant_latent_area->begin(); it != resistant_latent_area->end(); it ++ ){

total_compartment_area += (*it);

}

return total_compartment_area;

}

/**

* Calculates the height of this leaf due to stem extension

* as a linear function of time, simply rate x time.

* if the extension exceeds the maximum extension, the maximum

* extension is returned.

*

* @param t - current time value

* @return - calculated extension, or the maximum extension

* if exceeded by the calculated value.

*/

double leafLayer::calc_extension( double t ){

double ex = 0.0;

if( this->isExtending() ){

double time_extended = t - this->t_emerge;

ex = parameters::ex_rate * time_extended;

//check if we have surpassed the maximum amount of extension,

//in which case we simply return this value

if( ex > parameters::maximum_extension ){ex = parameters::maximum_extension;}

}//end if

return ex;

}

/**

*

* calculates the derivative of the differential equation:

*

* da/dt = gamma * (A_max - A_i ) - providing a monomolecular

* growth function

* gamma = growth rate = 0.034

* A_max = maximum area for leaf layer

* A_i = current leaf area

*

* @param t - time step

* @param y - the current dependent variable of the function

*

* @return - double variable representing the calculated derivative

*

*/

double leafLayer::calc_Ai_deriv( double t, double leaf_area ){

if( t < this->get_t_ini() ){

return 0.0;

}

if( t == this->t_death ){

return 0.0;

}

/**

* if the plant is growing we use the first,

* monomolecular growth function. This will

* automatically lead to a lag when A_i = A_max

*/

else if( this->isGrowing() ){

double dadt = parameters::growthRate * ( A_max - leaf_area );

return dadt;

/**

* Once senescence kicks in, we switch functions to

* incorporate the logistic decay function

*/

}else if( this->isSenescing() ){

/**

* the below code was used for calculating 'A' in the senescence function, e.g.,

* to help fit our time in senescence (area above a threshold of 0.01) better to

* the amount of time used for senescence.

*/

if( this->healthy_area > 0.01 && fmod( t, 1 ) ){

this->timeInSenescence ++;

}//*/

return 0.0;

}else{

//leaf must be dead

return 0.0;

}// */

}//end function

/**

* Takes in the input time value and checks through

* all phenology parameters, to determine if the leaf

* must switch to another life cycle stage. It then

* sets the corresponding boolean values.

* @param t - current time value

*/

void leafLayer::check_life_cycle_stage( double t, double & sum_sensitive, double & sum_resistant,

unsigned int max_l_no_resist_calc ){

if( ( t >= this->t_initiation && t < this->t_emerge ) && !this->isActive() ){

cout<<"leaf "<<this->layerNo<<" active at time "<<t<<"\n";

this->set_Active( true );

this->set_growing( true );

}else if( ( t >= this->t_emerge && t < this->t_senes ) && !this->isExtending() ){

this->set_extending( true );

}else if( ( t >= this->t_senes && t < this->t_death ) && !this->isSenescing() ){

this->set_senescing( true );

this->set_growing( false );

}else if( t >= this->t_death && this->isActive() ){

if( this->getLayerNo() <= max_l_no_resist_calc ){

sum_sensitive += this->getSensitiveInfectiousArea();

sum_resistant += this->getResistantInfectiousArea();

}

this->zeroLayer();

this->set_Active( false );

}//end else if

}//end function

/**

* Provides the logistic rate function for senescence.

*

* If t < t_senes returns 0, otherwise we return a

* function of exponential decay to be later integrated.

*

* @param t - current time step

* @return - rate value of logistic decay / 0.0

* if t < t_senes

*/

double leafLayer::senes_eq( double t ){

//calculate the exponential term first, known as eVal

double eVal = 0.0;

if( this->isSenescing() ){

eVal = parameters::a*exp( parameters::s_rate*( t-t_death ) );

}

return eVal;

}

/**

* Reads the parameter files for leaf layer data, such as

* layer growth initiation, senescence initiation and death.

* constructs the leaf layer objects

*

* @return - linked list of the newly generated layer objects

*/

list<leafLayer*>* leafLayer::makeLeafLayers(){

cout<<"Reading in leaf layer parameters\n";

list<leafLayer*> * layers = new list<leafLayer*>();

//string leaf_file = "D:/seed_treatment_sims/i_files/ST_in_l_11.txt";

string leaf_file = "ST_in.txt";

string line;

ifstream myfile ( leaf_file );

int layerNo = 0;

double A_max = 0.0;

double t_init = 0.0;

double t_emerge = 0.0;

double t_senes = 0.0;

double t_death = 0.0;

if( leaf_file != "ST_in.txt" ){

string ans;

cout<<"Non standard leaf layer parameters used. Are you happy? y\\n\n";

cin>>ans;

if( ans == "n" ){exit( 0 );}

}

if( myfile.is_open() ){

while ( myfile.good() ){

/*LEAF_LAYER_BEGIN

NUMBER 1

AMAX 0.96

GROWTH_INI 1635

LAG_INI 1700

SENES_INI 2695.06

DEATH 2914.66

END_LAYER*/

getline( myfile, line );

//split up according to single white spaces:

vector<string> out = split( line, ' ' );

string word_one;

/*

* if we hit an empty line, an exception will be thrown.

* we ignore it and continue (it will still throw an exception

* in debug mode of visual c++ apparently).

*/

try{

word_one = out.at( 0 );

}catch( out_of_range &e ){

//cout<<" exception "<<e.what()<<" made\n";

continue;

}//end catch

if( word_one == "NUMBER" ){

layerNo = atoi( out.at( 1 ).c_str() );

cout<<" layerNo = "<<layerNo<<"\n";

}//*/

if( word_one == "AMAX" ){

A_max = atof( out.at( 1 ).c_str() );

cout<<" A_max = "<<A_max<<"\n";

}//*/

if( word_one == "GROWTH_INI" ){

t_init = atof( out.at( 1 ).c_str() );

cout<<"t_initiation = "<<t_init<<"\n";

}//*/

if( word_one == "EMERGENCE" ){

t_emerge = atof( out.at( 1 ).c_str() );

cout<<"t_emergence = "<<t_emerge<<"\n";

}//*/

if( word_one == "SENES_INI" ){

t_senes = atof( out.at( 1 ).c_str() );

cout<<"t_senes = "<<t_senes<<"\n";

}//*/

if( word_one == "DEATH" ){

t_death = atof( out.at( 1 ).c_str() );

cout<<"t_death = "<<t_death<<"\n";

}//*/

if( word_one == "END_LAYER" ){

cout<<"Making new layer object\n";

//now we generate the leaf layer object

leafLayer * layer = new leafLayer( layerNo, A_max, t_init, t_emerge, t_senes, t_death );

cout<<"New layer "<<layer->layerNo<<" created\n";

layers->push_back( layer );

cout<<"layer added to list (size: "<<layers->size()<<") \n";

}//end if

}//end while

myfile.close();

}else{

cout << "Unable to open layer parameter file";

exit( 1 );

}

cout<<"layer size in function = "<<layers->size()<<"\n";

cout<<"Finished Reading in leaf layer parameters\n";

myfile.close();

return layers;

}//end function

/**

* Reads the parameter files for leaf layer data, such as

* layer growth initiation, senescence initiation and death.

* constructs the leaf layer objects

*

* @return - linked list of the newly generated layer objects

*/

list<leafLayer*>* leafLayer::makeLeafLayers( string & fileName ){

cout<<"Reading in leaf layer parameters\n";

list<leafLayer*> * layers = new list<leafLayer*>();

string line;

ifstream myfile ( fileName );

int layerNo = 0;

double A_max = 0.0;

double t_init = 0.0;

double t_emerge = 0.0;

double t_senes = 0.0;

double t_death = 0.0;

if( myfile.is_open() ){

while ( myfile.good() ){

/*LEAF_LAYER_BEGIN

NUMBER 1

AMAX 0.96

GROWTH_INI 1635

LAG_INI 1700

SENES_INI 2695.06

DEATH 2914.66

END_LAYER*/

getline( myfile, line );

//split up according to single white spaces:

vector<string> out = split( line, ' ' );

string word_one;

/*

* if we hit an empty line, an exception will be thrown.

* we ignore it and continue (it will still throw an exception

* in debug mode of visual c++ apparently).

*/

try{

word_one = out.at( 0 );

}catch( out_of_range &e ){

//cout<<" exception "<<e.what()<<" made\n";

continue;

}//end catch

if( word_one == "NUMBER" ){

layerNo = atoi( out.at( 1 ).c_str() );

cout<<" layerNo = "<<layerNo<<"\n";

}//*/

if( word_one == "AMAX" ){

A_max = atof( out.at( 1 ).c_str() );

cout<<" A_max = "<<A_max<<"\n";

}//*/

if( word_one == "GROWTH_INI" ){

t_init = atof( out.at( 1 ).c_str() );

cout<<"t_initiation = "<<t_init<<"\n";

}//*/

if( word_one == "EMERGENCE" ){

t_emerge = atof( out.at( 1 ).c_str() );

cout<<"t_emergence = "<<t_emerge<<"\n";

}//*/

if( word_one == "SENES_INI" ){

t_senes = atof( out.at( 1 ).c_str() );

cout<<"t_senes = "<<t_senes<<"\n";

}//*/

if( word_one == "DEATH" ){

t_death = atof( out.at( 1 ).c_str() );

cout<<"t_death = "<<t_death<<"\n";

}//*/

if( word_one == "END_LAYER" ){

cout<<"Making new layer object\n";

//now we generate the leaf layer object

leafLayer * layer = new leafLayer( layerNo, A_max, t_init, t_emerge, t_senes, t_death );

cout<<"New layer "<<layer->layerNo<<" created\n";

layers->push_back( layer );

cout<<"layer added to list (size: "<<layers->size()<<") \n";

}//end if

}//end while

myfile.close();

}else{

cout << "Unable to open layer parameter file";

exit( 1 );

}

cout<<"layer size in function = "<<layers->size()<<"\n";

cout<<"Finished Reading in leaf layer parameters\n";

myfile.close();

return layers;

}//end function

/**

* averages the total difference between the expected time

* in senescence with the observed. Averages over 11 leaf

* layers.

*

* @param file - reference of filestream object to

* write to.

*

*/

void leafLayer::avg_senes_disparity( fstream & file ){

double avg = totalSenescenceDiff / 11;

if( file.is_open() ){

file<<" a = average senescence difference = "<<avg<<"\n";

}

}//end method

/**

* sums up the static variables for total area, healthy area, and latent area.

* These must be zeroed between growing seasons!

*

*/

void leafLayer::sum_total_variables( list<leafLayer*> * layers ){

leafLayer::totalArea = 0.0;

leafLayer::totalHealthyArea = 0.0;

leafLayer::totalLatentArea = 0.0;

leafLayer::totalInfectiousArea = 0.0;

list<leafLayer*>::iterator it;

for( it = layers->begin(); it != layers->end(); it ++ ){

leafLayer * layer = (*it);

//only sum for the top three leaf layers

if( layer->getLayerNo() <= 3){

leafLayer::totalInfectiousArea += layer->getSensitiveInfectiousArea() + layer->getResistantInfectiousArea();

leafLayer::totalArea += layer->getArea();

leafLayer::totalHealthyArea += layer->get_healthy_area();

leafLayer::totalLatentArea += layer->getTotalLatentArea();

}//end if

}//end for

}//end function

/**

* prints the total area of each leaf layer to the input file

*

* @param layers - list of layer pointers

* @param outfile - reference to file stream file object

*

*/

void leafLayer::total_growth( list<leafLayer*> * layers, fstream & outfile ){

double totalArea = 0.0;

list<leafLayer*>::iterator it;

for( it = layers->begin(); it != layers->end(); it ++ ){

leafLayer * currentLayer = (*it);

totalArea += currentLayer->getArea();

}

outfile<<"Total area = "<<totalArea<<"\n";

}

/**

* prints the healthy, latent s and r and infectious s and r densities for

* this leaf layer.

*

* @param t - current time step

* @param outFile - reference to our file object

*/

void leafLayer::write_densities( double t, fstream & outFile ){

outFile<<"\n----------\ntime: "<<t<<"\n";

outFile<<"layer "<<this->getLayerNo()<<":\n----------\n";

outFile<<"Area = "<<this->getArea()<<"\n";

outFile<<"Healthy area = "<<this->get_healthy_area()<<"\n";

for( unsigned int i = 0; i < this->sensitive_latent_area->size(); i ++ ){

double area = sensitive_latent_area->at( i );

outFile<<"Sensitive latent compartment "<<i<<": area = "<<area<<"\n";

}//end for

for( unsigned int i = 0; i < this->resistant_latent_area->size(); i ++ ){

double area = resistant_latent_area->at( i );

outFile<<"Resistant latent compartment "<<i<<": area = "<<area<<"\n";

}//end for

//outFile<<"Severity = "<<this->calc_disease_severity()<<"\n";

outFile<<"Sensitive infectious area = "<<this->getSensitiveInfectiousArea()<<"\n";

outFile<<"Resistant infectious area = "<<this->getResistantInfectiousArea()<<"\n";

outFile<<"End layer "<<this->getLayerNo()<<":\n----------"<<endl;

}//*/

/**

* Writes out the column names for the function write_densities_column

*

* @param outFile - file stream to write to

*/

void leafLayer::write_densities_column_names( fstream & outFile ){

outFile<<"time layer_no area healthy_area ";

for( int i = 1; i <= 10; i ++ ){outFile<<"sensitive_latent_"<<i<<" ";}

for( int i = 1; i <= 10; i ++ ){outFile<<"resistant_latent_"<<i<<" ";}

outFile<<"sensitive_infectious resistant_infectious high_risk_ST low_risk_ST year\n";

}

/**

* prints the healthy, latent s and r and infectious s and r densities for

* this leaf layer.

*

* @param t - current time step

* @param outFile - reference to our file object

*/

void leafLayer::write_densities_column( double t, fstream & outFile, int year ){

outFile<<t<<" ";

outFile<<this->getLayerNo()<<" ";

outFile<<this->getArea()<<" ";

outFile<<this->get_healthy_area()<<" ";

for( unsigned int i = 0; i < this->sensitive_latent_area->size(); i ++ ){

double area = sensitive_latent_area->at( i );

outFile<<area<<" ";

}//end for

for( unsigned int i = 0; i < this->resistant_latent_area->size(); i ++ ){

double area = resistant_latent_area->at( i );

outFile<<area<<" ";

}//end for

//outFile<<"Severity = "<<this->calc_disease_severity()<<"\n";

outFile<<this->getSensitiveInfectiousArea()<<" ";

outFile<<this->getResistantInfectiousArea()<<" ";

outFile<<this->get_high_risk_seed_treat_amount()<<" ";

outFile<<this->get_low_risk_seed_treat_amount()<<" ";

outFile<<year<<endl;

}//*/

/*void leafLayer::write_densities( double t, fstream & outFile ){

outFile<<"Time "<<t<<" Leaf "<<this->getLayerNo()<<" Area "<<this->getArea()<<"Healthy area "<<this->get_healthy_area()<<"

}//*/

/**

* Writes out leaf specific severity data to the input file stream.

*

* @param t - current time step

* @param outFile - file stream object

* @param layerNo - the leaf layer number for which we want severities being

* written out

*/

void leafLayer::print_severity( double t, fstream & outFile, int layerNo ){

if( this->getLayerNo() == layerNo ){

outFile<<layerNo<<" "<<t<<" "<<calc_disease_severity()<<"\n";

}

}

/**

* prints the healthy, latent s and r and infectious s and r densities for

* this leaf layer.

*

* @param t - current time step

*/

void leafLayer::print_densities( double t ){

cout<<"\n----------\ntime: "<<t<<"\n";

cout<<"layer "<<this->getLayerNo()<<":\n----------\n";

cout<<"Area = "<<this->getArea()<<"\n";

cout<<"Healthy area = "<<this->get_healthy_area()<<"\n";

for( unsigned int i = 0; i < this->sensitive_latent_area->size(); i ++ ){

double area = sensitive_latent_area->at( i );

cout<<"Sensitive latent compartment "<<i<<": area = "<<area<<"\n";

}//end for

for( unsigned int i = 0; i < this->resistant_latent_area->size(); i ++ ){

double area = resistant_latent_area->at( i );

cout<<"Resistant latent compartment "<<i<<": area = "<<area<<"\n";

}//end for

cout<<"Sensitive infectious area = "<<this->getSensitiveInfectiousArea()<<"\n";

cout<<"Resistant infectious area = "<<this->getResistantInfectiousArea()<<"\n";

cout<<"Severity = "<<this->calc_disease_severity()<<"\n";

cout<<"End layer "<<this->getLayerNo()<<":\n----------\n";

}//end method

/**

* calculates the percentage of leaf layer area

* that has belongs to infectious area

*/

double leafLayer::calc_disease_severity(){

return( ( this->sensitive_infectious_area + this->resistant_infectious_area )/this->A_i * 100 ) ;

}

/**

* adds the new dose of high risk fungicide on spraying to the dose already present on the leaf.

*

* The amount being sprayed will be in mg / M squared, but the amount stored in the leaf

* layer will be mg / M cubed, so we must first convert back. When decay is calculated

* a dose per unit volume will be calculated.

*

* @param new_dose - dose to add

* @param t_spray - time of spraying

*/

void leafLayer::add_high_risk_foliar_dose( double new_dose, double t_spray ){

this->high_risk_foliar_dose_at_sprays->insert( make_pair( t_spray, new_dose ) );

}

/**

* adds the new dose of high risk fungicide on spraying to the dose already present on the leaf.

*

* The amount being sprayed will be in mg / M squared, but the amount stored in the leaf

* layer will be mg / M cubed, so we must first convert back. When decay is calculated

* a dose per unit volume will be calculated.

*

* @param new_dose - dose to add

* @param t_spray - time of spraying

*/

void leafLayer::add_low_risk_foliar_dose( double new_dose, double t_spray ){

this->low_risk_foliar_dose_at_sprays->insert( make_pair( t_spray, new_dose ) );

}

/**

* Static method for printing seed treatment fungicide concentration per leaf, requires

* the concentration to be input.

*

* @param layer - current leaf layer

* @param t - current time step

* @param conc - current seed treatment fungicide concentration

* @param area - area of leaf, not to be confused with healthy area, as this is used for the calculation

*/

void leafLayer::print_layer_foliar_conc( leafLayer * layer, double t, double conc, double area ){

fstream ofile;

ofile.open( "foliar_conc_per_leaf.txt", ios::out | ios::app );

ofile<<"Time = "<<t<<" layer number = "<<layer->getLayerNo()<<" foliar fungicide concentration = "<<conc<<" leaf area = "<<area<<"\n";

ofile.close();

}

/**

* Static method for printing seed treatment fungicide concentration per leaf, requires

* the concentration to be input.

*

* @param layer - current leaf layer

* @param t - current time step

* @param conc - current seed treatment fungicide concentration

* @param N_layer - amount of seed treatment fungicide in leaf, in mg

* @param healthyArea - healthy area of leaf, as this is used for the calculation

*/

void leafLayer::print_layer_seed_treatment_conc( leafLayer * layer, double t, double N_layer, double conc, double healthyArea ){

fstream ofile;

ofile.open( "ST_conc_per_leaf.txt", ios::out | ios::app );

ofile<<"Time = "<<t<<" layer number = "<<layer->getLayerNo()<<" amount of seed treatment fungicide = "<<N_layer<<" concentration = "<<conc<<" healthy area = "<<healthyArea<<"\n";

ofile.close();

}

/**

* Static method for printing out seed treatment dose information to a file

*

* @param layers - list of leaf layer objects

* @param ofile - file stream for writing to

* @param fung_amount - amount of fungicide left in the seed reservoire

*/

void leafLayer::print_layer_seed_treatment_amount( list<leafLayer*> * layers, fstream & ofile, double t, double fung_amount ){

list<leafLayer*>::iterator it;

for( it = layers->begin(); it != layers->end(); it ++ ){

leafLayer * layer = (*it);

if( !layer->isActive() ){

continue;

}

ofile<<"Time = "<<t<<" layer number = "<<layer->getLayerNo()<<" high risk ST dose = "<<layer->get_high_risk_seed_treat_amount()<<" high risk ST dose = "<<layer->get_low_risk_seed_treat_amount()<<" seed reservoire = "<<fung_amount<<" area = "<<layer->getArea()<<"\n";

cout<<"Time = "<<t<<" layer number = "<<layer->getLayerNo()<<" high risk ST dose = "<<layer->get_high_risk_seed_treat_amount()<<" high risk ST dose = "<<layer->get_low_risk_seed_treat_amount()<<" seed reservoire = "<<fung_amount<<" area = "<<layer->getArea()<<"\n";

}

}

/**

* static method for printing out seed treatment dose information to a file

*

* @param layers - list of leaf layer objects

* @param ofile - file stream for writing to

* @param fung_amount - amount of fungicide left in the seed reservoire

*/

void leafLayer::print_total_layers_seed_treat_conc( list<leafLayer*> * layers, fstream & ofile, double t, double high_fung_amount, double low_fung_amount ){

list<leafLayer*>::iterator it;

double high_tot_conc = 0.0;

double low_tot_conc = 0.0;

for( it = layers->begin(); it != layers->end(); it ++ ){

leafLayer * layer = (*it);

double leaf_healthy_area = layer->get_healthy_area();

double h_ST_amount = layer->get_high_risk_seed_treat_amount();

double l_ST_amount = layer->get_low_risk_seed_treat_amount();

double high_conc = 0.0;

double low_conc = 0.0;

//check we don't divide by zero.

if( parameters::omega * leaf_healthy_area != 0.0 ){

high_conc = ( h_ST_amount / ( parameters::omega * leaf_healthy_area ) );

low_conc = ( l_ST_amount / ( parameters::omega * leaf_healthy_area ) );

}

high_tot_conc += high_conc;

low_tot_conc += low_conc;

}

//cout<<" Time = "<<t<<" Getting seed_treat conc\n";

ofile<<"Time = "<<t<<" amount of high risk in seed reservoire = "<<high_fung_amount<<" amount of low risk in seed "<<low_fung_amount<<" total high risk concentration "<<high_tot_conc<<" total low risk conc "<<low_tot_conc<<"\n";

}

/**

* static method for printing out seed treatment dose information to a file

*

* @param layers - list of leaf layer objects

* @param ofile - file stream for writing to

* @param fung_amount - amount of fungicide left in the seed reservoire

*/

void leafLayer::print_total_layers_seed_treat_amount( list<leafLayer*> * layers, fstream & ofile, double t, double high_fung_amount, double low_fung_amount ){

list<leafLayer*>::iterator it;

double high_tot_amount = 0.0;

double low_tot_amount = 0.0;

for( it = layers->begin(); it != layers->end(); it ++ ){

leafLayer * layer = (*it);

double leaf_area = layer->getArea();

double high_ST_amount = layer->get_high_risk_seed_treat_amount();

double low_ST_amount = layer->get_low_risk_seed_treat_amount();

high_tot_amount += high_ST_amount;

low_tot_amount += low_ST_amount;

}

ofile<<"Time = "<<t<<" high risk amount in seed reservoire = "<<high_fung_amount<<" low risk amount in seed reservoire = "<<low_fung_amount<<" total high risk concentration "<<high_tot_amount<<" total low risk concentration"<<low_tot_amount<<"\n";

}

**leafLayer.h**

/**

* File: leafLayer.h

* Author: Dr James L Kitchen

* Plant Epidemiology and evolutionary biology group

* The centre for Computational and Systems Biology

* Rothamsted Research

*

* Created on June 6, 2013

*

* Each leaf layer is explicitly simulated.

* Once a leaf has emerged, it grows according to a monomolecular function

* until it reaches its maximum potential growth. Its mass then plateaus

* for a defined amount of time until it senesces according to a logistic

* decay function.

*

* leaf layers also undergo stem extension, where a layer L will begin to extend

* once the next youngest leaf (i.e. the layer that emerged immediately after L)

* has emerged. Layers extend at a rate of 1cm per 10 degree days up until a

* maximum height of 10 cm.

*

*/

#ifndef LEAFLAYER_H

#define LEAFLAYER_H

#include <iostream>

#include <vector>

#include <math.h>

#include <list>

#include <map>

#include <istream>

#include <fstream>

#include <exception>

#include <stdexcept>

class leafLayer{

private:

/**

* Plant growth model variables

*/

unsigned short int layerNo; //layer number of 1 - 11

double A_max; //maximum green leaf area for layer

double A_i; //current green leaf area of layer

double t_initiation; //time of leaf growth initiation (all time units are in degree days)

double t_emerge; //time when the leaf has fully grown and so beings the lag period for layer, in between end of leaf growth and onset of senescence

double t_senes; //onset of senescence

double t_death; //end of senscence, as in leaf death

/**

* The below booleans are switches for different life cycle stages,

* which are set outside of our integrator to avoid boundary

* problems. They will also have to be reset to false once a leaf dies

*/

bool layerActive; //true/false for whether the leaf has started growing. It will

//have an effect on inoculum transfer and spraying of other leaves.

bool is_growing; //true/false for whether the leaf has started growing. It will

//have an effect on inoculum transfer and spraying of other leaves.

bool is_extending; //true/false for whether the leaf has started extending. It will

//have an effect on inoculum transfer between leaves

bool is_senescing; //true/false for whether the leaf has started senescing

/**

* Plant disease model variables

*/

double healthy_area; //the area that is not occupied by any latent or infectious lesions

double sensitive_infectious_area; //the area that is infected with infectious lesions belonging to the sensitive strain

double resistant_infectious_area; //the area that is infected with infectious lesions belonging to the resistant strain

std::vector<double>* sensitive_latent_area; //an array containing all n latent compartments, of the latent area belonging to the sensitive strain

std::vector<double>* resistant_latent_area; //an array containing all n latent compartments, of the latent area belonging to the resistant strain

double timeInSenescence; //for debugging, the amount of time that the GAI of a leaf layer is greater than 0.01 (h_death)

/**

* fungicide model variables

*/

std::map<double, double>* high_risk_foliar_dose_at_sprays; //the concentration of high risk foliar spray fungicide that was reached by the leaf layer at the time of spraying

std::map<double, double>* low_risk_foliar_dose_at_sprays; //the concentration of low risk foliar spray fungicide that was reached by the leaf layer at the time of spraying

double high_risk_seed_treat_amount; //the amount in mg of product per milligrams of seed of high risk fungicide dose from seed treatment presently on the leaf layer

double low_risk_seed_treat_amount; //the amount in mg of product per milligrams of seed of low risk fungicide dose from seed treatment presently on the leaf layer

public:

//static variables

static double totalExtension; //for debugging, accumulates the total layer extension

static double totalArea; //for debugging and HAD calculation, accumulates the total layer area

static double totalHealthyArea; //for debugging and HAD calculation, accumulates the total layer healthy area

static double totalInfectiousArea; //for debugging and HAD calculation, accumulates the total layer healthy area

static double totalLatentArea; //for HAD calculation, sums up the latent density on all leaf layers

static double totalSenescenceDiff; //for debugging, totals the discrepancy in time in senescence

//with a GAI < 0.01, and the value of t_death-t_senescence for each

//leaf layer

/**

* static functions

*/

static std::list<leafLayer*>* makeLeafLayers(); //generates leaf layers from parsing input parameter file

static std::list<leafLayer*>* makeLeafLayers( std::string & fileName ); //generates leaf layers from parsing input parameter file

static void total_growth( std::list<leafLayer*>* layers, //prints the summed area for all leaf layers when called

std::fstream & outfile );

static void avg_senes_disparity( std::fstream & file ); //averages the total difference between the expected time

//in senescence with the observed. Averages over 11 leaf

//layers.

static void sum_total_variables( std::list<leafLayer*> * layers ); //sums total area, healthy area, latent area and stem extension

static void print_layer_seed_treatment_amount( std::list<leafLayer*> * layers, //prints the dose of the fungicide that came from the seed treatment

std::fstream & ofile, double t,

double fung_amount );

static void print_layer_seed_treatment_conc( leafLayer * layer_i, //prints the dose of the fungicide that came from the seed treatment

double t, double N_layer,

double concen, double healthy_area ); //this one uses the precalculated version of the concentration (does not)

//calculate it itself.

static void print_layer_foliar_conc( leafLayer * layer, double t, //prints the dose of the fungicide that came from the foliar spray

double conc, double area );

static void print_layer_seed_treatment_conc( std::list<leafLayer*> * layers, //prints the concentration of the fungicide that came from the seed treatment

std::fstream & ofile, double t,

double fung_amount );

static void print_total_layers_seed_treat_amount( std::list<leafLayer*> * layers,

std::fstream & ofile, double t,

double high_fung_amount,

double low_fung_amount );

static void print_total_layers_seed_treat_conc( std::list<leafLayer*> * layers,

std::fstream & ofile, double t,

double high_fung_amount,

double low_fung_amount );

/*

* CONSTRUCTORS

*/

leafLayer();

// Layer constructor

leafLayer( unsigned short int layerNoIn, double A_maxIn, double t_iniIn,

double t_emergeIn, double t_senesIn, double t_deathIn );

// Copy constructor

leafLayer( leafLayer & orig ){

std::cout<<"Copy constructor for layer "<<orig.layerNo<<" called!\n";

}

// Destructor

~leafLayer(){

std::cout<<"Deleting layer number "<<this->layerNo<<"\n";

}

/**

* Setters

*/

/**

* sets the current green leaf area

*

* @param areaIn - new area

*

*/

void setArea( double areaIn ){

A_i = areaIn;

}

/**

* sets the current green leaf area

*

* @param areaIn - new area

*

*/

void setHealthyArea( double hIn ){

healthy_area = hIn;

}

void setLatent_sensitive( std::vector<double> * val ){

this->sensitive_latent_area = val;

}

void setLatent_resistant( std::vector<double> * val ){

this->resistant_latent_area = val;

}

void setInfectious_sensitive( double val ){

this->sensitive_infectious_area = val;

}

void setInfectious_resistant( double val ){

this->resistant_infectious_area = val;

}

void set_high_risk_seed_treat_amount( double val ){

high_risk_seed_treat_amount = val;

}

void set_low_risk_seed_treat_amount( double val ){

low_risk_seed_treat_amount = val;

}

/**

* Sets growth initiation to true/false

* @param val - new true/false to be applied

*/

void set_Active( bool val ){

this->layerActive = val;

}

/**

* Sets growth initiation to true/false

* @param val - new true/false to be applied

*/

void set_growing( bool val ){

this->is_growing = val;

}

/**

* Sets leaf extension to true/false, according to

* leaf number

* @param val - new true/false to be applied,

* provided leaf number is correct

*/

void set_extending( bool val ){

if( val == true && ( this->layerNo >= 1 && this->layerNo < 5 ) ){

this->is_extending = true;

}else{

this->is_extending = false;

}

}

/**

* Sets senescence to true/false

* @param val - new true/false to be applied

*/

void set_senescing( bool val ){

this->is_senescing = val;

}

/**

* Getters

*/

/**

* get time of layer emergence

*

* @return - time of layer emergence

*/

double get_t_ini(){

return this->t_initiation;

}//

/**

* get time of full leaf emergence, when the leaf starts extending

* (leaves 1 - 4 only).

*

* @return - time of layer lag

*/

double get_t_emerge(){

return this->t_emerge;

}//

/**

* get time of layer senescence

*

* @return - time of layer senescence

*/

double get_t_senes(){

return this->t_senes;

}//

/**

* get time of layer death

*

* @return - time of layer death

*/

double get_t_death(){

return this->t_death;

}//

/**

* obtain current leaf area

*

* @return - current area index of layer

*/

double getArea(){

return A_i;

}

/**

* obtain current leaf area

*

* @return - current area index of layer

*/

double get_Amax(){

return this->A_max;

}

/**

* returns leaf layer number (1-15)

*

* @return - layer number

*

*/

unsigned short int getLayerNo(){

return this->layerNo;

}

/**

* returns the sensitive infectious area within

* this leaf layer

* @return - double of the GAI given to

* infectious lesions of the

* sensitive strain

*/

double getSensitiveInfectiousArea(){

return this->sensitive_infectious_area;

}

/**

* returns the resistant infectious area within

* this leaf layer

* @return - double of the GAI given to

* infectious lesions of the

* resistant strain

*/

double getResistantInfectiousArea(){

return this->resistant_infectious_area;

}

/**

* returns an array of the area belonging to

* the latent compartments infected with sensitive

* strain

*

* @return - vector of doubles of the

* sensitive latent compartments

*/

std::vector<double>* getSensitiveLatentCompartments(){

return this->sensitive_latent_area;

}

/**

* returns an array of the area belonging to

* the latent compartments infected with resistant

* strain

*

* @return - vector of doubles of the

* resistant latent compartments

*/

std::vector<double>* getResistantLatentCompartments(){

return this->resistant_latent_area;

}

double getTotalLatentArea();

double get_healthy_area(){

return this->healthy_area;

}

/**

* returns the dose of high risk fungicide at the time of

* spraying, not the dose that has been decaying

* although it is used for calculating decay

*

* @return - foliar spray fungicide dose

*/

std::map<double,double>* get_high_risk_foliar_dose_at_sprays(){

return high_risk_foliar_dose_at_sprays;

}

/**

* returns the dose of high risk fungicide at the time of

* spraying, not the dose that has been decaying

* although it is used for calculating decay

*

* @return - foliar spray fungicide dose

*/

std::map<double,double>* get_low_risk_foliar_dose_at_sprays(){

return low_risk_foliar_dose_at_sprays;

}

/**

* returns the amount of fungicide according to

* the seed treatment

*

* @return - seed treatment fungicide dose

*/

double get_high_risk_seed_treat_amount(){

return this->high_risk_seed_treat_amount;

}

/**

* returns the amount of fungicide according to

* the seed treatment

*

* @return - seed treatment fungicide dose

*/

double get_low_risk_seed_treat_amount(){

return this->low_risk_seed_treat_amount;

}

/**

* Returns the boolean for whether

* leaf has started growing

*

* @return true/false for life cycle stage

*/

bool isGrowing(){

return is_growing;

}

/**

* Returns the boolean for whether

* leaf has started growing

*

* @return true/false for life cycle stage

*/

bool isSenescing(){

return is_senescing;

}

/**

* Returns the boolean for whether

* leaf has started growing

*

* @return true/false for life cycle stage

*/

bool isExtending(){

return is_extending;

}

/**

* Returns the boolean for whether

* leaf is active

*

* @return true/false for life cycle stage

*/

bool isActive(){

return this->layerActive;

}

/**

* calculates the probability of inoculum

* spread between this leaf layer, i, and the

* input leaf layer, j's height

*

*

* @param layerNo - layer no.

* @param heightIn - height of input leaf

*/

double getTransmissionProb( unsigned short int layerNo, double heightIn );

/**

* zeroes the layer, for in between growing seasons

*/

void zeroLayer();

/*

* Integrator for the growth differential equations,

* using the Runge Kutta solver

*/

void calc_growth_Eq( double t, double h );

double calc_Ai_deriv( double t, double area ); //Differential equations for growth

double senes_eq( double t ); //logistic rate function for decay of

//healthy area according to senescence

double calc_disease_severity(); //calculates the percentage of leaf layer area

//that has belongs to infectious area

void add_high_risk_foliar_dose( double new_dose, double t_spray ); //adds the new sprayed dose to the dose remaining in the leaf layer

void add_low_risk_foliar_dose( double new_dose, double t_spray ); //adds the new sprayed dose to the dose remaining in the leaf layer

void print_densities( double t ); //prints our healthy, latent and infectious densities

void write_densities( double t, std::fstream & outFile ); //writes the above output to a file

std::vector<double>* get_HAD_y_vals();

std::vector<double>* get_totArea_y_vals();

void update_layer_HAD( double t );

void print_severity( double t, std::fstream & outFile, //write disease severity out to a file

int layerNo );

double calc_extension( double t ); //calculates leaf extension/height as a function of time

void check_life_cycle_stage( double t, //checks the current time to switch between life cycle stages

double & sum_sensitive,

double & sum_resistant,

unsigned int max_l_no_resist_calc );

void write_densities_column( double t, std::fstream & outFile, int year );

static void write_densities_column_names( std::fstream & outFile );

};

#endif

**Main.cpp**

#include "leafLayer.h"

#include "epidemic_st.h"

#include "JKUtils.h"

#include "fungicide_st.h"

#include "parameters.h"

#include <vector>

#include <math.h>

#include <string>

using namespace std;

////////////////////////////////////////////////////////////////////////////

// The below values are for the NAG HAD integration

////////////////////////////////////////////////////////////////////////////

double ans = 0.0;

double er = 0.0;

int n = 0;

int audpc1_n = 0;

int audpc2_n = 0;//*/

////////////////////////////////////////////////////////////////////////////

// Constants for HAD duration integration.

// HAD runs from anthesis to harvest. The below quantities are in degree

// days

////////////////////////////////////////////////////////////////////////////

const unsigned int anthesis = 2100; //anthesis/flowering time

const unsigned int harvest = 3100; //harvest time

//const double max_area_under_HAD = 1842.75; //the maximum HAD area, calculated in the absence of disease

const double max_area_under_HAD = 1843.75; //the maximum HAD area, calculated in the absence of disease*/

//*/

//const double max_area_under_HAD = 1842.61; //the maximum HAD area, calculated in the absence of disease*/

//const double max_area_under_HAD = 5671.79;

/*const unsigned int anthesis = 2150; //anthesis/flowering time

const unsigned int harvest = 3100; //harvest time

//const double max_area_under_HAD = 1699.61;

const double max_area_under_HAD = 1700.75;//*/

//const unsigned int l1_ini = 1635;

//const unsigned int l1_sen = 2725;

//const double max_area_under_HAD = 2910.66;

/////////////////////////////////////////////////////////////////////////////////////////

// The below four constants are for how the program is run.

// AUTOMATE - if you want to loop the program within a bat script, this

// has to be set to true. Otherwise the program will

// display a list of parameter values and expect a prompt from

// you, preventing automation.

//

// If you set this to true and run it in Visual Studio (F5) it

// will crash. This is because it is expecting fungicide dose

// files in ARGV.

//

// END_FIRST_YEAR Will end the program after one year, before resistance builds

// up. Useful if you want to test the program.

//

// KEEP_RUNNING Ignores HAD calculation, and so the program will run infinitely.

//

// PARAMETER_SEARCH This also expects Gamma and Rho to be input via the command line

// used for cultivar resistance stuff

/////////////////////////////////////////////////////////////////////////////////////////

const bool keep_running = false; //flag to determine whether the program should ignore the reduction

//in HAD and never quit

const bool end_first_year = false; //Do we run for only one growing season?

bool in_control = true; //If keep_running is false, then the program will end when

//HAD < 0.95*max_area_under_HAD, and this variable is set to false

double HAD_area = 0.0; //stores the current HAD value

double sum_sensitive = 0.0; //sum of sensitive infectious area at the end of the growing season

double sum_resistant = 0.0; //sum of resistant infectious area at the end of the growing season

double AUDPC1 = 0.0; //Area under the disease progress curve for window 1

double AUDPC2 = 0.0; //Area under the disease progress curve for window 2

const double maxAUDPC1 = 4.24417; //Maximum AUDPC value (no fungicide) for period one (winter)

const double maxAUDPC2 = 125.37; //Maximum AUDPC for period two (spring)

///////////////////////////////////////////////////////////////////////////////////////////////

// Below variables are for AUDPC calculation

///////////////////////////////////////////////////////////////////////////////////////////////

const unsigned int AUDPC_1_start = 500;

const unsigned int AUDPC_1_end = 1200;

const unsigned int AUDPC_2_start = 1400;

const unsigned int AUDPC_2_end = 2100;

vector<double> audpc_vec_1; //Infectious area index points over time, between flowering and harvest

vector<double> audpc_vec_2; //Infectious area index points over time, between flowering and harvest

vector<double> x_audpc_1_vec;

vector<double> x_audpc_2_vec;

vector<double> x_times; //stores the time steps that occured between flowering and harvest

vector<double> had_values; //stores the sum of the healthy and latent area indexes at the

//points in x_times

//set up our bookkeeping variables for our latent compartments (refer to the header file for the values of these variables).

unsigned int parameters::L2i_s = 6;

unsigned int parameters::L2i_r = parameters::L2i_s + ( parameters::no_of_latent_compartments - 1 );

//and for our seed treatment fungicide bookkeeping variables

unsigned int parameters::Nseed_h = parameters::L2i_r + ( parameters::no_of_latent_compartments - 1 );

unsigned int parameters::N_layer_i_h = parameters::Nseed_h + 1;

unsigned int parameters::Nseed_l = parameters::N_layer_i_h + 1;

unsigned int parameters::N_layer_i_l = parameters::Nseed_l + 1;

double parameters::trans_asymp_val = 0;

double parameters::trans_intercept = 0;

//and the rounding multiplier

unsigned int parameters::h_MOD = 0;

const unsigned int last_leaf_for_HAD_calc = 3;

const unsigned int last_leaf_for_resist_calc = 5;

const unsigned int max_years = 11;

double calculateArea(const std::vector<double>& x, const std::vector<double>& y){

// Calculate the area under a curve, when x and y are specified in vectors

if (x.size() != y.size()){

std::cerr << "Cannot calculate the area; vectors are not the same size" << std::endl;

exit(1);

}

double area = 0.0;

for (std::size_t element = 1; element != x.size(); ++element){

area += 0.5 * (y[element] + y[element-1]) * (x[element]-x[element-1]);

}

return area;

}

/**

* checks with the HAD (the total healthy area and latent area for leaf layers 1-3

* is still within 5% of the total area (it will never exceed the area).

*

* @return - true/false for whether we are still within this measure of 'control'.

*/

bool still_in_control( list<leafLayer*> * layers ){

if( keep_running ){

return true;

}

n = x_times.size();

audpc1_n = x_audpc_1_vec.size();

audpc2_n = x_audpc_2_vec.size();//*/

double * HAD_arr = new double[x_times.size()];

double * x_times_arr = new double[x_times.size()];

double * audpc_arr_1 = new double[x_audpc_1_vec.size()];

double * x_times_audpc1 = new double[x_audpc_1_vec.size()];

double * audpc_arr_2 = new double[x_audpc_2_vec.size()];

double * x_times_audpc2 = new double[x_audpc_2_vec.size()];

HAD_area = 0.0;

AUDPC1 = 0.0;

AUDPC2 = 0.0;

//initialise these arrays

for( int i = 0; i < x_times.size(); i ++ ) {HAD_arr[i] = 0.0; x_times_arr[i] = 0.0;}

for( int i = 0; i < x_audpc_1_vec.size(); i ++ ){audpc_arr_1[i] = 0.0; x_times_audpc1[i] = 0.0;}

for( int i = 0; i < x_audpc_2_vec.size(); i ++ ){audpc_arr_2[i] = 0.0; x_times_audpc2[i] = 0.0;}

//from JK_utils

convert_vec_to_array( x_times_arr, x_times );

convert_vec_to_array( HAD_arr, had_values );

convert_vec_to_array( x_times_audpc1, x_audpc_1_vec );

convert_vec_to_array( audpc_arr_1, audpc_vec_1 );

convert_vec_to_array( x_times_audpc2, x_audpc_2_vec );

convert_vec_to_array( audpc_arr_2, audpc_vec_2 );

//first get our HAD area

// GET HAD ARES

HAD_area = calculateArea(x_times, had_values);

// GET AUDPC1

AUDPC1 = calculateArea(x_audpc_1_vec,audpc_vec_1);

// GET AUDPC2

AUDPC2 = calculateArea(x_audpc_2_vec, audpc_vec_2);

//delete our pointers to prevent leaks

HAD_arr = 0; delete [] HAD_arr;

x_times_arr = 0; delete [] x_times_arr;

audpc_arr_1 = 0; delete [] audpc_arr_1;

audpc_arr_2 = 0; delete [] audpc_arr_2;

x_times_audpc1 = 0; delete [] x_times_audpc1;

x_times_audpc2 = 0; delete [] x_times_audpc2;

//now check for control

if( HAD_area < parameters::HAD_threshold*max_area_under_HAD ){

return false;

}else{

return true;

}//*/

}//end method

double roundTS( double tsim ){

// Round tsim to nearest time step

double newtime = tsim * 1/parameters::h + 0.5;

newtime = floor( newtime );

newtime *= parameters::h;

return newtime;

}

int main( int argc, char * argv[] ){

unsigned int year = 0; //how many years have we got control?

//parameters::trans_asymp_val = 0.02;

parameters::trans_asymp_val = 0.027;

parameters::trans_intercept = ((double)20 / (double)254)*parameters::trans_asymp_val;

parameters::print_params_to_terminal();

parameters::write_params_to_file();

parameters::calc_h_MOD();

parameters::print_bookkeeping_variables();

//our high risk fungicide object, inheriting from the foliar and seed treatment classes

fungicide_st high_risk_fung( parameters::foliar_breakdown_h, parameters::st_breakdown_h, parameters::st_uptake_h, parameters::Nseed_h, parameters::N_layer_i_h );

//our low risk fungicide object, inheriting from the foliar and seed treatment classes

fungicide_st low_risk_fung ( parameters::foliar_breakdown_l, parameters::st_breakdown_l, parameters::st_uptake_l, parameters::Nseed_l, parameters::N_layer_i_l );

//lets read in our doses from file

string high_foliar_file = "high_risk_foliar_times.txt";

string high_st_file = "high_ST_input_dose.txt";

string low_foliar_file = "low_risk_foliar_times.txt";

string low_st_file = "low_ST_input_dose.txt";

high_risk_fung.read_fungicide_dose_data( high_foliar_file );

high_risk_fung.read_ST_dose_data( high_st_file );

low_risk_fung.read_fungicide_dose_data( low_foliar_file );

low_risk_fung.read_ST_dose_data( low_st_file );

//make our list of our leaf layer objects

list<leafLayer*> * layers = leafLayer::makeLeafLayers();

epidemic_st epidem( layers, high_risk_fung, low_risk_fung ); //our epidemic object initialised with the number of latent compartments

//and the layers vector

fstream densityFileCols; //file stream for writing density output to (columns).

fstream ST_bar_conc_File; //file stream for printint seed treatment concentration as a bar plot per layer

fstream res_prop; //prints out the proportion of the resistant strain in the population

fstream effective_life_rcrd; //writes out step sizes used, which may become altered due to boundaries

fstream HADperyr; //prints out our y points for leaf 1-3 healthy area from flowering till harvest

map<double, double> high_risk_spray_times = high_risk_fung.get_spray_times_hash();

map<double, double> low_risk_spray_times = low_risk_fung.get_spray_times_hash();

double high_risk_init_N_seed = high_risk_fung.get_Nseed();

double low_risk_init_N_seed = low_risk_fung.get_Nseed();

densityFileCols.open( "density_out_cols.txt", ios::out | ios::trunc );

res_prop.open( "res_prop.txt", ios::out | ios::trunc );

effective_life_rcrd.open( "effective_life_rcrd.txt", ios::out | ios::trunc );

//Set up the columns in our severity files and effective life file

double high_risk_ST_prop = 0.0;

double low_risk_ST_prop = 0.0;

if( high_risk_init_N_seed != 0.0 ){high_risk_ST_prop = high_risk_init_N_seed / parameters::maxSTDose;}

if( low_risk_init_N_seed != 0.0 ){ low_risk_ST_prop = low_risk_init_N_seed / parameters::maxSTDose; }

if( high_risk_ST_prop > 1.0 ){

cout<<"Error with initial dose "<<high_risk_init_N_seed<<" "<<high_risk_ST_prop<<"\n"; exit( 0 );

}

if( low_risk_ST_prop > 1.0 ){

cout<<"Error with initial dose "<<low_risk_init_N_seed<<" "<<low_risk_ST_prop<<"\n"; exit( 0 );

}

effective_life_rcrd<<"Effective life (years)\tHAD\tmax HAD\tpercentage of max\n";

res_prop<<"1=2\n";

//Lets write our doses out to our dose files so we know what we've applied

map<double, double>::iterator f_spray_it;

for( f_spray_it = high_risk_spray_times.begin(); f_spray_it != high_risk_spray_times.end(); f_spray_it ++ ){

double t = (*f_spray_it).first;

double h_dose = (*f_spray_it).second;

double h_prop = 0.0;

if( h_dose != 0.0 ){ h_prop = h_dose / parameters::maxFoliarDose; }

}//end for

for( f_spray_it = low_risk_spray_times.begin(); f_spray_it != low_risk_spray_times.end(); f_spray_it ++ ){

double t = (*f_spray_it).first;

double l_dose = (*f_spray_it).second;

double l_prop = 0.0;

if( l_dose != 0.0 ){ l_prop = l_dose / parameters::maxFoliarDose; }

}//end for

leafLayer::write_densities_column_names( densityFileCols );

while( in_control ){

year ++;

cout<<"Year "<<year<<"\n";

//every year after the first we recalculate the proportion of resistant fungi

if( year > 1 ){

epidem.calc_prop_of_resistant( sum_sensitive, sum_resistant, res_prop );

//below values should now be zero

}

/*

* loop through all time steps according to time step h. We will take a new step size if

* there is a boundary, which is the variable we initialise directly below

*/

double step_size = 0.0;

bool use_remainder_of_stepsize = false;

double high_spray_dose_sum = 0;

double low_spray_dose_sum = 0;

bool has_sprayed = false;

for( double t = 0.0; t < harvest; ){

if( !use_remainder_of_stepsize ){t = roundTS( t );}

list<leafLayer*>::iterator it; //iterator object for all leaf layers

for( it = layers->begin(); it != layers->end(); it ++ ){

leafLayer * currentLayer = (*it);

//update leaf layer life cycle

currentLayer->check_life_cycle_stage( t, sum_sensitive, sum_resistant, last_leaf_for_resist_calc );

//spray leaf if at correct time

if( map_contains_with_modifier( high_risk_spray_times, t, parameters::h_MOD ) ){

double high_risk_init_dose = high_risk_fung.calculate_dose_per_leaf_on_spraying( currentLayer, layers, t );

high_spray_dose_sum += high_risk_init_dose;

has_sprayed = true;

cout<<"leaf "<<currentLayer->getLayerNo()<<" Spraying at time "<<t<<" high_risk_init_dose "<<high_risk_init_dose<<" max dose "<<high_risk_spray_times.at( t )<<endl;

currentLayer->add_high_risk_foliar_dose( high_risk_init_dose, t );

}//*/

//spray leaf if at correct time

if( map_contains_with_modifier( low_risk_spray_times, t, parameters::h_MOD ) ){

double low_risk_init_dose = low_risk_fung.calculate_dose_per_leaf_on_spraying( currentLayer, layers, t );

low_spray_dose_sum += low_risk_init_dose;

has_sprayed = true;

//cout<<"Spraying at time "<<t<<" low_risk_init_dose "<<low_risk_init_dose<<endl;

currentLayer->add_low_risk_foliar_dose( low_risk_init_dose, t );

}//*/

}//end for

if( has_sprayed ){

has_sprayed = false;

high_spray_dose_sum = 0;

low_spray_dose_sum = 0;

}

/**

* Here we look at whether we have come to any boundaries, which will alter the step size.

* the new step size is returned by epidem.calculate_disease_ODEs(), which if there has

* been a boundary will give t_boundary - t as the new step size. The simulation is then

* iterated according to this new step size.

*

* However, we still, in the next iteration, will need to iterate in time by the remainder

* of the previous step size. For example, if we have a default step size of 0.3, and there

* is a boundary 0.1 time units away, epidem.calculate_disease_ODEs() will return a step size of

* 0.1. In the next iteration, however, we will need to iterate by 0.3 - 0.1 = 0.2 time steps.

* if there are no further boundaries, the step size will return to 0.3.

*

* the boolean use_remainder_of_stepsize tells us if we are on the second iteration that must

* use this remainder. If this is TRUE, and there are no further boundaries: step_size = h - the

* previous value of step_size. If there is a further boundary, we will know because the value

* returned by epidem.calculate_disease_ODEs() != h, and so we keep use_remainder_of_stepsize as

* TRUE and go through again.

*/

if( use_remainder_of_stepsize ){

step_size = parameters::h - step_size;

step_size = epidem.calculate_disease_ODEs( step_size, layers, t );

use_remainder_of_stepsize = false;

}else{

step_size = epidem.calculate_disease_ODEs( parameters::h, layers, t );

if( step_size != parameters::h ){

use_remainder_of_stepsize = true;

}

cout<<"";

}

//Loop through all leaf layers and print out severity and density info

for( it = layers->begin(); it != layers->end(); it ++ ){

leafLayer * currentLayer = (*it);

if( currentLayer->isActive() && fmod( t, 1.0 ) == 0 ){

currentLayer->write_densities_column( t, densityFileCols, year );

}//end if

}//end nested for

//now retrieve HAD (healthy plus latent area) for leaf layers (layers 1 - 3 only)

if( t >= anthesis ){

double H_i_sum = 0.0;

double I_i_sum = 0.0;

for( it = layers->begin(); it != layers->end(); it ++ ){

leafLayer * currentLayer = (*it);

if( currentLayer->getLayerNo() <= last_leaf_for_HAD_calc && currentLayer->isActive() ){

H_i_sum += currentLayer->get_healthy_area() + currentLayer->getTotalLatentArea();

}//end if

}//end for

had_values.push_back( H_i_sum );

x_times.push_back( t );

}//end if

if( t >= AUDPC_1_start && t < AUDPC_1_end ){

double sum_infectious = 0;

for( it = layers->begin(); it != layers->end(); it ++ ){

leafLayer * currentLayer = (*it);

sum_infectious += currentLayer->getSensitiveInfectiousArea() + currentLayer->getResistantInfectiousArea();

}//end for

audpc_vec_1.push_back( sum_infectious );

x_audpc_1_vec.push_back( t );

}//end if

if( t >= AUDPC_2_start && t < AUDPC_2_end ){

double sum_infectious = 0;

for( it = layers->begin(); it != layers->end(); it ++ ){

leafLayer * currentLayer = (*it);

sum_infectious += currentLayer->getSensitiveInfectiousArea() + currentLayer->getResistantInfectiousArea();

}//end for

audpc_vec_2.push_back( sum_infectious );

x_audpc_2_vec.push_back( t );

}//end if

if( t > 2094 && t < 2095 ){

cout<<"t: "<<t<<" leaf 5 death - high ST amount = "<<high_risk_fung.get_ST_percent_of_initial()<<"\n";

cout<<"t: "<<t<<" leaf 5 death - low ST amount = "<<low_risk_fung.get_ST_percent_of_initial()<<"\n";

cout<<"";

}

if( t == 500 || t == 750 || t == 1000 || t == 1250 || t == 1500 || t == 1725 || t == 2000 || t == 2225 || t == 2500 || t == 2725 || t == 3000 ){

cout<<"t = "<<t<<"\n";

}//end if

t += step_size;

//cout<<"t = "<<t<<" step_size = "<<step_size<<"\n";

//cout<<"";

}//end for time t

//replenish seed treatment fungicide

high_risk_fung.replenish_ST_fungicide();

low_risk_fung.replenish_ST_fungicide();

//check if we are still in control

cout<<"Year "<<year<<" calculating HAD\n";

in_control = still_in_control( layers );

effective_life_rcrd<<year<<"\t"<<HAD_area<<"\t"<<max_area_under_HAD

<<"\t"<<((HAD_area/max_area_under_HAD)*100)<<"\n";

//clear our x and y value arrays for HAD, for the next growing season

x_times.clear();

had_values.clear();

x_audpc_1_vec.clear();

audpc_vec_1.clear();

audpc_vec_2.clear();

x_audpc_2_vec.clear();

if( end_first_year ){

break;

}//*/

if( year == max_years ){

break;

}//*/

}//end while

double HAD_sum = leafLayer::totalHealthyArea + leafLayer::totalLatentArea;

double area = leafLayer::totalArea;

cout<<"\n Year "<<year<<" control has been lost, HAD = "<<HAD_area<<" percent of max "<<((HAD_area/max_area_under_HAD)*100)<<". Exiting\n";

cout<<"AUDPC 1: "<<AUDPC1<<" AUDPC 2 "<<AUDPC2<<"\n";

cout<<"percent_1 "<<(AUDPC1/maxAUDPC1)*100<<"\n";

cout<<"percent_2 "<<(AUDPC2/maxAUDPC2)*100<<"\n";

epidem.calc_prop_of_resistant( sum_sensitive, sum_resistant, res_prop );

return 0;

}

/**

* Prints out our parameters to the file "param_values.txt"

*/

void parameters::write_params_to_file(){

fstream param;

param.open( "param_values.txt", ios::out | ios::trunc );

if( !param.good() ){

cout<<"Error with param output file! Quitting\n";exit( 1 );

}

param<<"1 2";

param<<"\ngrowth_rate "<<parameters::growthRate;

param<<"\nsenescence_rate "<<parameters::s_rate;

param<<"\na "<<parameters::a;

param<<"\ntau "<<parameters::tau;

param<<"\nleaf_thickness "<<parameters::leaf_thickness;

param<<"\nomega "<<parameters::omega;

param<<"\neta "<<parameters::eta;

param<<"\nlambda "<<parameters::lambda;

param<<"\ntheta_zero "<<parameters::theta_zero;

param<<"\nsigma_up "<<parameters::sigma_up;

param<<"\nsigma_down "<<parameters::sigma_down;

param<<"\nmu "<<parameters::mu;

param<<"\nfoliar_breakdown_h "<<parameters::foliar_breakdown_h;

param<<"\nst_breakdown_h "<<parameters::st_breakdown_h;

param<<"\nst_uptake_h "<<parameters::st_uptake_h;

param<<"\nfoliar_breakdown_l "<<parameters::foliar_breakdown_l;

param<<"\nst_breakdown_l "<<parameters::st_breakdown_l;

param<<"\nst_uptake_l "<<parameters::st_uptake_l;

param<<"\nalpha_delta_h "<<parameters::alpha_delta_h;

param<<"\nalpha_epsilon_h "<<parameters::alpha_epsilon_h;

param<<"\nalpha_rho_h "<<parameters::alpha_rho_h;

param<<"\nalpha_delta_l "<<parameters::alpha_delta_l;

param<<"\nalpha_epsilon_l "<<parameters::alpha_epsilon_l;

param<<"\nalpha_rho_l "<<parameters::alpha_rho_l;

param<<"\nk_delta_s_h "<<parameters::k_delta_s_h;

param<<"\nk_epsilon_s_h "<<parameters::k_epsilon_s_h;

param<<"\nk_rho_s_h "<<parameters::k_rho_s_h;

param<<"\nk_delta_s_l "<<parameters::k_delta_s_l;

param<<"\nk_epsilon_s_l "<<parameters::k_epsilon_s_l;

param<<"\nk_rho_s_l "<<parameters::k_rho_s_l;

param<<"\ngamma_default "<<parameters::gamma_default;

param<<"\nrho_default "<<parameters::rho_default;

param<<"\ndelta_default "<<parameters::delta_default;

param<<"\nmaximum_extension "<<parameters::maximum_extension;

param<<"\nNo_latent_compts "<<parameters::no_of_latent_compartments;

param<<"\nstep_size "<<parameters::h;

param<<"\nanthesis "<<anthesis;

param<<"\nharvest "<<harvest;

param<<"\nmax_HAD "<<max_area_under_HAD;

param<<"\nHAD_threshold "<<parameters::HAD_threshold;

param<<"\ntrans_intercept "<<parameters::trans_intercept;

param<<"\ntrans_coefficient "<<parameters::trans_coefficient;

param<<"\ntrans_asymp_val "<<parameters::trans_asymp_val;

param<<"\ntrans_growth_rate "<<parameters::trans_growth_rate;

param <<"\nUsing_transpiration_pull " << parameters::using_transpiration;

param<<"\nlast_leaf_for_HAD_calc "<<last_leaf_for_HAD_calc;

param<<"\nlast_leaf_for_resist_calc "<<last_leaf_for_resist_calc;

param<<"\nmaxSTDose "<<maxSTDose;

param.close();

}//end method

/**

* Prints out our parameters to the file "param_values.txt"

* @param param - file to write the parameters to

*/

void parameters::write_params_to_file( fstream & param ){

param<<"1 2\n";

param<<"\n\n\n************************\nSimulation parameters\n";

param<<"\ngrowth_rate "<<parameters::growthRate;

param<<"\nsenescence_rate "<<parameters::s_rate;

param<<"\na "<<parameters::a;

param<<"\ntau "<<parameters::tau;

param<<"\nleaf_thickness "<<parameters::leaf_thickness;

param<<"\nomega "<<parameters::omega;

param<<"\neta "<<parameters::eta;

param<<"\nlambda "<<parameters::lambda;

param<<"\ntheta_zero "<<parameters::theta_zero;

param<<"\nsigma_up "<<parameters::sigma_up;

param<<"\nsigma_down "<<parameters::sigma_down;

param<<"\nmu "<<parameters::mu;

param<<"\nfoliar_breakdown_h "<<parameters::foliar_breakdown_h;

param<<"\nst_breakdown_h "<<parameters::st_breakdown_h;

param<<"\nst_uptake_h "<<parameters::st_uptake_h;

param<<"\nfoliar_breakdown_l "<<parameters::foliar_breakdown_l;

param<<"\nst_breakdown_l "<<parameters::st_breakdown_l;

param<<"\nst_uptake_l "<<parameters::st_uptake_l;

param<<"\nalpha_delta_h "<<parameters::alpha_delta_h;

param<<"\nalpha_epsilon_h "<<parameters::alpha_epsilon_h;

param<<"\nalpha_rho_h "<<parameters::alpha_rho_h;

param<<"\nalpha_delta_l "<<parameters::alpha_delta_l;

param<<"\nalpha_epsilon_l "<<parameters::alpha_epsilon_l;

param<<"\nalpha_rho_l "<<parameters::alpha_rho_l;

param<<"\nk_delta_s_h "<<parameters::k_delta_s_h;

param<<"\nk_epsilon_s_h "<<parameters::k_epsilon_s_h;

param<<"\nk_rho_s_h "<<parameters::k_rho_s_h;

param<<"\nk_delta_s_l "<<parameters::k_delta_s_l;

param<<"\nk_epsilon_s_l "<<parameters::k_epsilon_s_l;

param<<"\nk_rho_s_l "<<parameters::k_rho_s_l;

param<<"\ngamma_default "<<parameters::gamma_default;

param<<"\nrho_default "<<parameters::rho_default;

param<<"\ndelta_default "<<parameters::delta_default;

param<<"\nmaximum_extension "<<parameters::maximum_extension;

param<<"\nNo_latent_compts "<<parameters::no_of_latent_compartments;

param<<"\nstep_size "<<parameters::h;

param<<"\nanthesis "<<anthesis;

param<<"\nharvest "<<harvest;

param<<"\nmax_HAD "<<max_area_under_HAD;

// param<<"\ny "<<parameters::y;

param<<"\nHAD_threshold "<<parameters::HAD_threshold;

param<<"\n\n************************\n";

}//end method

/**

* Prints out the parameters to the terminal and asks user if they are

* happy with the chosen values

*/

void parameters::print_params_to_terminal(){

cout<<"\n\n\n************************\nSimulation parameters\n";

cout<<"\ngrowth_rate "<<parameters::growthRate;

cout<<"\nsenescence_rate "<<parameters::s_rate;

cout<<"\na "<<parameters::a;

cout<<"\ntau "<<parameters::tau;

cout<<"\nleaf_thickness "<<parameters::leaf_thickness;

cout<<"\nomega "<<parameters::omega;

cout<<"\neta "<<parameters::eta;

cout<<"\nlambda "<<parameters::lambda;

cout<<"\ntheta_zero "<<parameters::theta_zero;

cout<<"\nsigma_up "<<parameters::sigma_up;

cout<<"\nsigma_down "<<parameters::sigma_down;

cout<<"\nmu "<<parameters::mu;

cout<<"\nfoliar_breakdown_h "<<parameters::foliar_breakdown_h;

cout<<"\nst_breakdown_h "<<parameters::st_breakdown_h;

cout<<"\nst_uptake_h "<<parameters::st_uptake_h;

cout<<"\nfoliar_breakdown_l "<<parameters::foliar_breakdown_l;

cout<<"\nst_breakdown_l "<<parameters::st_breakdown_l;

cout<<"\nst_uptake_l "<<parameters::st_uptake_l;

cout<<"\nalpha_delta_h "<<parameters::alpha_delta_h;

cout<<"\nalpha_epsilon_h "<<parameters::alpha_epsilon_h;

cout<<"\nalpha_rho_h "<<parameters::alpha_rho_h;

cout<<"\nalpha_delta_l "<<parameters::alpha_delta_l;

cout<<"\nalpha_epsilon_l "<<parameters::alpha_epsilon_l;

cout<<"\nalpha_rho_l "<<parameters::alpha_rho_l;

cout<<"\nk_delta_s_h "<<parameters::k_delta_s_h;

cout<<"\nk_epsilon_s_h "<<parameters::k_epsilon_s_h;

cout<<"\nk_rho_s_h "<<parameters::k_rho_s_h;

cout<<"\nk_delta_s_l "<<parameters::k_delta_s_l;

cout<<"\nk_epsilon_s_l "<<parameters::k_epsilon_s_l;

cout<<"\nk_rho_s_l "<<parameters::k_rho_s_l;

cout<<"\ngamma_default "<<parameters::gamma_default;

cout<<"\nrho_default "<<parameters::rho_default;

cout<<"\ndelta_default "<<parameters::delta_default;

cout<<"\nmaximum_extension "<<parameters::maximum_extension;

cout<<"\nNo_latent_compts "<<parameters::no_of_latent_compartments;

cout<<"\nstep_size "<<parameters::h;

cout<<"\nanthesis "<<anthesis;

cout<<"\nharvest "<<harvest;

cout<<"\nmax_HAD "<<max_area_under_HAD;

cout<<"\nHAD_threshold "<<parameters::HAD_threshold;

cout<<"\ntrans_intercept "<<parameters::trans_intercept;

cout<<"\ntrans_coefficient "<<parameters::trans_coefficient;

cout<<"\ntrans_asymp_val "<<parameters::trans_asymp_val;

cout<<"\ntrans_growth_rate "<<parameters::trans_growth_rate;

cout<<"\nUsing_transpiration_pull "<<parameters::using_transpiration;

cout<<"\nlast_leaf_for_HAD_calc "<<last_leaf_for_HAD_calc;

cout<<"\nlast_leaf_for_resist_calc "<<last_leaf_for_resist_calc;

cout<<"\nmaxSTDose "<<maxSTDose;

cout<<"\n\n************************\n";

if( growthRate != 0.034 ){

string ans;

cout<<"growthRate = "<<growthRate<<" - proceed?\n";

cin>>ans;

if( ans == "n" ){exit( 0 );}

}

}//end method

/**

* Simply prints out the values of these bookkeeping

* variables for debugging purposes

*/

void parameters::print_bookkeeping_variables(){

cout<<"Hi = "<<Hi<<"\n";

cout<<"Ai = "<<Ai<<"\n";

cout<<"L1i_s = "<<L1i_s<<"\n";

cout<<"L1i_r = "<<L1i_r<<"\n";

cout<<"Ii_s = "<<Ii_s<<"\n";

cout<<"Ii_r = "<<Ii_r<<"\n";

cout<<"L2i_s = "<<L2i_s<<"\n";

cout<<"L2i_r = "<<L2i_r<<"\n";

cout<<"Nseed_h = "<<Nseed_h<<"\n";

cout<<"N_layer_i_h = "<<N_layer_i_h<<"\n";

cout<<"Nseed_l = "<<Nseed_l<<"\n";

cout<<"N_layer_i_l = "<<N_layer_i_l<<"\n";

cout<<"";

}//end method

/**

* Calculates h_MOD, the value to multiply time steps by, according

* to the input step size. If time step is >= 1.0 and < 0.1, the mod

* will be 10, if >= 0.1 and < 0.01, 100 and 1 otherwise

*/

void parameters::calc_h_MOD(){

if( parameters::h >= 0.1 && parameters::h < 1.0 ){

parameters::h_MOD = 10;

}else if( parameters::h > 0.01 && parameters::h <= 0.1 ){

parameters::h_MOD = 100;

}else if( parameters::h > 0.001 && parameters::h <= 0.01 ){

parameters::h_MOD = 1000;

}else{

cout<<"Warning - your time step of "<<parameters::h<<" will lead to a "<<

"h_MOD value of 1 - are you happy with this? (y/n)";

string ans = "";

cin>>ans;

if( ans != "y" ){exit(0);}

cout<<"\n";

}

cout<<"h_MOD = "<<h_MOD<<"\n";

cout<<"";

}

**Parameters.h**

#include <fstream>

#include <iostream>

namespace parameters{

const double h = 0.1; //step size

/**

* Plant growth parameters

*/

const double growthRate = 0.034;

const double h_death = 0.01;

const double phyllochron_length = 122.0;

const double s_rate = 0.05;

const double a = 1.0;

const double tau = 0.77;

const double leaf_thickness = 0.001;

const double omega = 0.001;

const double HAD_threshold = 0.95;

const unsigned short int maximum_extension = 10;

const bool using_transpiration = true;

const double trans_coefficient = 0.0015;

const double trans_growth_rate = 0.003;

extern double trans_asymp_val;

extern double trans_intercept;

/**

* Disease parameters

*/

const double eta = 1.0;

const double lambda = 0.0035;

const double theta_zero = 0.00001;

//const double theta_zero = 0.0;

const double sigma_up = 0.1;

const double sigma_down = 0.01;

const double mu = 0.002;

const double gamma_default = 4e-10;

const double rho_default = 0.007;

const double delta_default = 0.004;

const double ex_rate = 0.1;

//const double y = 0;

const unsigned short int no_of_latent_compartments = 10;

/**

* Fungicide parameters

*/

const double maxFoliarDose = 20.0; //maximum foliar dose, for expressing the input dose as a proportion

// Changed this max from 2.15

const double maxSTDose = 5.0; //maximum seed treatment dose, for expressing the input dose as a proportion

/*

* High risk

*/

const double st_uptake_h = 0.0055;

//params for low breakdown

const double st_breakdown_h = 0.0046;

const double foliar_breakdown_h = 0.0046;

const double alpha_delta_h = 0.45;

const double alpha_epsilon_h = 0.45;

const double alpha_rho_h = 0.45;

const double k_delta_s_h = 0.0025;

const double k_epsilon_s_h = 0.0025;

const double k_rho_s_h = 0.0025;//*/

//params for high breakdown

/*const double st_breakdown_h = 0.009;

const double foliar_breakdown_h = 0.009;

const double alpha_delta_h = 0.55;

const double alpha_epsilon_h = 0.55;

const double alpha_rho_h = 0.55;

const double k_delta_s_h = 0.003;

const double k_epsilon_s_h = 0.003;

const double k_rho_s_h = 0.003;//*/

/**

* Fungicide parameters

*

* Low risk

*/

const double st_uptake_l = 0.0055;

const double st_breakdown_l = 0.0028;

const double foliar_breakdown_l = 0.0028;

/*const double alpha_delta_l = 0.45;

const double alpha_epsilon_l = 0.45;

const double alpha_rho_l = 0.45; */

const double alpha_delta_l = 0.225;

const double alpha_epsilon_l = 0.225;

const double alpha_rho_l = 0.225;

const double k_delta_s_l = 0.0008;

const double k_epsilon_s_l = 0.0008;

const double k_rho_s_l = 0.0008;

/**

* Bookkeeping variables/constants

*/

enum variables{ //enum for the variables used in our variable_array.

//To help with bookkeeping

Ai, //0

Hi, //1

L1i_s, //2

L1i_r, //3

Ii_s, //4

Ii_r //5

};//end enum

/**

* We declare the following four bookeeping variables as extern as we want the definitions to be seen across all

* different compilation units (.cpp files).

*/

extern unsigned int L2i_s; //bookkeeping value for where the second sensitive latent compartment is stored

extern unsigned int L2i_r; //bookkeeping value for where the second sensitive latent compartment is stored

extern unsigned int Nseed_h; //bookkeeping value for where the derivative/value of N_seed, the amount of seed

//high risk fungicide is left in the seed.

extern unsigned int N_layer_i_h; //bookkeeping value for where the derivative/value for the amount of

//high risk systemic fungicide in the leaf layer is kept

extern unsigned int Nseed_l; //bookkeeping value for where the derivative/value of N_seed, the amount of seed

//low risk fungicide is left in the seed.

extern unsigned int N_layer_i_l; //bookkeeping value for where the derivative/value for the amount of

//low risk systemic fungicide in the leaf layer is kept

extern unsigned int h_MOD; //modifier to mulitply time steps by to avoid floating point errors

void write_params_to_file();

void print_params_to_terminal();

void print_bookkeeping_variables();

void write_params_to_file( std::fstream & param );

void calc_h_MOD();

}//end namespace

**seed_treatment_fungicide.cpp**

#include "seed_treatment_fungicide.h"

#include "parameters.h"

using namespace std;

/**

* Constructor.

*/

seed_treatment_fungicide::seed_treatment_fungicide( const double & ST_breakdownIn, const double & ST_uptakeIn,

unsigned int & N_seed_element_noIn, unsigned int N_layer_element_noIn ){

ST_breakdown = ST_breakdownIn;

ST_uptake = ST_uptakeIn;

N_seed_element_no = N_seed_element_noIn;

N_layer_element_no = N_layer_element_noIn;

}

/**

* returns the transpiration rate as a logistic function,

* with parameters estimated using data from Ober et al

* multiplied by a certain maximum (y)

*

* @param t = time point

* @return - current transpiration rate

*/

double seed_treatment_fungicide::get_trans_rate( double t ){

double u = parameters::trans_intercept;

double c = parameters::trans_coefficient;

double y = parameters::trans_asymp_val;

double a = parameters::trans_growth_rate;

return u + ( y*c*exp( a*t ) ) / ( c*exp( a*t ) + ( 1 - c ) );

}

/**

* Contains the ODE for the rate of change of the seed treatment reservoire

* to be solved by RK4

*

* As all other variables are per leaf layer, even though this is not, the

* same value has to be recorded per leaf layer, unfortunately.

*

* @param layer_i - leaf layer object (all leaf layers

* will keep the seed amount value

* @param layer_temp_variable_map - map of layers and their initial conditions

* @param layer_derivative_map - map of layers and their derivatives

*/

void seed_treatment_fungicide::ST_seed_deriv( leafLayer * layer_i,

map<leafLayer*, vector<long double>*> & layer_temp_variable_map,

map<leafLayer*, vector<long double>*> & layer_derivative_map, double t ){

//get our arrays from our map

vector<long double> * variable_array = layer_temp_variable_map.at( layer_i );

vector<long double> * derivative_array = layer_derivative_map.at( layer_i );

double dNseed_dt = 0.0;

double N_seed_value = variable_array->at( N_seed_element_no );

if( parameters::using_transpiration ){

dNseed_dt = -get_trans_rate( t ) * N_seed_value;

}

else{

dNseed_dt = -this->ST_uptake * N_seed_value;

}//end else

derivative_array->at( N_seed_element_no ) = dNseed_dt;

}

/**

* converts the amount of fungicide (in mg/m cubed) into concentration

*

* @param layer_i - leaf layer as to what ODE's we are currently solving

* @param layers - list of leaf layer objects

* @param t - current time

* @param layer_temp_variable_map - hash table containing initial conditions per leaf layer of our ODE's.

*

* @return - concentration of seed treatment fungicide in layer

*/

double seed_treatment_fungicide::calc_seed_treat_concentration( leafLayer * layer_i, list<leafLayer*>* layers,

map<leafLayer*, vector<long double>*> & layer_temp_variable_map,

double t ){

double conc = 0.0;

//get the array from the map

vector<long double> * variable_array = layer_temp_variable_map.at( layer_i );

double healthy_area_i = variable_array->at( parameters::Hi );

double N_layer_amount = variable_array->at( N_layer_element_no );

//check we don't divide by zero.

if( parameters::omega * healthy_area_i != 0.0 ){

conc = ( N_layer_amount / ( parameters::omega * healthy_area_i ) );

}

return conc;

}//end method

/**

* Calculates the amount of seed treatment fungicide molecules entering the leaf layer, according to

* the proportion of its area relative to the total area of the whole plant.

*

* @param layer_i - current leaf layer of interest

* @param layers - list of leaf layer objects

* @param t - current time

* @param layer_temp_variable_map - map of layers and their initial conditions

*

* @return - the current influx of seed treatment fungicide into layer i

*/

double seed_treatment_fungicide::calc_Zi( leafLayer * layer_i, list<leafLayer*>* layers, double t,

map<leafLayer*, vector<long double>*> & layer_temp_variable_map ){

double area_ratio = 0.0; //ratio of layer i's area to all other layers

double Z_i = 0.0;

vector<long double> * variable_array = layer_temp_variable_map.at( layer_i );

double fung_in_seed = variable_array->at( N_seed_element_no );

double healthy_area_layer_i = variable_array->at( parameters::Hi );

double flow = 0.0;

if( parameters::using_transpiration ){

flow = get_trans_rate(t) * fung_in_seed;

}

else{

flow = this->ST_uptake * fung_in_seed;

}//end else

double sum_of_all_layer_healthy_areas = 0.0;

//sum healthy area belonging to all active leaf layers

list<leafLayer*>::iterator it;

for( it = layers->begin(); it != layers->end(); it ++ ){

leafLayer * layer_j = (*it);

if( !layer_j->isActive() ){

continue;

}

vector<long double> * variable_array_layer_j = layer_temp_variable_map.at( layer_j );

sum_of_all_layer_healthy_areas += variable_array_layer_j->at( parameters::Hi );

}

//make sure we don't divide by zero.

if( healthy_area_layer_i == 0.0 || sum_of_all_layer_healthy_areas == 0.0 ){

return 0.0;

}else{

area_ratio = healthy_area_layer_i / sum_of_all_layer_healthy_areas;

Z_i = flow * area_ratio;

return Z_i;

}

}//end method

/**

* Contains the ODE for the rate of change of the seed treatment dose

* stored in the leaf layer, to be solved by RK4

*

* As all other variables are per leaf layer, even though this is not, the

* same value has to be recorded per leaf layer, unfortunately.

*

* @param layer_i - leaf layer object (all leaf layers

* will keep the seed amount value

* @param layer_temp_variable_map - map of layers and their solved variables

* @param layer_derivative_map - map of layers and their derivatives

*/

void seed_treatment_fungicide::ST_layer_deriv( leafLayer * layer_i, std::list<leafLayer*>* layers, double t,

map<leafLayer*, vector<long double>*> & layer_temp_variable_map,

map<leafLayer*, vector<long double>*> & layer_derivative_map ){

//get our arrays from our map

vector<long double> * variable_array = layer_temp_variable_map.at( layer_i );

vector<long double> * derivative_array = layer_derivative_map.at( layer_i );

double fungicide_in_layer = variable_array->at( N_layer_element_no );

//now calc our values

double Z_i = this->calc_Zi( layer_i, layers, t, layer_temp_variable_map );

//calc our derivative

double senes = layer_i->senes_eq( t );

double dNi_dt = Z_i - this->ST_breakdown*fungicide_in_layer - senes*fungicide_in_layer;

derivative_array->at( N_layer_element_no ) = dNi_dt;

}

/**

* Reads in the intial dose of fungicide coating the seed at sowing.

* Quits if an empty file has been specified

*/

void seed_treatment_fungicide::read_ST_dose_data(){

cout<<"Reading in seed treatment input dose\n";

string line;

ifstream myFile( "ST_input_dose.txt" );

int no_lines = 0;

if( myFile.is_open() ){

while( myFile.good() ){

no_lines ++;

getline( myFile, line );

string dose;

vector<string> out = split( line, ' ' );

try{

dose = out.at( 1 );

}catch( out_of_range &e ){

continue;

}

initial_dose = atof( dose.c_str() );

N_seed = initial_dose;

}//end while

}//end if

if( no_lines == 0 ){

cout<<"Error, your seed treatment dose file is empty!\n";

exit( 1 );

}else if( no_lines > 1 ){

cout<<"Error, your seed treatment dose file contains more than one line - you will be overwriting previous doses.\n";

exit( 1 );

}

string ans;

cout<<"\n\n\n************************\nSeed treatment dose = "<<N_seed<<"\nAre you happy? y\\n\n";

cin>>ans;

if( ans == "n" ){exit( 0 );}

myFile.close();

}//end function

/**

* Reads in the intial dose of fungicide coating the seed at sowing.

* Quits if an empty file has been specified

*/

void seed_treatment_fungicide::read_ST_dose_data( string & fileName ){

cout<<"Reading in seed treatment input dose - file "<<fileName<<"\n";

string line;

ifstream myFile( fileName );

int no_lines = 0;

if( myFile.is_open() ){

while( myFile.good() ){

no_lines ++;

getline( myFile, line );

string dose;

vector<string> out = split( line, ' ' );

try{

dose = out.at( 1 );

}catch( out_of_range &e ){

continue;

}

initial_dose = atof( dose.c_str() );

N_seed = initial_dose;

cout<<"N_seed = "<<N_seed<<"\n";

}//end while

}else{

cout<<"Error in reading seed treatment file\n";

exit;

}//end else

if( no_lines == 0 ){

cout<<"Error, your seed treatment dose file is empty!\n";

exit( 1 );

}

cout<<"Seed treatment file read\n";

myFile.close();

}//end function

**seed_treatment_fungicide.h**

#ifndef SEED_TREATMENT_FUNGICIDE_H

#define SEED_TREATMENT_FUNGICIDE_H

#include <map>

#include <list>

#include <math.h>

#include <istream>

#include <fstream>

#include <string>

#include "leafLayer.h"

#include "JKUtils.h"

class seed_treatment_fungicide{

private:

double ST_breakdown;

double ST_uptake;

unsigned int N_seed_element_no;

unsigned int N_layer_element_no;

double get_trans_rate( double t );

public:

seed_treatment_fungicide(){}

seed_treatment_fungicide( const double & ST_breakdownIn, const double & ST_uptakeIn, unsigned int & N_seed_element_noIn, unsigned int N_layer_element_noIn );

void read_ST_dose_data(); //reads in the initial dose coating the seed, in mg m squared

void read_ST_dose_data( std::string & fileName ); //reads in the initial dose coating the seed, in mg m squared

void ST_seed_deriv( leafLayer * layer_i,

std::map<leafLayer*, std::vector<long double>*> & layer_temp_variable_map,

std::map<leafLayer*, std::vector<long double>*> & layer_derivative_map, double t );

double calc_Zi( leafLayer * layer_i, std::list<leafLayer*>* layers, double t,

std::map<leafLayer*, std::vector<long double>*> & layer_temp_variable_map );

void ST_layer_deriv( leafLayer * layer_i, std::list<leafLayer*>* layers, double t,

std::map<leafLayer*, std::vector<long double>*> & layer_temp_variable_map,

std::map<leafLayer*, std::vector<long double>*> & layer_derivative_map );

/**

* prints parameter value for the breakdown of this ST fungicide

* @return - value of this parameter

*/

double get_ST_breakdown(){

return this->ST_breakdown;

}

/**

* prints parameter value for the breakdown of this ST fungicide

* @return - value of this parameter

*/

double get_ST_uptake(){

return this->ST_uptake;

}

/**

* sets the new seed treatment fungicide reservoire amount

*

* @param val - new value for the amount of fungicide left

*/

void set_Nseed( double val ){

N_seed = val;

}

/**

* retrieves the value for the amount of fungicide remaining

* in the seed reservoire

*

* @return - the amount of fungicide remaining

*/

double get_Nseed(){

return N_seed;

}

/**

* Replenishes the dose of fungicide on the seed

*/

void replenish_ST_fungicide(){

N_seed = initial_dose;

std::cout<<"N_seed = "<<N_seed<<"\n";

std::cout<<"";

}

/**

* Returns the percentage of ST dose compared

* to the initial dose

*

* @return - ST percentage of intitial

*/

double get_ST_percent_of_initial(){

std::cout<<"N_seed "<<N_seed<<" initial_dose "<<initial_dose<<"\n";

return ( N_seed / initial_dose ) * 100;

}

protected:

double calc_seed_treat_concentration( leafLayer * layer_i, std::list<leafLayer*>* layers,

std::map<leafLayer*, std::vector<long double>*> & layer_temp_variable_map,

double t );

double N_seed;

double initial_dose;

};

#endif

**high_risk_foliar_times.txt**

1456 0.0

1700 0.0

**low_risk_foliar_times.txt**

1456 0.0

1700 0.0

**high_ST_input_dose.txt**

Dose 4.5

**low_ST_input_dose.txt**

Dose 0.0

**ST_in.txt**

LEAF_LAYER_BEGIN

NUMBER 1

AMAX 0.95

GROWTH_INI 1635

EMERGENCE 1700

SENES_INI 2725

DEATH 2928

END_LAYER

LEAF_LAYER_BEGIN

NUMBER 2

AMAX 1.05

GROWTH_INI 1513

EMERGENCE 1578

SENES_INI 2676

DEATH 2900

END_LAYER

LEAF_LAYER_BEGIN

NUMBER 3

AMAX 0.86

GROWTH_INI 1391

EMERGENCE 1456

SENES_INI 2408

DEATH 2590

END_LAYER

LEAF_LAYER_BEGIN

NUMBER 4

AMAX 0.76

GROWTH_INI 1269

EMERGENCE 1334

SENES_INI 2212

DEATH 2373

END_LAYER

LEAF_LAYER_BEGIN

NUMBER 5

AMAX 0.59

GROWTH_INI 1147

EMERGENCE 1212

SENES_INI 1968

DEATH 2094

END_LAYER

LEAF_LAYER_BEGIN

NUMBER 6

AMAX 0.43

GROWTH_INI 1025

EMERGENCE 1090

SENES_INI 1724

DEATH 1815

END_LAYER

LEAF_LAYER_BEGIN

NUMBER 7

AMAX 0.26

GROWTH_INI 903

EMERGENCE 968

SENES_INI 1480

DEATH 1536

END_LAYER

LEAF_LAYER_BEGIN

NUMBER 8

AMAX 0.26

GROWTH_INI 781

EMERGENCE 846

SENES_INI 1358

DEATH 1414

END_LAYER

LEAF_LAYER_BEGIN

NUMBER 9

AMAX 0.26

GROWTH_INI 659

EMERGENCE 724

SENES_INI 1236

DEATH 1292

END_LAYER

LEAF_LAYER_BEGIN

NUMBER 10

AMAX 0.23

GROWTH_INI 537

EMERGENCE 602

SENES_INI 1090

DEATH 1139

END_LAYER

LEAF_LAYER_BEGIN

NUMBER 11

AMAX 0.23

GROWTH_INI 415

EMERGENCE 480

SENES_INI 968

DEATH 1017

END_LAYER
