# Supplementary material for: The Evolution of Fungicide Resistance Resulting from Combinations of Foliar-Acting Systemic Seed Treatments and Foliar-Applied Fungicides: A Modeling Analysis
Source: PLoS One. 2016 Aug 29;11(8):e0161887. doi: 10.1371/journal.pone.0161887 (PMC5003396; doi:10.1371/journal.pone.0161887)
Supplement: S1 Table — (DOCX) [file pone.0161887.s003.docx]

**S1 Table. Estimated initial seed treatment dose (ST) resulting in % HAD losses (in year 1) similar to those achieved with a T1 foliar spray for a range of model scenarios.**

| Breakdown rate | Uptake model | Treatment type | % HAD loss |
| --- | --- | --- | --- |
| Low | Constant | T1 at 20 mg m^-2^ | 7.57 |
| Low | Constant | ST at 5.5 mg m^-2^ | 7.49 |
| Low | Transpiration | ST 2.15 mg m^-2^ | 7.60 |
| High | Constant | T1 at 20 mg m^-2^ | 7.97 |
| High | Constant | ST at 4.25 mg m^-2^ | 7.94 |
| High | Transpiration | ST at 2.15 mg m^-2^ | 7.50 |
