# Supplementary material for: The Evolution of Fungicide Resistance Resulting from Combinations of Foliar-Acting Systemic Seed Treatments and Foliar-Applied Fungicides: A Modeling Analysis
Source: PLoS One. 2016 Aug 29;11(8):e0161887. doi: 10.1371/journal.pone.0161887 (PMC5003396; doi:10.1371/journal.pone.0161887)
Supplement: S2 Table — (DOCX) [file pone.0161887.s004.docx]

**S2 Table. Estimated initial seed treatment dose (ST) leading to a 60% reduction in AUDPC for a range of model scenarios.** This reduction in AUDPC is consistent with that found for *Zymoseptoria tritici* spore washing data comparing treated and untreated winter wheat leaves as reported by Parker and Lovell [22]. AUDPC values were calculated according to infectious leaf tissue on all leaves between 500 and 1200 degree days.

| Dose | Uptake model | Breakdown rate | % AUDPC reduction due to treatment |
| --- | --- | --- | --- |
| 1 mg m^-2^ | Transpiration | Low | 60.83 |
| 1 mg m^-2^ | Transpiration | High | 62.00 |
| 1 mg m^-2^ | Constant | Low | 60.94 |
| 1 mg m^-2^ | Constant | High | 61.68 |
