# Supplementary material for: The Evolution of Fungicide Resistance Resulting from Combinations of Foliar-Acting Systemic Seed Treatments and Foliar-Applied Fungicides: A Modeling Analysis
Source: PLoS One. 2016 Aug 29;11(8):e0161887. doi: 10.1371/journal.pone.0161887 (PMC5003396; doi:10.1371/journal.pone.0161887)
Supplement: S3 Table — (DOCX) [file pone.0161887.s005.docx]

**S3 Table. Median selection ratios under three treatment regimes.** Selection ratios were calculated for individual growing seasons by dividing the frequency of the fungicide-resistance population in season *x+1* by that of season *x*. The frequency of resistance was set to 1E-05 at the beginning of the first growing season. The median ratio based on ten consecutive growing seasons is tabulated below. ST denotes the seed treatment, whereas T1 and T2 refer to foliar treatments applied at full emergence of eventual leaf 3 and the flag leaf, respectively.

| Treatment regime | Breakdown rate | Selection ratio |
| --- | --- | --- |
| Untreated | - | 1 |
| T1 only | low | 4.37 |
| ST only, with ST = T1 |  | 4.00 |
| ST only with ST = 60% AUDPC reduction |  | 2.26 |
| T1 only | high | 3.62 |
| ST only, with ST = T1 |  | 3.86 |
| ST only with ST = 60% AUDPC reduction |  | 2.26 |
