# Supplementary material for: The Evolution of Fungicide Resistance Resulting from Combinations of Foliar-Acting Systemic Seed Treatments and Foliar-Applied Fungicides: A Modeling Analysis
Source: PLoS One. 2016 Aug 29;11(8):e0161887. doi: 10.1371/journal.pone.0161887 (PMC5003396; doi:10.1371/journal.pone.0161887)
Supplement: S5 Table — (DOCX) [file pone.0161887.s007.docx]

**S5 Table. Effective lives (in years) for a seed treatment that provides the same level of control as a T1 foliar treatmentin the presence of the constant seed treatment uptake model and a high fungicide breakdown rate.**

| ST_dose | foliar_dose | ST_T1 | ST_T2 | ST_T1_T2 |
| --- | --- | --- | --- | --- |
| 0 | 0 | 0 | 0 | 0 |
| 0 | 0.2 | 0 | 0 | 0 |
| 0 | 0.4 | 0 | 0 | 0 |
| 0 | 0.6 | 0 | 0 | 5 |
| 0 | 0.8 | 0 | 0 | 5 |
| 0 | 1 | 0 | 0 | 5 |
| 0.2 | 0 | 0 | 0 | 0 |
| 0.2 | 0.2 | 0 | 0 | 5 |
| 0.2 | 0.4 | 0 | 0 | 4 |
| 0.2 | 0.6 | 0 | 0 | 4 |
| 0.2 | 0.8 | 4 | 0 | 4 |
| 0.2 | 1 | 4 | 5 | 4 |
| 0.4 | 0 | 0 | 0 | 0 |
| 0.4 | 0.2 | 0 | 0 | 5 |
| 0.4 | 0.4 | 4 | 0 | 4 |
| 0.4 | 0.6 | 4 | 5 | 4 |
| 0.4 | 0.8 | 4 | 5 | 4 |
| 0.4 | 1 | 4 | 5 | 4 |
| 0.6 | 0 | 0 | 0 | 0 |
| 0.6 | 0.2 | 0 | 0 | 4 |
| 0.6 | 0.4 | 4 | 5 | 4 |
| 0.6 | 0.6 | 4 | 5 | 4 |
| 0.6 | 0.8 | 4 | 5 | 4 |
| 0.6 | 1 | 4 | 5 | 3 |
| 0.8 | 0 | 0 | 0 | 0 |
| 0.8 | 0.2 | 4 | 0 | 4 |
| 0.8 | 0.4 | 4 | 5 | 4 |
| 0.8 | 0.6 | 4 | 5 | 4 |
| 0.8 | 0.8 | 4 | 5 | 3 |
| 0.8 | 1 | 4 | 4 | 3 |
| 1 | 0 | 0 | 0 | 0 |
| 1 | 0.2 | 4 | 4 | 4 |
| 1 | 0.4 | 4 | 5 | 4 |
| 1 | 0.6 | 4 | 5 | 4 |
| 1 | 0.8 | 4 | 4 | 3 |
| 1 | 1 | 4 | 4 | 3 |
