# Supplementary material for: The Evolution of Fungicide Resistance Resulting from Combinations of Foliar-Acting Systemic Seed Treatments and Foliar-Applied Fungicides: A Modeling Analysis
Source: PLoS One. 2016 Aug 29;11(8):e0161887. doi: 10.1371/journal.pone.0161887 (PMC5003396; doi:10.1371/journal.pone.0161887)
Supplement: S12 Table — (DOCX) [file pone.0161887.s014.docx]

**S12 Table. Effect of different ascospore initiation.** Effective lives (in years) for a seed treatment that has the same level of control as a T1 foliar treatment in the presence of the constant seed treatment uptake model and a low fungicide breakdown rate. In this scenario the ascospores initiating the epidemic in the next growing season were related to the infectious tissue densities on all leaf layers, not only the top 5 leaf layers.

| ST_dose | foliar_dose | ST_T1 | ST_T2 | ST_T1_T2 |
| --- | --- | --- | --- | --- |
| 0 | 0 | 0 | 0 | 0 |
| 0 | 0.2 | 0 | 0 | 0 |
| 0 | 0.4 | 0 | 0 | 5 |
| 0 | 0.6 | 0 | 0 | 5 |
| 0 | 0.8 | 0 | 0 | 5 |
| 0 | 1 | 0 | 0 | 5 |
| 0.2 | 0 | 0 | 0 | 0 |
| 0.2 | 0.2 | 0 | 0 | 5 |
| 0.2 | 0.4 | 4 | 4 | 4 |
| 0.2 | 0.6 | 4 | 5 | 4 |
| 0.2 | 0.8 | 4 | 5 | 4 |
| 0.2 | 1 | 4 | 5 | 4 |
| 0.4 | 0 | 0 | 0 | 0 |
| 0.4 | 0.2 | 4 | 0 | 4 |
| 0.4 | 0.4 | 4 | 5 | 4 |
| 0.4 | 0.6 | 4 | 5 | 4 |
| 0.4 | 0.8 | 4 | 5 | 4 |
| 0.4 | 1 | 4 | 4 | 4 |
| 0.6 | 0 | 0 | 0 | 0 |
| 0.6 | 0.2 | 4 | 5 | 4 |
| 0.6 | 0.4 | 4 | 5 | 4 |
| 0.6 | 0.6 | 4 | 5 | 4 |
| 0.6 | 0.8 | 4 | 4 | 3 |
| 0.6 | 1 | 4 | 4 | 3 |
| 0.8 | 0 | 0 | 0 | 0 |
| 0.8 | 0.2 | 4 | 5 | 4 |
| 0.8 | 0.4 | 4 | 5 | 4 |
| 0.8 | 0.6 | 4 | 4 | 3 |
| 0.8 | 0.8 | 4 | 4 | 3 |
| 0.8 | 1 | 4 | 4 | 3 |
| 1 | 0 | 0 | 0 | 0 |
| 1 | 0.2 | 4 | 5 | 4 |
| 1 | 0.4 | 4 | 4 | 4 |
| 1 | 0.6 | 4 | 4 | 3 |
| 1 | 0.8 | 4 | 4 | 3 |
| 1 | 1 | 4 | 4 | 3 |
